# Chitosan based smart injectable hydrogels for biomedical applications: A comprehensive review

**DOI:** 10.1016/j.bioactmat.2025.09.028

**Published:** 2025-10-24

**Authors:** Qamar Salamat, Rasoul Moradi, Zahra Nadizadeh, Pirouz Kavehpour, Mustafa Soylak, Ahmet Asimov, Md Zillur Rahman, Tomáš Kovářík, Václav Babuška, Kalim Deshmukh

**Affiliations:** a NanoBioAnalytical Chemistry Center, Khazar University, Baku, Azerbaijan; b Erciyes University, Faculty of Sciences, Department of Chemistry, Kayseri, Turkey; c KU-KIST Graduate School of Converging Science and Technology, Korea University, Seoul, South Korea; d Department of Mechanical and Aerospace Engineering, University of California, Los Angeles, California, United States of America; e Faculty of Chemistry, University of Tehran, Tehran, Iran; f Department of Bioengineering, University of California, Los Angeles, California, United States of America; g Technology Research and Application Center (ERU-TAUM), Erciyes University, Kayseri, Turkey; h Turkish Academy of Sciences (TUBA), Cankaya, Ankara, Turkey; i Composite Materials Scientific Research Center, Azerbaijan State University of Economics, Baku, Azerbaijan; j Department of Mechanical Engineering, Ahsanullah University of Science and Technology, Dhaka, Bangladesh; k New Technologies-Research Centre, University of West Bohemia in Pilsen, Pilsen, Czech Republic; l Department of Material Science and Metallurgy, Faculty of Mechanical Engineering, University of West Bohemia in Pilsen, Pilsen, Czech Republic; m Department of Medical Chemistry and Biochemistry, Faculty of Medicine in Pilsen, Charles University, Pilsen, Czech Republic

**Keywords:** Chitosan, Injectable hydrogels, drug delivery, Wound healing, Tissue engineering

## Abstract

Chitosan-based smart injectable hydrogels (CS-SIHs) have emerged as multifunctional platforms for drug delivery, regenerative medicine, and tissue engineering (TE), owing to their inherent biocompatibility, biodegradability, and responsiveness to external stimuli such as pH, temperature, and ionic strength. These smart hydrogels offer controlled, localized therapeutic release and mimic the extracellular matrix (ECM), thereby fostering cell adhesion, proliferation, and differentiation. In clinical applications such as bone regeneration, cartilage repair, and chronic wound healing, CS-SIHs can be encapsulated with various therapeutic agents, including proteins, nucleic acids, and small molecules, facilitating minimally invasive delivery. Recent studies have been more focused on developing CS-SIHs with enhanced bioactivity, mechanical integrity, and adaptability to dynamic microenvironments. This review provides an in-depth analysis of novel CS-SIH formulations and their potential therapeutic applications, as well as a comprehensive overview of recent preclinical and translational studies. Additionally, this investigation explores the challenges of clinical translation, including regulatory hurdles and scalability concerns. This work distinguishes itself by systematically integrating the physicochemical properties, intelligent response mechanisms, crosslinking strategies, and biomedical applications of CS-SIHs, offering a coherent framework for future research and development in the field of biomedical engineering.

## Highlights

• Describing Design Strategies for Chitosan Based Injectable Hydrogels.
• Discussing Physicochemical Properties of Chitosan Based Injectable Hydrogels.
• Reviewing the Biomedical Applications of Chitosan Based Injectable Hydrogels.
• Proposing Future Directions for the Development of Chitosan Based Smart Injectable Hydrogels for Biomedical Applications.

## Graphical abstract

**Figure:**
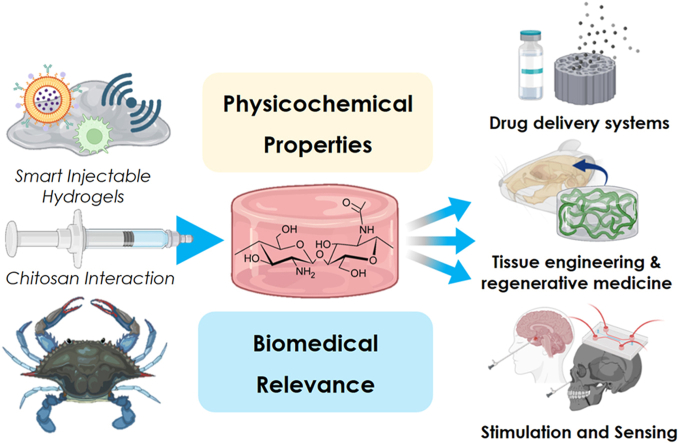

## 1 Introduction

In recent years, biomedical materials have witnessed remarkable advancements, particularly in developing smart injectable hydrogels (SIHs). These innovative materials are designed to go through a sol-gel phase transition in response to specific physiological or external stimuli, such as temperature, acidity, or salt levels. This unique characteristic allows SIHs to be administered least invasively, enabling in situ gelation at the target site. SIHs have substantial potential in biomedical applications, such as targeted drug delivery, wound healing, and tissue engineering (TE) [[1], [2], [3], [4], [5]]. Among the different smart hydrogel systems, those based on chitosan (CS) are very promising. CS, a naturally derived polysaccharide obtained through chitin deacetylation, is biocompatible, biodegradable, and non-immunogenic, which are highly desirable for biomedical use. CS-based smart injectable hydrogels (CS-SIHs) exhibit inherent advantages of CS, together with the stimulus-responsive properties of smart hydrogels, enabling site-specific drug delivery and tissue regeneration [6,7]. A distinctive feature of SIHs is their transformation from a liquid to a gel state under physiological conditions, facilitating precise localization and sustained release of therapeutic agents. The term "smart" underscores their ability to adapt to environmental stimuli, thus aligning well with the goals of precision and personalized medicine. Their injectable nature eliminates the need for invasive surgical procedures and enables spatially controlled delivery systems or scaffolding support at targeted anatomical sites [[8], [9], [10]]. Structurally, SIHs are three-dimensional (3D) polymer networks with a high-water retention capacity, closely mimicking the properties of natural tissue. This enables the use of SIHs in drug delivery systems (DDSs), where the gelation process facilitates localized and sustained release, thereby advancing the therapeutic efficacy with diminishing adverse side effects.

**Fig. 1:**
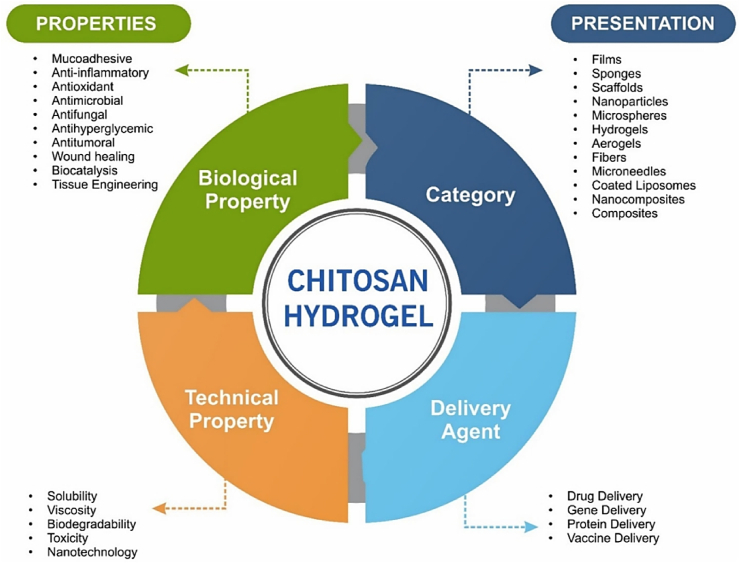
Multifaceted role of CS-based hydrogels in the biomedical field [11]. Copyright 2024. Reproduced with permission from Elsevier.

This review focuses particularly on how the fundamental physicochemical and biological properties of CS including solubility, degradability, and functional groups chemistry directly impact its performance in injectable, stimuli-responsive hydrogel systems. These hydrogels also function as scaffolds in TE, replicating the natural extracellular matrix (ECM) to support adhesion, proliferation, and tissue regeneration. The high adaptability and responsiveness render SIHs among the most promising choices in modern day biomedical engineering [[11], [12], [13]]. Fig. 1 illustrates various key characteristics of CS-based hydrogels [11].

CS-based hydrogels are exceptionally valuable owing to their biodegradability and biocompatibility, ensuring that they do not cause detrimental biological reactions and are safely excreted or metabolized by the body. These features make them broadly relevant for long-term biomedical applications such as sustained drug release, wound care, and regenerative therapies. Their injectability allows administration via syringes or catheters without requiring surgical procedures. Once administered, the hydrogels undergo gelation at the target site in response to physiological conditions [14,15]. The structural backbone of CS hydrogels comprises cross-linked polymer chains, which can be formed through physical (e.g., ionic interactions and hydrogen bonding) or chemical crosslinking (e.g., glutaraldehyde and genipin (GP)). Physical methods are usually preferred due to their simplicity and avoidance of toxic agents. The characteristics (e.g., mechanical strength, porosity, and swelling behaviour) of CS hydrogels can be finely tuned by changing the deacetylation degree, molecular weight, and crosslinking density, thus broadening their utility in various biomedical contexts [[16], [17], [18], [19], [20], [21]].

The multifunctionality of CS-SIHs stems from their customizable properties. Their capacity for high water absorption is advantageous for wound dressings and drug reservoirs. Their swelling behaviour can be precisely modulated by altering crosslink density or ambient pH. Additionally, the intrinsic antimicrobial activity of CS renders it highly effective for wound healing and infection control. CS-SIHs have shown success in the controlled delivery of anti-cancer agents, antibiotics, and growth factors, thereby improving therapeutic outcomes. In TE, they function as biocompatible scaffolds that assist in cellular regeneration, particularly in cartilage, bone, and skin tissue repair. When combined with other polymers or nanomaterials, their mechanical properties and biological performance can be further enhanced [[22], [23], [24], [25], [26], [27], [28], [29], [30]]. CS-SIHs also have applications in biosensing, where their structure can be functionalized with enzymes, antibodies, or aptamers to create highly sensitive and specific biosensors. Such systems have been utilized in medical diagnostics, food safety, and environmental monitoring. However, despite their vast potential, several challenges remain. Depending on the source, variability in CS properties can lead to inconsistencies in hydrogel properties. Moreover, CS-SIHs may not be strong enough for some applications, necessitating the development of hybrid or composite hydrogels with better properties.

CS-SIHs have evolved from fundamental biomaterials to multifunctional, precision-engineered systems employed in regenerative medicine, controlled drug delivery, and biosensing [[31], [32], [33]]. Their significant customizability, enabled by varying degrees of deacetylation, molecular weight, and crosslinking methods, has produced hydrogels with modifiable swelling, mechanical strength, and degradation properties [34,35]. Furthermore, their inherent antibacterial properties, mucoadhesive characteristics, and biocompatibility increase their applicability in intricate biomedical settings [36]. Recent advancements have broadened its application to the administration of anti-cancer drugs, growth factors, and bioactive chemicals for wound healing, cartilage regeneration, and targeted infection control [37]. Composite formulations that incorporate nanoparticles (NPs), growth factors, or other biopolymers have demonstrated enhanced efficacy in preclinical evaluations, particularly in targeted therapeutic release. Additionally, their responsiveness to different stimuli, including pH, temperature, and ionic strength, makes them suitable for varying physiological environments [23].

This review presents a comprehensive analysis of recent advancements in CS-SIHs, focusing on their design strategies, functional properties, preclinical applications, and emerging translational efforts. The review also discusses the challenges accompanying their clinical integration and regulatory preparedness and proposes future research directions to resolve these obstacles. While previous reviews have discussed injectable hydrogels or CS derivatives separately, they often lack a cohesive focus on multi-responsive CS-based injectable systems specifically designed for regenerative medicine and drug delivery. Many earlier works focus on fabrication techniques or individual stimulus-responsiveness, without integrating these aspects into a unified biomedical framework. In contrast, this review provides a systematic overview that includes CS's intrinsic properties, crosslinking methodologies, stimulus-responsive behaviors, scaffold fabrication strategies, and translational clinical applications, thus serving as a comprehensive resource for both researchers and clinicians.

## 2 Overview of smart injectable hydrogels

SIHs are a revolutionary advancement in biomedical materials that undergo a phase transition into a gel within the body when exposed to certain external stimuli while delivered in liquid form [38,39]. Their adaptability and practical characteristics make these hydrogels highly important in many biomedical applications that include drug delivery, regenerative medicine, and TE. The "smart" label is bestowed upon these hydrogels due to their capacity to respond to external stimuli or adapt to environmental changes, thereby significantly improving their efficiency and applicability in personalized medicine. Their injectability enables minimally invasive procedures, rendering them optimal for applications requiring precise localization, controlled release of therapeutic agents, or in situ induction of tissue growth and repair [[39], [40], [41]]. At their core, SIHs are fundamentally 3D polymeric networks which can absorb and retain substantial amounts of water, therefore being structurally analogous to human tissues. Their inherent environmental factors often cause the transformation from liquid to gel after being injected into the human body [41]. The hydrogel properties enable diverse applications, such as tailored DDSs, where they facilitate the localized and controlled release of therapeutics, advancing treatment efficacy and nullifying the adverse effects [[42], [43], [44]]. These hydrogels also function as scaffolds in TE that mimic the natural ECM, enabling cell adhesion, proliferation, and regeneration [[45], [46], [47]]. The adaptability and responsiveness of these SIHs make them a promising choice for next-generation medicine, with ongoing research continually expanding their potential.

### 2.1 Characteristics of SIHs

#### 2.1.1 Injectability

**Fig. 2:**
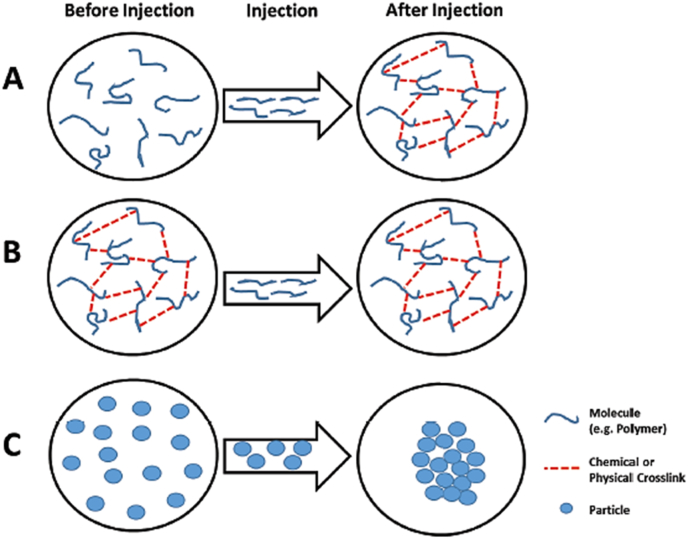
Schematic of three injectable hydrogel mechanisms: (A) liquids that gel in situ after injection, (B) shear-thinning gels that regain structure post-injection, and (C) particles in suspension that assemble into a gel after injection [49]. Copyright 2018. Adapted from Research in Molecular Medicine.

**Fig. 3:**
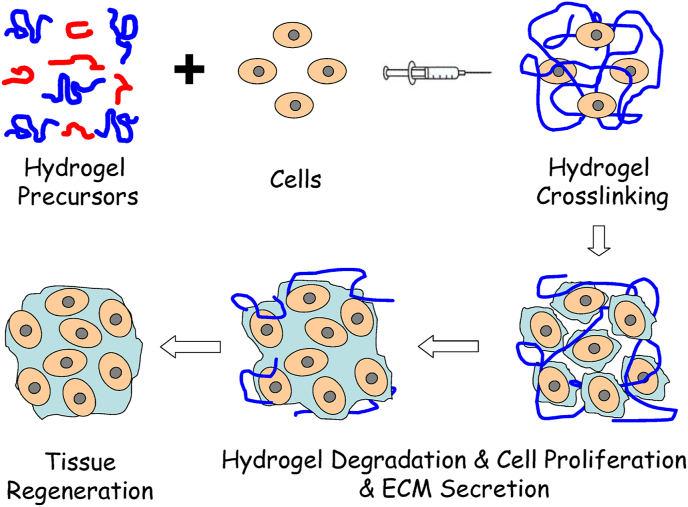
An injectable hydrogel designed for tissue regeneration involves isolating cells from a small biopsy, culturing them in vitro, and encapsulating them in hydrogel precursors. These precursors are then injected into the patient through a needle. The hydrogel provides initial structural support, retaining cells within the damaged tissue area, which aids in metabolism, cell proliferation, and the synthesis of new ECM. As the cells secrete ECM, the hydrogel gradually degrades. This approach allows minimally invasive transplantation of the combination of cells, growth factors, and hydrogel [50]. Copyright 2010. Adapted from MDPI.

The injectability of smart hydrogels is the most important characteristic, particularly in biomedical applications, as it enables targeted delivery with minimal invasiveness. This ability makes it possible to administer hydrogels in a liquid form by using a syringe, catheter, or other minimally invasive techniques. Upon administration, these materials respond to physiological parameters, including temperature, pH, or ionic concentration, triggering an in-situ sol-gel transition that leads to localized gel formation. This liquid-to-gel transition is especially advantageous for targeted therapies, as it facilitates the ability of the hydrogel to adapt to the complex structures of tissues or cavities and maintain its position in situ. Such adaptability ensures that the active therapeutic agents are delivered precisely and retained effectively, enhancing treatment efficacy. Because of their high injectability, smart hydrogels are particularly well-suited for applications requiring repeated administration, such as the sustained delivery of therapeutics for chronic diseases. Furthermore, their liquid state before injection ensures that they are suitable for use in intricate anatomical locations, particularly those that are difficult to access using traditional surgical methods [48]. Three different injectability mechanisms include in-situ gelling liquids, injectable gels, and injectable particles, as demonstrated in Fig. 2 [49]. Another example of injectable hydrogels used in tissue regeneration approaches is shown in Fig. 3 [50]. Injectable hydrogels have a microstructure resembling the ECM, promoting effective physical integration into the defect site. This eliminates the need for open surgery, enabling the use of the least invasive techniques for cell delivery. The encapsulated cells proliferate within the hydrogel, subsequently secreting new ECM to repair the damaged tissue [50].

#### 2.1.2 Biocompatibility

Biocompatibility is essential for any material used in biomedical applications, including SIHs. Intravenous administration of biologically compatible hydrogels reduces the risk of immunological rejection or harmful inflammatory reactions—an essential factor for ensuring long-term stability while minimizing damage to surrounding tissues or unwanted immunological responses. A deliberate selection of naturally biocompatible or chemically modifiable polymers to enhance compatibility is essential for developing intelligent hydrogels. CS, alginate, and polyethylene glycol (PEG) are the most extensively used polymers, widely recognized for their biocompatibility. Furthermore, intelligent hydrogels can be engineered to incorporate bioactive compounds that promote cellular infiltration and tissue regeneration, enhancing their integration with surrounding tissues. Regarding pharmaceutical delivery systems, hydrogel biocompatibility is crucial to ensure safe degradation and elimination from the body after fulfilling its therapeutic function [[51], [52], [53]].

**Fig. 4:**
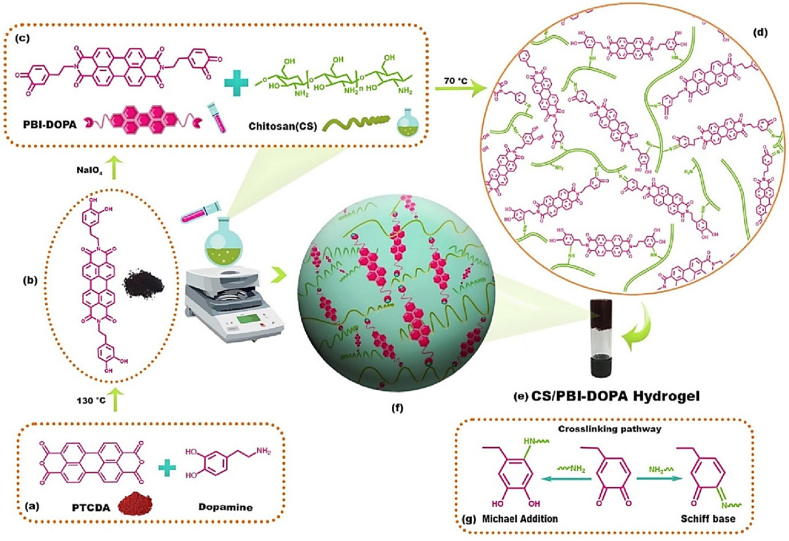
Synthesis of cross-linked CS/PBI-DOPA hydrogels. (a) Reaction demonstrating interaction between PTCDA and dopamine, (b) Structure of PBI-DOPA, (c) Interaction between oxidized PBI-DOPA and CS chains via crosslinking (d) Structure of the photosensitive CS/PBI-DOPA hydrogel, (e) Digital photo of the hydrogel, (f) Schematic diagram of the hydrogel, and (g) cross-linking mechanism [54]. Copyright 2023. Reproduced with permission from Elsevier.

A CS-based hydrogel (CS/PBI-DOPA) was made to increase the photodynamic properties of perylene for cancer treatment [54]. The hydrogel was fabricated employing an oxidative cross-linking method, in which the catechol groups of PBI-DOPA were oxidized to quinones, resulting in covalent bonding with the amino groups of CS via Schiff base reactions or Michael addition (Fig. 4) [54]. The resulting hydrogel showed shear-thinning behavior, a biocompatible microporous structure, and significantly improved singlet oxygen quantum efficiency. In vitro experiments comparing CS/PBI-DOPA hydrogel, free PBI-DOPA, and perylene tetracarboxylic dihydride (PTCDA) on breast cancer (MDA-MB-231), skin cancer (A375), and normal fibroblast cells demonstrated the superior phototoxicity of hydrogels against cancer cells while reducing toxicity in normal cells. Covalent conjugation of perylene to the hydrogel matrix mitigated self-quenching and molecular aggregation, increasing ROS generation for efficient photodynamic therapy (PDT). Overall, the prepared hydrogel is a biocompatible, elastic, and injectable technology with considerable potential for perylene-based drug delivery and improved cancer therapeutic outcomes [54].

#### 2.1.3 Mechanical properties

The mechanical characteristics of SIHs are crucial to their performance in TE and other biomedical uses. Key parameters, such as elasticity, rigidity, and strength, are essential for reproducing the natural ECM, which provides structural cues that support cellular motility, proliferation, and differentiation. Smart hydrogels must possess mechanical properties comparable to the substrates for optimal tissue regeneration, especially in load-bearing tissues (e.g., bone or cartilage). In addition, these mechanical properties need to be tunable to suit specific biomedical contexts. In particular, hydrogels utilized in soft tissue healing require a degree of flexibility and elastic modulus, whereas those designed for bone regeneration necessitate increased stiffness and hardness. Furthermore, hydrogels can dynamically change their mechanical properties responding to external stimuli, and adapting to the evolving physiological environment within the body [55,56].

**Fig. 5:**
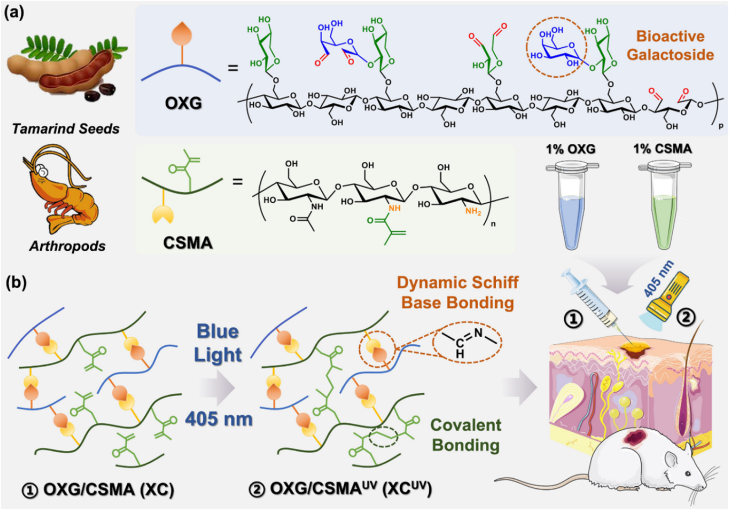
Synthesis pathway of injectable CS/xyloglucan composite hydrogels designed for skin wound treatment in a mouse model [57]. Copyright 2023. Reproduced with permission from Elsevier.

In a study by Wei et al., a bioactive and injectable hydrogel, composed entirely of natural polysaccharides, was developed to foster skin wound healing in a mouse model [57]. The hydrogel was developed through a dual cross-linking network comprising a reversible and dynamic network created by Schiff base reactions between aldehyde-containing xyloglucan (OXG) and methacrylate CS (CSMA) (Fig. 5a) alongside a secondary covalent network formed through photo-initiated polymerization of methacrylate groups (Fig. 5b) [57]. This innovative design imparted self-healing and shear-thinning capabilities, enabling facile injection, in situ gelation, and mechanical flexibility suited for clinical applications. Xyloglucan, extracted from tamarind seeds, consists of galactoside units known for their intrinsic anti-inflammatory, mucoadhesive, and cell-aggregating properties. These natural galactosides greatly improved cell spheroid formation, collagen deposition, re-epithelialization, angiogenesis, and hair follicle regeneration, thus accelerating wound healing. The electrophilic amino groups of CS and the inherent properties of xyloglucan provided the hydrogel with antibacterial and hemostatic properties [57]. In vitro and in vivo tests revealed that the hydrogel quickly accelerated wound healing compared to clinically used polyurethane-based dressings (e.g., Tegaderm). The hydrogel's efficacy is primarily attributed to its capacity to dynamically respond to mechanical and metabolic changes during tissue regeneration. Moreover, the photo-cross-linked secondary network provided an elastic, supportive 3D microenvironment suitable for deep wound tamponade and repair. The authors investigated the bioactivity of endogenous galactosides in developing injectable polysaccharide hydrogels for wound healing. Its remarkable cost-effectiveness, biodegradability, and biocompatibility highlight its potential for clinical translation. By harnessing the intrinsic bioactivity of natural materials, this work offers a sustainable and effective alternative to pharmacologically loaded dressings for tissue regeneration and wound healing [57].

#### 2.1.4 Degradability

One critical feature of SIHs is their ability to break down under controlled settings and in particular, this aspect is significant in drug delivery and TE applications, where temporary scaffolds or controlled release mechanisms are essential. Degradable hydrogels can effectively prevent the accumulation of toxic by-products in the body, mitigating long-term adverse effects. This is accomplished through their progressive breakdown in reaction to enzymatic activity or other environmental factors, such as pH fluctuations. The degradation rate can be accurately regulated to align with specific biomedical requirements. In TE, for instance, the hydrogel scaffold should degrade in tandem with tissue regeneration, ensuring continued structural support until the newly formed tissue is functionally mature. In DDSs, the release rate of the therapeutic agent is often synchronized with the hydrogel's degradation rate, allowing for sustained and controlled release over time. By enabling precise control over their degradation kinetics, SIHs offer remarkable versatility, making them highly valuable across diverse biomedical applications [58,59].

#### 2.1.5 Stimuli-responsiveness

**Fig. 6:**
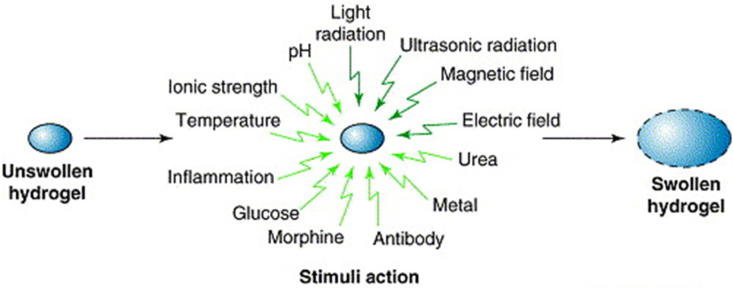
Stimuli-responsive swelling of hydrogels [62]. Copyright 2002. Reproduced with permission from Elsevier.

The "smart" features of hydrogels come from their ability to undergo chemical or physical transformations in response to external stimuli. Common triggers include variations in temperature, acidity, luminosity, and the presence of specific ions or proteins [59]. For example, certain thermosensitive hydrogels remain liquid at ambient temperature but rapidly transition into a gel upon exposure to physiological warmth. Similarly, pH-sensitive hydrogels can respond to changes in their surrounding chemical environment, regardless of whether acidic or alkaline, making them particularly beneficial for targeted drug delivery in regions with fluctuating pH levels, including tumor microenvironments or gastrointestinal tracts [60]. Due to their adaptability to specific stimuli, hydrogels are a highly suitable choice for developing personalized therapeutic strategies. The ideal performance of hydrogels in biomedical applications is credited to their ability to provide precise and regulated delivery of highly concentrated therapeutics [60,61]. The stimulus-responsive behavior of hydrogels is illustrated in Fig. 6 [62]. The external stimuli can be produced using various stimuli-generating devices, while internal stimuli can be created within the body to control the structural variations in the polymeric network exhibiting the desired drug release [62]. Compared to other polymers, such as collagen, silk fibroin, hyaluronic acid (HA), alginate, and PEG, CS-SIHs offer distinct medicinal advantages. These include intrinsic antibacterial properties, enhanced mucoadhesiveness, the ability to disrupt epithelial tight junctions, and pronounced hemostatic effects. The economic viability and chemical versatility of CS are further underscored by its abundant amino and hydroxyl groups, which facilitate straightforward derivatization. Table 1 provides a comparative analysis of various biomaterials, emphasizing the unique benefits of CS in drug delivery, wound healing, and regenerative medicine applications.

**Table 1:** Comparison of properties between CS and other injectable hydrogels.

Property	CS	Collagen	Silk Fibroin	HA	Alginate	PEG	Ref.
**Biocompatibility**	Excellent	Excellent	Excellent	Excellent	Excellent	Excellent	[[63], [64], [65], [66]]
**Biodegradability**	Enzymatically degraded by lysozyme	Enzymatically degraded (collagenase)	Biodegradable (protease-sensitive)	Enzymatically degraded by hyaluronidase	Enzymatically degraded (alginate lyase)	Non-biodegradable (unless modified)	[64,65,67,68]
**Antimicrobial Activity**	Strong intrinsic activity	None (requires additives)	Limited (needs functionalization)	None (requires silver/zinc additives)	None (requires additives)	None (requires functionalization)	[64,65,69,70]
**Mucoadhesiveness**	High (cationic interactions)	Low	Low	Low–Moderate	Moderate (anionic interactions)	None	[64,[71], [72], [73]]
**Tight Junction Permeability**	Opens tight junctions	Not reported	Not reported	Weakly reported	Limited	Does not affect tight junctions	[65,66,74,75]
**Stimuli Responsiveness**	pH, temperature, and enzyme	Enzyme (mainly collagenase)	Limited pH and enzyme sensitivity	Mainly enzyme and pH responsive	Mainly ion and pH responsive	Thermo- and photo-responsive (when modified)	[68,[76], [77], [78], [79]]
**Mechanical** **Strength**	Tunable (moderate to strong)	Good, limited to load-bearing without crosslinking	Strong (suitable for mechanical support)	Weak (requires reinforcement)	Low mechanical strength unless modified	Tunable (depends on formulation)	[64,68,78,80]
**Injectability**	Excellent	Good (after solubilization)	Good (requires processing)	Excellent	Excellent (gelation with Ca^2+^)	Excellent	[64,65,78,81]
**Cost & Availability**	Low (abundant from seafood waste)	Moderate to high	High (silkworms, purification steps)	High (fermentation-based or animal-derived)	Low (abundant, plant-based)	Moderate to high (synthetic)	[64,65,82]
**Chemical Modifiability**	Highly modifiable (–NH_2_, –OH groups)	Limited modifiability	Modifiable (–COOH, –NH_2_ groups)	Modifiable (–COOH groups)	Limited (–COOH groups)	Highly modifiable (wide functionalization)	[65,66,79,83,84]
**Hemostatic Properties**	Strong clotting ability	Moderate	Weak	None	Moderate (forms gels but not strong clotting)	None	[64,73,85]

### 2.2 Mechanisms of smart behaviour in injectable hydrogels

SIHs have emerged as a probable option for biomedical applications, particularly in tissue regeneration and targeted drug delivery, due to their ability to respond dynamically to external stimuli. SIHs can alter their physical condition through their inherent sensitivity to stimuli or adapt their characteristics to more effectively respond to therapeutic environments. The high versatility of SIHs is attributed to their stimuli-responsive mechanisms, which enable a wide array of controlled and targeted treatment techniques. This section includes a comprehensive analysis of four fundamental mechanisms in SIHs, namely pH-responsive, thermo-responsive, photo-responsive, and enzyme-responsive behaviors, along with their respective biomedical applications. The intelligent functionality of SIHs is driven by their capacity to detect and react to diverse stimuli, including pH, temperature, enzymes, and light. The stimuli-responsive mechanisms of hydrogels allow them to carry out predetermined functions, such as drug administration or tissue regeneration, with precise control and targeting. Thermo-responsive hydrogels (TRHs) undergo sol-gel transitions triggered by temperature changes, making them ideal for in situ gelation. pH-responsive hydrogels (pHRHs) exploit variations in acidity to enable controlled drug release at targeted sites. Enzyme-responsive hydrogels (ERHs) react to particular enzymes to cause localized degradation, allowing localized and condition-dependent therapeutic action. Photo-responsive hydrogels (PRHs) use light as an external stimulus to achieve spatiotemporal precision in activation and function. Each of these mechanisms provides a distinct edge for biomedical use, contributing to the high adaptability and functional potential of SIHs [86,87].

#### 2.2.1 Thermo responsive mechanisms

**Fig. 7:**
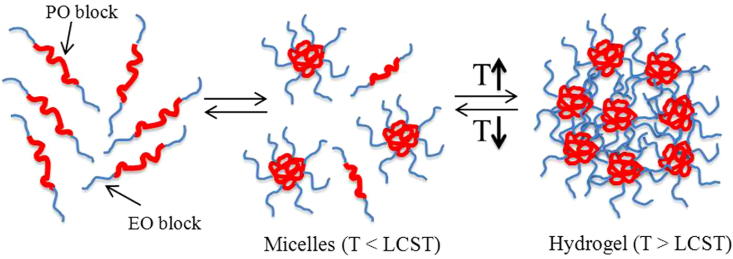
Micellization process and gel formation of aqueous poloxamer solution [91]. Copyright 2014. Reproduced with permission from Elsevier.

TRHs are a category of intelligent materials that experience phase transitions, such as sol-gel (liquid to solid) or gel-sol (solid to liquid), in response to temperature variations [88,89]. Typically, these hydrogels are formulated to stay in a liquid state at ambient temperature, facilitating ease of injection, and subsequently solidify into a gel upon exposure to physiological temperature (∼37 °C). The sol-to-gel transition is influenced by temperature-induced changes in the balance of hydrophobic and hydrophilic interactions within the polymer network [90,91]. TRHs demonstrate hydrophilicity and maintain a liquid state at temperatures below a critical threshold. However, polymers exhibit increased hydrophobicity when they exceed a certain lower critical solution temperature (LCST), thereby removing water and forming a gel as depicted in Fig. 7 [91]. This reversible phase transition is influenced by the interplay between polymer-polymer and polymer-solvent interactions.

TRHs have considerable potential in medication delivery systems. For instance, they may be administered intravenously in a liquid state and then gelate in situ at body temperature, forming a depot for sustained drug release. An example is hydrogels based on poly (N-isopropyl acrylamide) (PNIPAAm), which exhibit an LCST near body temperature. These hydrogels rapidly transition to a gel state upon intravenous administration, enabling controlled and prolonged release of therapeutic agents [[88], [89], [90], [91]]. TRHs are also employed in post-operative therapies, where precise, localized, and prolonged drug delivery is crucial for wound healing and infection prevention. For instance, TRHs can deliver drugs directly to a surgical site, releasing them gradually over days or even weeks. Moreover, TRHs are being actively explored for applications in cancer therapy, where the slightly elevated temperature of tumor tissues relative to healthy tissues can be used for site-specific drug release. This enables the precise intertumoral delivery of therapeutics, thereby minimizing collateral damage to healthy tissues [[88], [89], [90]].

**Fig. 8:**
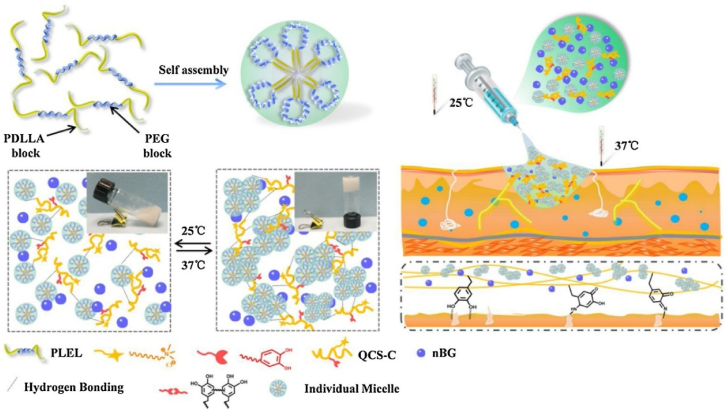
Injectable PLEL-nBG-QCS-C composite hydrogels for wound healing [92]. Copyright 2020. Reproduced with permission from Elsevier.

Zheng et al. produced a new injectable, thermo-sensitive hydrogel comprising catechol-modified quaternized CS (QCS-C), poly (d, l-lactide)-poly (ethylene glycol)-poly (d, l-lactide) (PLEL) and nano bioactive glass (nBG) [92]. The QCS-C modification reduced the LCST of PLEL-nBG-QCS-C hydrogels, promoting gelation under physiological environments while significantly enhancing tissue adhesion and antibacterial properties. Incorporating nBG provided bioactive ions that stimulated endothelial cell migration and angiogenesis, thereby considerably accelerating wound healing (Fig. 8) [92]. The hydrogel demonstrated superior mechanical and adhesive properties, making it well-suited for sealing ruptured skin and treating complex wounds. In vivo experiments confirmed its biocompatibility, bioactivity, and capacity to enhance wound healing. The bioactive ions produced by nBG facilitated blood vessel development and enhanced the expression of the VEGF and b-FGF genes. After seven days, wounds treated with PLEL-nBG-QCS-C hydrogel showed substantially improved healing results compared to conventional treatments such as fibrin glue or sutures. This injectable hydrogel adhesive offers rapid and effective wound closure and can polymerize at body temperature. Its diverse multifunctionality—combining antimicrobial action, strong adhesion, and pro-angiogenic properties—positions it as a promising therapeutic alternative, particularly for treating complex and difficult-to-manage skin injuries [92].

**Fig. 9:**
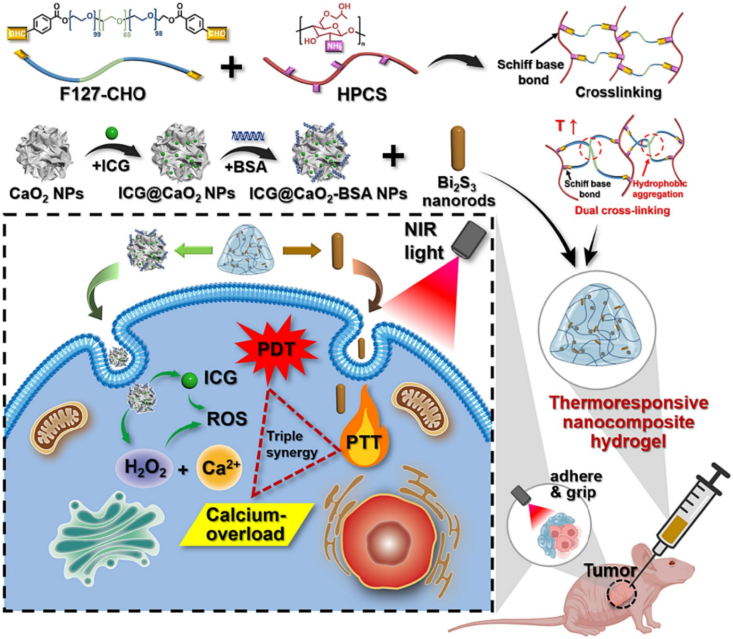
Nanocomposite TRHs comprising ICG@CaO_2_-BSA NPs and Bi_2_S_3_ nanorods for tumor therapy application involving calcium overload, photodynamic, and photothermal effects [93]. Copyright 2024. Reproduced with permission from Elsevier.

A unique self-healing, injectable hydrogel was designed by Qian et al. for synergistic tumor therapy, incorporating calcium overload, photodynamic, and photothermal processes [93]. This multifunctional hydrogel contains indocyanine green (ICG), bismuth sulfide (Bi_2_S_3_) nanorods, and calcium peroxide (CaO_2_) NPs functionalized using bovine serum albumin (BSA) (ICG@CaO_2_-BSA). The hydrogel was manufactured using dynamic Schiff-base bonds between aldehyde-modified Pluronic F127 (F127-CHO) and HPCS, allowing in situ injectability at room temperature (RT). The system displays strong adhesion to tumor tissues via hydroxyl groups and hydrophobic aggregation due to the thermoresponsive nature of F127-CHO (Fig. 9) [93]. When exposed to near-infrared radiation, ICG@CaO_2_-BSA NPs release Ca^2+^ and H_2_O_2_, generating ROS in the acidic tumor microenvironment. Concurrently, the synergistic interaction between Bi_2_S_3_ nanorods and ICG induces localized hyperthermia, facilitating targeted tumor cell ablation via photothermal effects. This device enables a triple-modal therapeutic strategy through a single minimally invasive injection and external NIR activation, ensuring sustained, controlled release of therapeutic agents. Remarkably, this approach achieved complete tumor ablation within one week without the need for conventional chemotherapy drugs and with no significant side effects or evidence of drug resistance. This work presents a novel, potentially useful, and safe technique to treat cancer [93].

In another work, Bhuiyan et al. successfully developed and optimized thermoresponsive CS/β-glycerophosphate (CS/β-GlyP) hydrogels for brain tissue regeneration [94]. Variations in CS (0.5–2 %) and β-GlyP (2–3 %) in the hydrogels revealed that formulations with 0.5 % CS/3 % β-GlyP (CH6) and 0.75 % CS/3 % β-GlyP (CH7) show the most promising results. These hydrogels exhibited rapid gelation (3–5 min) at physiological conditions (37 °C, pH 7.4) while maintaining injectability at 25 °C. Their porous microstructure and controlled biodegradation make them suitable for TE applications. Moreover, in vitro investigations using PC12 cells confirmed the hydrogels' high cell viability and proliferation, highlighting their biocompatibility. The distinctive architecture of hydrogels allows for the direct injection of stem cells and other therapeutics into the brain, promoting tissue regeneration. The cytocompatibility of the formulations, particularly those with CH6 and CH7, was further assessed through fluorescence imaging of viable and non-viable cells. The thermoresponsive CS/β-GlyP hydrogels, specifically CH6 and CH7, exhibit significant potential in brain TE, driven by three key advantages: cellular division potential, injection capability, and rapid gelation. However, further in vivo investigations were not carried out to ascertain the efficacy of these hydrogels in specific cerebral injury or pathology models [94].

#### 2.2.2 pH-responsive mechanisms

pH-responsive hydrogels (pHRHs) are engineered to modify their swelling characteristics in precise response to variations in pH in their surroundings. The polymer frameworks of these hydrogels contain ionizable functional groups, including carboxyl or amino groups, which can either accept or donate protons depending on the pH. The hydrogel swelling is attributed to an increase in electrostatic repulsion across polymer chains at a specific pH induced by the charging of these functional groups. Conversely, the hydrogel collapses when the pH shifts and the functional groups lose their charge. This dynamic swelling and deswelling behaviour render pHRHs particularly suitable for applications where pH varies across various body areas, such as the gastrointestinal tract [95,96]. A key application of pHRHs is oral pharmaceutical delivery systems. The gastrointestinal tract has different pH values, ranging from severely acidic (pH 1.5–3.5) in the stomach to neutral or mildly basic (pH 6–7.5) in the small intestine. A pHRH can be designed to maintain its integrity in the acidic circumstances of the stomach, releasing the drug only when it reaches the more neutral pH of the intestines. This targeted delivery method is especially beneficial for drugs that would otherwise degrade in gastric acid or for treating symptoms localized to the intestines, such as inflammatory bowel disease (IBD). Apart from its gastrointestinal applications, pHRHs are also being explored for their potential in cancer treatments. Tumor microenvironments often exhibit more acidic conditions than healthy tissues, making it possible to design pHRHs that selectively release drugs under these conditions. This selective release strategy helps protect the healthy cells while enhancing the therapeutic efficacy of the drug. In addition, pHRHs can be integrated into wound dressings that enable the controlled release of drugs or bioactive compounds in response to the pH changes associated with wound healing or infection [[95], [96], [97]].

**Fig. 10:**
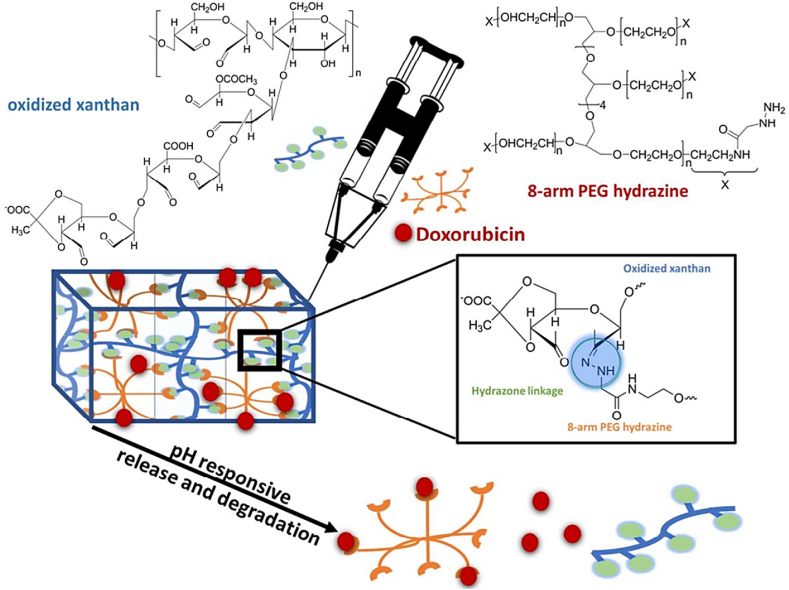
Hydrogel formation using hydrazone bonds between oxidized xanthan and 8-arm PEG hydrazine, enabling pH-sensitive and controlled release of DOX [98]. Copyright 2018. Reproduced with permission from the American Chemical Society.

A self-healing, pH-responsive, injectable hydrogel with "double H-bond" capabilities was presented by Sharma et al. [98]. This was accomplished by utilizing hydrazone bonds to link polysaccharides, such as 8-arm PEG hydrazine and oxidized xanthan (Fig. 10) [98]. Drug release studies, particularly those employing hydrogels loaded with doxorubicin (DOX), have revealed significant differences in drug release rates between the tumor pH (5.5) and the normal body pH (7.4), with accelerated drug release at the lower pH typically found in tumors. The MTT assay showed that oxidized xanthan and PEG hydrazine did not exhibit cytotoxic effects on human cells (NIH-3T3). Additionally, the live/dead assay highlighted the excellent cytocompatibility of the hydrogels in both two-dimensional (2D) and 3D growth settings, as accomplished by using encapsulated cells [98].

#### 2.2.3 Enzyme responsive mechanisms

ERHs are engineered to undergo degradation or swelling in response to specific enzymes. These hydrogels comprise polymer chains that are susceptible to cleavage or modification by enzymes intrinsic to the body or present in diseased tissues. By integrating peptide sequences or other enzyme-specific cleavage sites within the polymer structure, ERHs exhibit targeted responsiveness. When the specific enzyme interacts with the hydrogel, it identifies and degrades specific sequences, thereby modifying the physical characteristics of the hydrogel or inducing its disintegration. The enzyme-driven nature of the hydrogel enables precise regulation of its degradation, making it suitable for applications requiring either targeted or controlled disintegration [[99], [100], [101], [102]].

ERHs show great potential in DDSs, particularly for precise cancer treatments. Oncogenic tissues often produce elevated levels of enzymes, including matrix metalloproteinases (MMPs), which contribute to tumor progression and metastasis. Hydrogels that undergo degradation in the presence of MMPs can be employed to directly deliver anti-cancer therapy to the tumor site. Upon interaction with the enzymes generated by the tumor, the hydrogel undergoes degradation, resulting in the regulated and targeted release of the medication. Employing this focused approach minimizes adverse systemic side effects and enhances the therapeutic effectiveness of the therapy [101]. ERHs are also being formulated for TE, where controlled degradation is essential for the timely release of growth factors or other biologically active molecules. In tissue regeneration, the hydrogel scaffold can degrade in response to enzymes secreted by the regenerating tissue. This ensures the scaffold's support until the tissue has sufficiently healed and the scaffold is no longer necessary [99,100,102].

#### 2.2.4 Photo responsive mechanisms

**Fig. 11:**
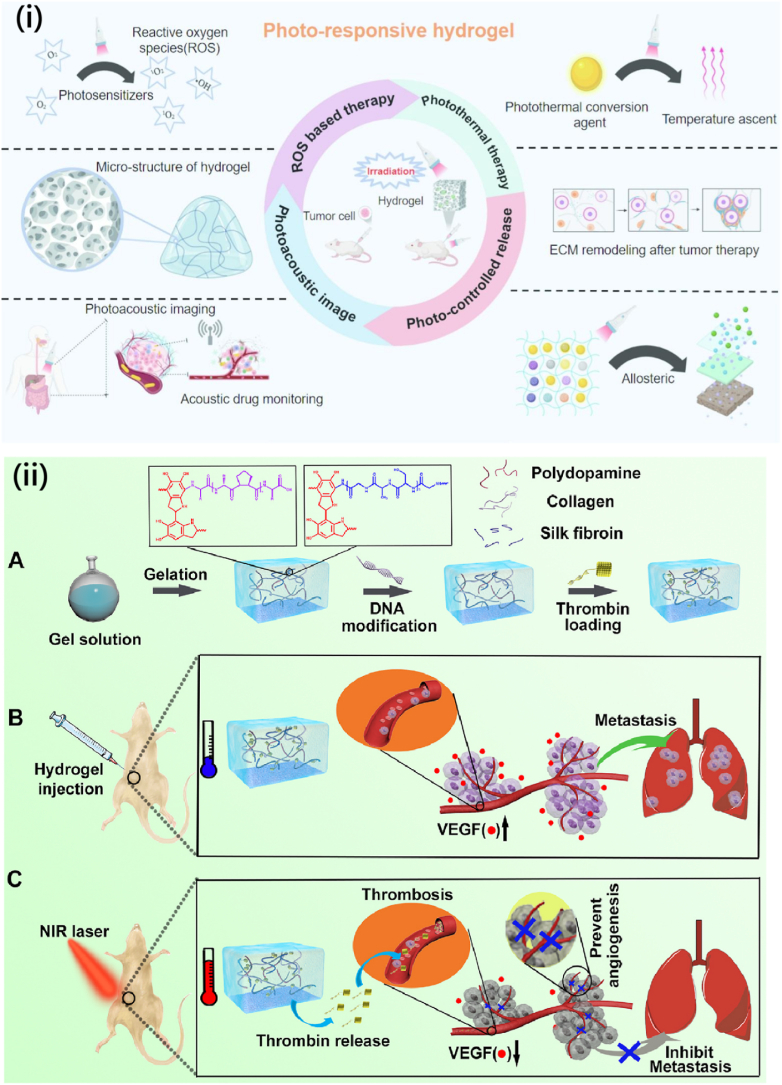
(I) Applications and basic biomedical properties of PRHs for tumor therapy [104]. Copyright 2024. Reproduced with permission from Elsevier, (ii) Illustration of photo-responsive injectable hydrogel construction and its working mechanism in nutrition deprivation to prevent metastasis and cancer recurrence [106]. Copyright 2021. Reproduced with permission from Elsevier.

PRHs integrate light-sensitive groups capable of undergoing chemical or structural modifications upon exposure to particular light wavelengths. Typically, these hydrogels are engineered with photo-labile links or photochromic molecules that can fracture or change their structure when exposed to ultraviolet (UV) or visible light. The specific design of these hydrogels enables them to undergo swelling, contraction, or degradation upon exposure to light. PRHs offer significant benefits for applications requiring precise control, as they allow for manipulating the hydrogel's spatial and temporal properties through light-induced changes. PRHs have extensive applications in light-controlled DDSs. In these systems, a therapeutic agent is encapsulated within the hydrogel matrix and is released only when the hydrogel is exposed to laser light. On-demand drug release technology enables the patient or doctor to precisely regulate the timing and localization of drug administration by delivering light directly to the intended area. This technique is particularly promising for cancer therapies, where light can be used to trigger the release of drugs precisely at the tumor site, thereby minimizing damage to surrounding healthy tissues [103,104]. PRHs are also being explored for their potential in dynamic TE scaffolds. By selectively exposing certain hydrogel areas to light, researchers may control the mechanical characteristics or degradation of the scaffold, thus guiding tissue formation more precisely. Moreover, these hydrogels show great potential in wound healing, where the healing process can be used to cause the release of medicinal drugs or growth factors under the control of light [[103], [104], [105], [106]]. Applications and essential biomedical properties of PRHs for tumor therapy are demonstrated in Fig. 11i [104]. In another work, Wang et al. [106] developed a photo-responsive injectable hydrogel employing collagen/silk fibroin composite cross-linked with polydopamine to deliver thrombin for blood vessel blockage and angiogenesis inhibition (Fig. 11(ii)) [106]. Upon injection into tumor-resected sites in mice, the hydrogel, activated by near-infrared light, releases thrombin for blood coagulation and suppresses angiogenesis-promoting signals. This strategy effectively hampers recurrence and metastasis in triple-negative breast cancer [106].

### 2.3 Different types of smart hydrogels

The utility and potential applications of SIHs in drug delivery and TE are contingent upon their ability to respond to a predetermined set of stimuli. Hydrogels are of three types based on their responsiveness: single-responsive, dual-responsive, and multi-responsive materials. Each category offers a distinct set of benefits, which vary depending on the complexity of the environmental conditions in which they are applied. Although single-responsive hydrogels provide a direct and accurate response to certain stimuli, dual-responsive hydrogels (DRHs) have the potential to adapt and self-regulate, making them appropriate for more complex applications. Developing multi-responsive hydrogels (MRHs) is the pinnacle of biomedical hydrogel technology, holding the most promise for advanced therapeutic applications, such as TE and targeted drug delivery. Despite the additional production challenges, MRHs offer the most significant potential in these fields [107,108].

#### 2.3.1 Single-responsive hydrogels

Single-responsive hydrogels (SRHs) are specifically engineered to detect and respond to a single stimulus, including pH, temperature, or light. These hydrogels exhibit tailored properties, undergoing physical or chemical transformations in response to a specific environmental stimulus, making them both straightforward and efficient for specific applications. Upon exposure to the particular stimulus, SRHs can experience phase transitions, swelling, or degradation, which can be harnessed for regulated drug release or structural modifications in biomedical contexts. The design and synthesis of SRHs are typically less intricate, resulting in increased stability and improved control over specific parameters. However, their limited responsiveness can hinder their ability to adapt to multifaceted biological environments, where multiple factors may need to be simultaneously considered. A notable example of a single-responsive hydrogel is TRHs based on poly (N-isopropyl acrylamide) (PNIPAAm). The LCST of PNIPAAm hydrogels is approximately 32 °C, which is near human body temperature. Below the LCST, the hydrogel retains a swollen, hydrated state; however, as the temperature surpasses the LCST, the hydrogel experiences a sol-gel transition, contracting into a denser configuration. This temperature-sensitive behavior makes PNIPAAm hydrogels particularly suitable for applications, including targeted drug delivery. For instance, the hydrogel can be administered into the body in its liquid form and subsequently gel at body temperature, thereby enabling the controlled release of therapeutics [78,109].

**Fig. 12:**
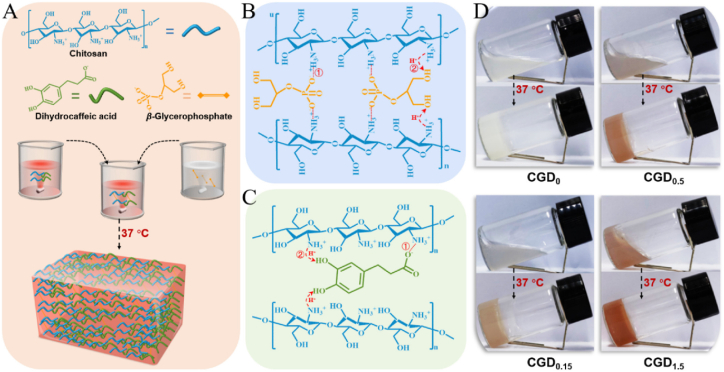
Fabrication of CGD TRHs. (A) Synthesis of hydrogel. (B) Proposed molecular mechanisms for CS thermosensitive gelation caused by GlyP and dihydrocaffeic acid, (C) at physiological temperatures (e.g., proton transfer and electrostatic attraction). (D) Images showing the hydrogel gelation precursor solutions at 37 °C [110]. Copyright 2023. Reproduced with permission from Elsevier.

Liu et al. recently produced thermosensitive hydrogels composed of CS and β-GlyP, incorporating varying dihydrocaffeic acid (DHCA) contents, and examined their structural, physical, biosafety, and hemostatic characteristics [110]. In these TRHs, the blood coagulation time in vitro substantially decreases, effectively shortens hemostasis time, and minimizes blood loss in vivo as demonstrated in rat models of tail amputation and hepatic hemorrhage. These hydrogels demonstrated biocompatibility by maintaining cell viability, supporting blood components, and exhibiting biodegradability while minimizing significant inflammation and promoting injury recovery. Injectable hemostatic adhesive hydrogels were generated using a two-phase mixing approach: one precursor phase comprised DHCA and CS, while the other contained β-GlyP. The mixture was incubated at 37 °C (Fig. 12) [110]. Incorporating DHCA significantly reduced the thermogelling duration in conjunction with the organic phosphate, thereby improving the viscoelasticity of the hydrogel and strengthening its internal network structure. These outcomes suggest that GlyP and DHCA effectively enhanced the temperature-dependent gelation of CS-*β*-GlyP-DHCA (CGD) formulation [110].

#### 2.3.2 Dual responsive hydrogels

DRHs are engineered to react to two distinct stimuli, offering enhanced functionality and control in intricate settings. By selectively reacting to multiple triggers, DRHs can more effectively replicate the dynamic conditions of the human body, enabling more accurate and customized therapeutic interventions. The choice of stimuli, such as pH, temperature, and light, or other combinations, is decided based on the intended applications. These hydrogels offer enhanced versatility and manipulation of single-responsive systems by enabling precise adjustments of their responses depending on the simultaneous or sequential detection of two stimuli. DRHs have the potential for advanced applications, including DDSs that require a two-step mechanism for drug release, ensuring more targeted delivery to specific tissues or disease areas.

**Fig. 13:**
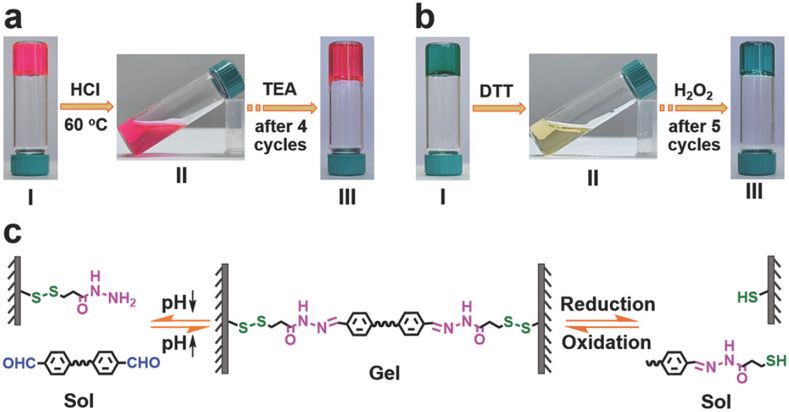
(a) pH-responsive sol-gel transition of the prepared gel. (I) The staining of hydrogel with rhodamine B, (II) hydrogel breakdown upon incorporating in the HCl solution, and (III) regenerated hydrogel after 4 cycles of gel-sol transition. (b) The redox-responsive sol-gel transition of the prepared gel. (I) The staining of hydrogel with methylene blue (MB), (II) hydrogel breakdown after addition of 1,4-dithio-dl-threitol (DTT), and (III) regenerated hydrogel after 5 cycles of gel-sol transition. (c) Representation of pH/redox dual-responsive sol-gel transitions [114]. Copyright 2017. Reproduced with permission from WILEY-VCH.

DRHs are composite materials responsive to both ambient temperature and specific pH levels. For example, a hydrogel can be designed to maintain its liquid form at ambient temperature but undergo solidification at body temperature, expanding or contracting in response to the pH of its surroundings. In DDSs, these DRHs are highly advantageous since they can be injected in liquid form, undergo gelation at the intended site, and thereafter release their payload only upon reaching a predefined pH threshold, such as the acidic conditions of a tumor. This combination guarantees a highly regulated and targeted therapeutic release [[111], [112], [113]]. In another study, Yang et al. synthesized a novel self-healing, cellulose-based hydrogel using dynamic covalent acyl hydrazone connections [114]. The hydrogels exhibited pH/redox dual-responsive sol-gel transition behaviors and were effectively utilized for the controlled release of doxorubicin. The hydrogels' reversibly cross-linked networks and superior biocompatibility made them suitable as 3D culture scaffolds for L929 cells, showing high cell viability and proliferative potential. Moreover, these self-healing hydrogels demonstrated promising uses in 3D cell culture and drug administration for TE (Fig. 13) [114].

**Fig. 14:**
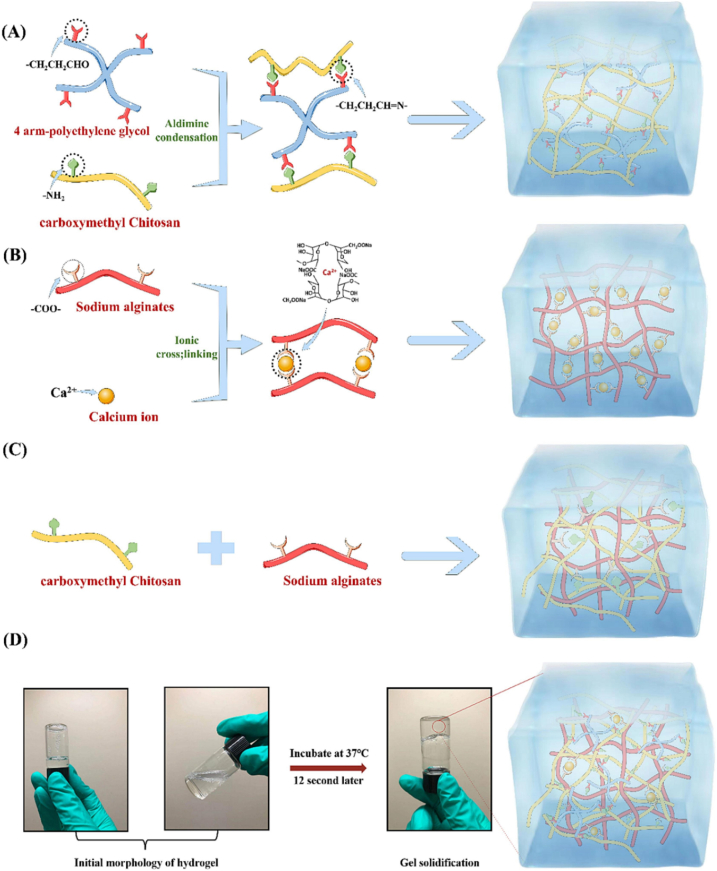
Synthesis of the CMCS/PEG + SA/CaCl2 (CPSC) hydrogel. (A) Crosslinking process of CMCS with 4-arm-PEG-pALD, (B) Crosslinking interaction between SA and CaCl_2_, (C) Electrostatic interaction between CMCS and SA, and (D) Formation mechanism of the CPSC hydrogel [115]. Copyright 2024. Reproduced with permission from Elsevier.

Wang et al. developed an injectable dual-crosslinked (DC) hydrogel scaffold composed of CMCS, 4-arm-PEG-pALD, calcium chloride (CaCl_2_), and SA with tunable mechanical properties and good biocompatibility [115]. The hydrogel combines chemical and physical crosslinking to adjust softness and hardness, promoting its use in bone repair applications. The scaffold was synthesized via in situ gelation in a physiological environment (37 °C) without using external catalysts. The chemical network formed rapidly (within 5 s) due to imine bonds between CMCS and PEG, while SA and Ca^2+^ contributed to a physical "egg-box" network. By integrating these systems, the study resolved common issues with purely chemical or physical hydrogels, such as needle blockage during injection and poor mechanical strength. Under optimized conditions, the hydrogel scaffold demonstrated favorable nutrient transport, cell infiltration, and osteogenic differentiation of rat bone MSCs. In vivo studies confirmed that the rat bone MSC-loaded hydrogels effectively supported bone reconstruction. Additionally, the mechanical properties and gelation time were adjustable by modifying the component ratio, ensuring suitability for bone defect repair. This research highlights the potential of injectable DC hydrogels with controlled properties for promoting osteogenesis and addressing bone defect challenges (Fig. 14) [115]. In another work, a novel thermosensitive and pH-responsive, self-healing, injectable hydrogel (FCAB) was developed by Tang et al. [116]. Composed of CS oligosaccharide (COS) grafted with boric acid (BA), aldehyde hyaluronic acid (A-HA) oxidized by NaIO_4_, and Pluronic F127 (F127-COS), this hydrogel was infused with deferoxamine (DFO) to facilitate the localized and precise drug release, promoting angiogenesis to treat diabetic foot ulcers. The FCAB hydrogel demonstrated exceptional therapeutic efficacy, biocompatibility, and physicochemical characteristics. In vitro tests showed that the FCAB hydrogel comprising DFO (FCAB/D) substantially enhanced the angiogenesis and migration of human umbilical vascular endothelial cells (HUVECs). Further testing in a diabetic rat foot ulcer model revealed that the FCAB/D hydrogel effectively fostered tissue regeneration and wound healing. This multifunctional hydrogel enhances diabetic wound healing by facilitating angiogenesis, epidermal regeneration, and targeted DFO release. These findings highlight the potential of FCAB/D hydrogel as an advanced biomaterial for targeted therapy in TE and regenerative medicine [116].

#### 2.3.3 Multi-responsive hydrogels

MRHs are a complex and versatile class of intelligent hydrogels capable of responding to three or more types of stimuli, including temperature, light, pH, enzymes, or electric fields. These hydrogels demonstrate exceptional customizability and complexity, particularly in sophisticated biomedical applications. MRHs are specifically designed to adapt to the complex and diverse conditions encountered in biological environments, where multiple signals may occur simultaneously. By integrating responses to various stimuli, MRHs offer improved precision in controlling drug release, scaffold disintegration, and tissue regeneration. One of the inherent challenges of MRHs lies in their intricate nature, which encompasses both design and synthesis complexities, as well as their performance within living organisms. Systematically managing several reactions requires a careful and detailed examination of the interactions among various stimuli. However, the potential advantages of these sophisticated treatments position them as a significant area of ongoing investigation [[114], [115], [116], [117]].

**Fig. 15:**
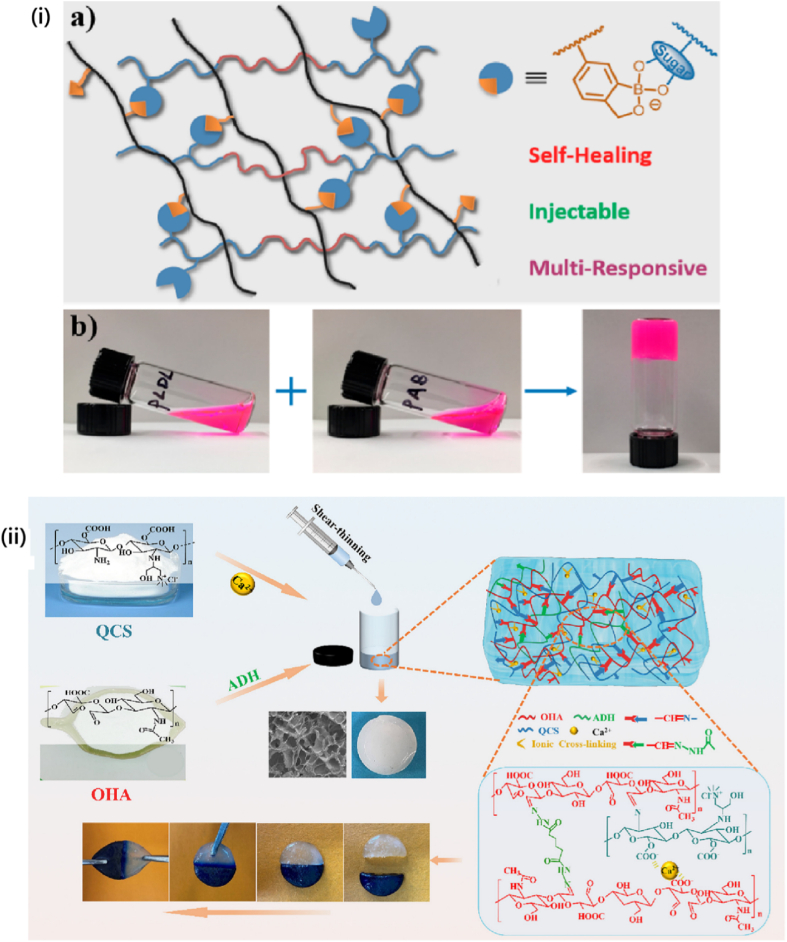
(I) A multi-responsive, injectable, and self-healing hydrogel crosslinked via the complexation of benzoxaborole and galactose (a), and hydrogel formation is achieved by combining PAB and PLDL solutions at pH 7.4 (b) [121]. Copyright 2019. Reproduced with permission from the American Chemical Society. (ii) Schematic representation of the injectable multifunctional hydrogel [122]. Copyright 2023. Reproduced with permission from Elsevier.

One instance of an MRH exhibits a response to a combination of temperature, pH, and light parameters. In this configuration, when exposed to body heat, the hydrogel undergoes a phase change from liquid to gel. However, the drug content will only be released in reaction to the local pH and a light stimulus. For example, a hydrogel employed in cancer treatment could be engineered to solidify at the temperature of the human body when administered close to a tumor, where the pH is lower compared to the adjacent healthy tissue. When subjected to a particular wavelength of light, the hydrogel may release its drug content in a highly controlled manner. This multi-responsive system ensures precise drug release on demand, both spatially and temporally. MRHs are also under investigation in TE, where they can react to various mechanical stresses, fluctuations in pH, and enzyme activities to facilitate tissue growth, restoration, and regeneration. Their versatility and adaptability make them highly promising for regenerative medicine, given the constantly shifting environmental conditions during tissue repair and regeneration [[117], [118], [119], [120]]. In a recent study, Chen et al. developed a novel ABA-type triblock copolymer (PLDL) hydrogel exhibiting LCST and galactose pendant groups [121]. This hydrogel was synthesized by combining PLDL solutions with a hydrophilic copolymer containing benzoxazole groups through dynamic benzoxazole−galactose complexation. The resulting hydrogels demonstrated a tri-fold response to sugar, temperature, and pH. Rheological studies revealed that the mechanical characteristics can be adjusted by altering the pH and the galactose/benzoxazole molar ratio. Additionally, the hydrogel demonstrated remarkable injectable and self-healing properties due to the inherent reversible nature of aryl boronic ester bonding. As a result, multi-responsive, self-healing, and injectable hydrogel offers significant potential for diverse biomedical applications (Fig. 15i) [121]. In another study, novel hydrogels were developed by combining adipic acid dihydrazide (ADH), quaternized CMCS (QCMCS), aldehyde-modified hyaluronic acid (OHA), and calcium chloride (CaCl_2_). They were synthesized through a simple one-step process, using coordination bonds and dynamic Schiff base (imine and acyl hydrazone bonds) to create a multifunctional network. The hydrogels demonstrated excellent injectability, pH responsiveness (pH 4, 7.4, and 10), self-healing (up to 94 %), and notable compressive strength (up to 896.30 kPa). The reversible interactions between QCS and OHA facilitated a dynamic structure suitable for physiological conditions, with a gelation time of approximately 54 s. The hydrogels also exhibited high drug loading capacity (121.3 mg g^−1^), prolonged drug release (over 120 h), and a Fickian diffusion mechanism, as analyzed by Korsmeyer-Peppas kinetics. Loaded with acetylsalicylic acid (ASA) as a model drug, the hydrogels displayed favorable swelling, biodegradability, biocompatibility, and antibacterial properties. Overall, these injectable, multifunctional hydrogels hold considerable clinical potential for drug delivery applications due to their dynamic bonding, excellent mechanical properties, self-healing capabilities, and controlled drug release profile (Fig. 15(ii)) [122]. In multi-responsive CS-based hydrogels, interactions among stimuli are often hierarchical, rather than simply additive. Temperature-induced phase transitions alter the polymer conformation and ionization state, thereby affecting pH sensitivity and drug release kinetics. In these systems, the primary stimulus (e.g., temperature) typically drives structural reconfiguration, which in turn enables secondary responses (e.g., pH- or enzyme-mediated degradation), underscoring the importance of designing hydrogels with synergistically coordinated stimulus hierarchies [123].

#### 2.3.4 Comparative evaluation of responsive hydrogel systems

The classification of hydrogels into single-, dual-, and multi-responsive categories provides a practical framework for selecting suitable systems based on the needs of biomedical applications. Each class exhibits distinct advantages and constraints, subject to the complexity of the target environment and therapeutic objective.

**Single-responsive hydrogels** offer simplicity in design and predictable behavior, making them ideal for localized drug delivery or basic wound healing. However, they may lack adaptability to dynamically changing biological conditions.

**Dual-responsive hydrogels** improve precision by responding to two stimuli—such as pH and temperature—thus enhancing their control over drug release or gelation behavior. They remain relatively easy to synthesize but still provide more targeted responses compared to single-responsive types.

**Multi-responsive hydrogels** incorporate three or more stimuli, offering high specificity and tunability. These systems are promising for complex biomedical scenarios such as tumor microenvironments or TE. Yet, their synthesis is often challenging and may hinder scalability or clinical translation. Table 2 provides a comparative overview of single-, dual-, and multi-responsive hydrogels, highlighting their stimulus types, key advantages, limitations, and typical biomedical applications.

**Table 2:** Comparative analysis of single, dual, and multi-responsive hydrogels.

Type	Stimuli	Advantages	Limitations	Key biomedical applications	Ref.
**Single-responsive**	One (e.g., pH, temp, redox)	Simple, cost-effective, predictable response	Limited adaptability to complex biological environments	Topical drug delivery, wound healing	[[124], [125], [126]]
**Dual-responsive**	Two (e.g., pH + temp)	Improved specificity and control	Increased formulation complexity	Injectable DDS, inflammation targeting	[127]
**Multi-responsive**	≥3 (e.g., pH + temp + redox)	Highly tunable, suitable for complex conditions	Complex synthesis, regulatory barriers	Tumor therapy, TE scaffolds	[128]

## 3 Chitosan: properties and biomedical relevance

**Fig. 16:**
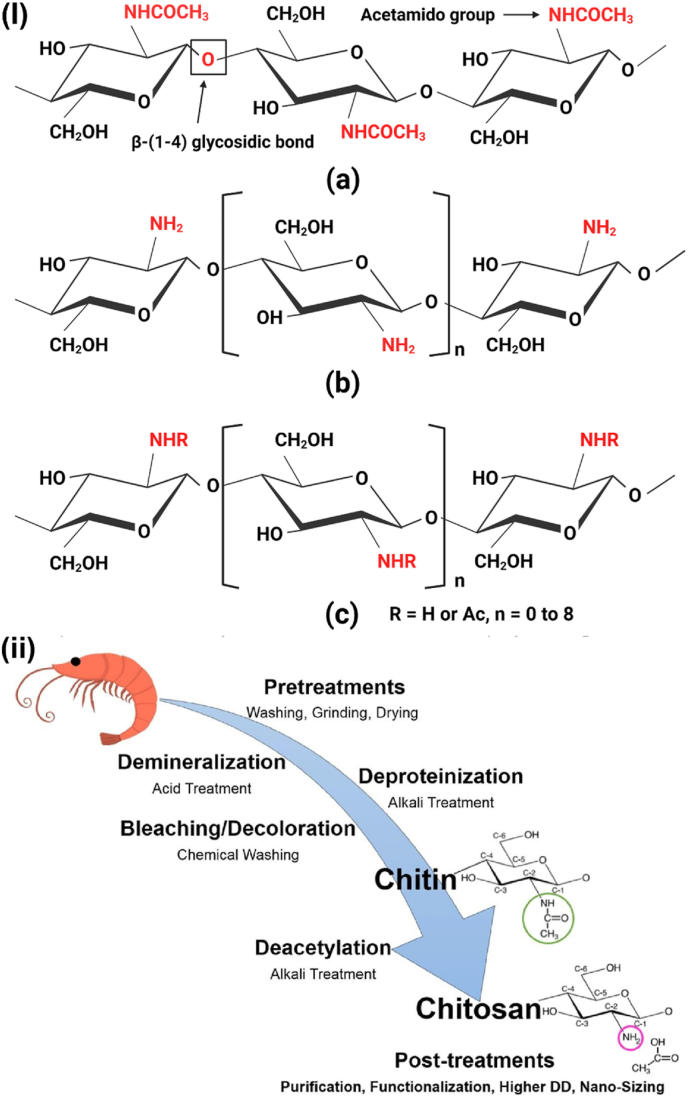
(I) Chemical structures of (a) fully acetylated chitin [poly-(1–4)-N-acetyl-D-glucosamine], (b) fully deacetylated CS [poly-(1–4)-D-glucosamine], and (c) Citooligosacharide [130]. Copyright 2022. Reproduced with permission from Elsevier. (ii) Steps involved in extracting chitin and synthesis, and post-treatment of CS [133]. Copyright 2021. Reproduced with permission from Elsevier.

Unlike previous reviews that broadly address the general properties of CS, this section specifically focuses on the structural and physicochemical characteristics of CS that are crucial for its behavior in injectable smart hydrogels. Key aspects such as sol-gel responsiveness, tight junction interactions, and enzymatic degradation under physiological conditions are thoroughly explored. CS is the second-largest naturally occurring, widely used, and most abundant biopolymer, which is synthesized via deacetylation of chitin [129]. It is a linear polysaccharide consisting of varying proportions of (3R,4R,5S,6R)-3-amino-6-(hydroxymethyl)tetrahydro-2H-pyran-2,4,5-triol (D-GlcN) and β-D-(acetylamino)-2-deoxy-glucopyranose (GlcNAc) units, linked by β-(1–4) glycosidic bonds, as illustrated in Fig. 16i [130]. Chitin, the primary source of CS, is widely extracted from fungi, algae, echinoderms, segmented worms (Annelida), mollusks, jellyfish (Cnidaria), roundworms (Aschelminthes), and various other organisms, particularly arthropods. In arthropods, chitin serves as a structural component of tendons, exoskeletons, and the linings of digestive, excretory, and respiratory tracts, as well as external insect structures and certain fungal cell walls [131]. It is also present in the iridophores (reflective cells) of the eyes and skin in cephalopods and arthropods (phylum Mollusca) and the epidermal cuticle of vertebrates. Notably, the epidermal cuticle of Paralipophrys trigloides is chitinous [132]. The transformation of chitin into CS involves four key steps: deproteinization, demineralization, decolorization, and deacetylation, as illustrated in Fig. 16(ii) [133]. When the deacetylation degree of chitin reaches approximately 50 % (which varies based on the polymer's origin), it dissolves in aqueous acidic solutions and is then referred to as CS [134]. CS contains hydroxyl and amino groups, which facilitate the generation of covalent bonds through various pathways, such as diminishing amination, esterification, and etherification reactions [135]. These functional groups are key determinants of its solubility and other physicochemical properties. CS exhibits several inherent properties, including mucoadhesion, antimicrobial activity, and permeability enhancement [136]. At low pH, the protonation of the amino groups imparts a positively charged (cationic) character to the CS molecule, enabling electrostatic interactions with negatively charged mucosal surfaces and promoting mucoadhesion. This process is also influenced by hydrophobic interactions and hydrogen bonding [137]. CS interacts with the anionic regions of cellular membranes, modulating tight junction proteins and enhancing paracellular transport. The most critical parameters affecting the behaviour of CS are its molecular weight and deacetylation degree [138]. The acetylation degree influences its polycationic nature in acidic environments, while adjustments to molecular weight can tailor its viscosity, water solubility, and overall functionality [139].

Biologically, CS is a highly active molecule, exhibiting various biological activities, such as antimicrobial, anti-inflammatory, antifungal, antihyperglycemic, antitumor, anticancer, antidiabetic, antioxidant, and wound-healing properties [54,93,132,[140], [141], [142], [143], [144]]. These diverse activities make CS widely utilized in biomedical applications and DDSs, including nasal, oral, ocular, pulmonary, gene, mucosal, and vaccine delivery [145]. Despite its versatility, CS has certain drawbacks: its low solubility in water and specific organic solvents, which may restrict its use in several areas [146]. To address this, CS is frequently subjected to chemical modification targeting its –NH_2_ and –OH groups. This modification enhances its chemical and physical properties while retaining its essential bioactivity, expanding its applicability. Derivatives, such as thiolated CS, trimethyl CS, glycol CS, and carboxymethyl chitosan (CMCS), are widely employed as drug carriers by virtue of their superior biocompatibility, bioactivity, and biodegradability [147].

### 3.1 Physicochemical properties of chitosan

The physicochemical features, such as solubility, molecular weight, degree of deacetylation, and viscosity of CS, define the relationship between its chemical structure and its versatile applications across various scientific and industrial domains [148,149]. Among these, the degree of deacetylation and average molar mass are the most commonly evaluated properties, irrespective of the CS's intended use. Additional parameters commonly evaluated include solubility, viscosity, crystallinity, water content, nitrogen content, ash content, and water retention capacity. Furthermore, heavy metals, residual proteins, and endotoxin concentrations are critical factors in determining CS's suitability as a biomaterial [150].

#### 3.1.1 Solubility

CS dissolves in acidic solvents but remains insoluble in neutral and alkaline conditions. While native chitin is typically insoluble, deacetylation yields CS with primary amino groups (pKa of 6.5), conferring solubility in acidic media. In such environments, the protonation of amino groups imparts a positive charge, facilitating dissolution. However, above pH 6, the loss of protonation leads to a decrease in solubility [148]. Apart from pH, factors such as molecular weight, deacetylation degree, polymer crystallinity, and temperature significantly influence the solubility of CS [149]. This solubility behavior is critical in CS-SIH formulations, as it allows for injectable delivery under acidic conditions and enables gelation when exposed to physiological pH, supporting pH-responsive systems for targeted drug release.

#### 3.1.2 Molecular weight

The molecular weight of CS, which typically ranges from 50 kDa to 2000 kDa, significantly influences its physicochemical and biological properties. It is primarily influenced by the source material and the extraction process [150]. CS can be available in low, medium, or high molecular weights. Higher molecular weights are associated with increased viscosity and reduced solubility, which is often undesirable in industrial processes. In contrast, low-molecular-weight CS exhibits improved solubility and stability, making it a preferable choice for various biological and industrial applications [151]. The molecular weight of CS directly affects the viscosity of precursor solutions and the mechanical strength of the resulting hydrogel network, both of which are crucial for tuning injectability and structural integrity post-gelation in responsive systems.

#### 3.1.3 Degree of deacetylation

The deacetylation degree is instrumental in defining the physicochemical properties and applications of CS. It indicates how the amino groups are distributed along the polymer chain. CS exhibits a cationic nature in acidic media due to the amino groups in the polymer chain. As a result, the degree of deacetylation significantly affects its solubility and viscosity [152]. The deacetylation degree (DD) is defined as the molar fraction of glucosamine (GlcN) units relative to the total monomeric units and is calculated as:

(1) $$DD=\frac{\text{nGlcN}}{\text{nGlcN}+\text{nGlcNAcNAc}}$$

where nGlcN is the average number of GlcN units, and nGlcNAc is the average number of N-acetylglucosamine units. A DD exceeding 50–60 % typically signifies the successful conversion of chitin to CS [153]. A higher DD enhances the density of primary amine groups, improving ionic crosslinking potential and pH sensitivity—two core mechanisms driving CS-SIH smart behavior and in situ gelation.

#### 3.1.4 Viscosity

Viscosity is a critical parameter for evaluating the processability and functionality of CS, particularly in industrial applications. It is highly influenced by molecular weight, deacetylation degree, particle size, and storage duration, and can vary significantly depending on the type of CS and its preparation method [154]. For instance, at a 1 % (w/v) concentration in acetic acid, high molecular weight CS with a high deacetylation degree may show viscosities ranging from 100 to 1000 mPa s. Conversely, low molecular weight CS under similar conditions may display viscosities as low as 10–50 mPa s. Viscosity tends to increase with higher degrees of deacetylation and lower molecular weights. Nano CS solutions typically have around 30 % lower viscosity than their conventional counterparts at equivalent concentrations. Moreover, the storage period can impact viscosity; after 24 h of storage, typical CS solutions experience a 10 % reduction, while nano CS colloids show a 17 % drop. Viscosity is a key indicator of polymer stability in solution, as a decline over time may indicate molecular degradation or structural alterations during storage [152]. The intrinsic viscosity of CS solutions influences shear-thinning behavior and syringeability. Optimized viscosity is essential to ensure smooth injection while preserving responsive gelation kinetics in vivo.

### 3.2 Functional bioactive properties of chitosan relevant to SIHs

#### 3.2.1 Biocompatibility and biodegradability

CS exhibits superior biocompatibility and biodegradability, positioning it as an ideal candidate for various biomedical applications [155]. Both in vitro and in vivo studies provide robust quantitative evidence supporting these characteristics. Enzymatic degradation, primarily facilitated by lysozyme, typically results in a mass loss of 30–60 % within 7–14 days, depending on the molecular weight and degree of deacetylation. Higher deacetylation levels lead to prolonged degradation due to increased crystallinity, while lower molecular weight CS exhibits a more rapid degradation profile [156].

Cytotoxicity evaluations demonstrate that CS-based hydrogels consistently maintain over 85 % cell viability across a range of human and animal cell lines, including fibroblasts, keratinocytes, and mesenchymal stem cells (MSCs), as assessed using MTT and Live/Dead assays. In vivo implantation studies, including subcutaneous and skin wound models in rats, have shown minimal inflammatory responses, successful tissue integration, and complete biodegradation within 2–4 weeks, thereby confirming excellent tissue compatibility. These findings underscore the material's clinical potential for applications that require safe resorption and minimal immunogenicity [157].

#### 3.2.2 Mucoadhesion

**Fig. 17:**
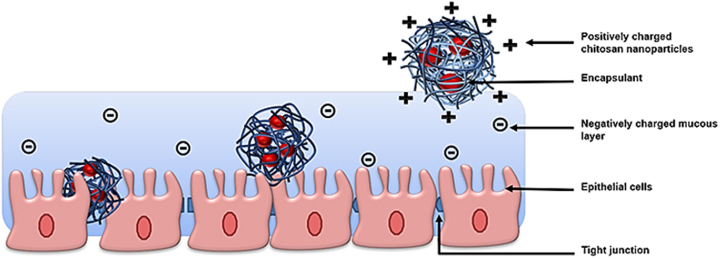
CS-loaded nanoparticle interaction with the mucus layer [165]. Copyright 2022. Adapted from DovPress.

Synthetic and natural polymers exhibit mucoadhesive properties, which are influenced by their chemical structure and reactive group concentrations. Mucoadhesive polymers are typically composed of reactive groups such as -COOH, -SO_3_H, -NH_2_, and -OH, which make non-covalent bonds with mucin, thereby adhering to the mucosal surfaces—a process referred to as mucoadhesion [158,159]. CS's mucoadhesive behavior is primarily attributed to its cationic nature. The mucus gel layer contains anionic components, including sialic acid and sulfonic acid, which interact ionically with the CS's cationic groups, resulting in mucoadhesion [160]. The mucoadhesive capacity of CS is enhanced with increasing deacetylation degree and molecular weight, but decreases with higher cross-linking. This characteristic enables CS to adhere to the upper gastrointestinal and respiratory tracts, facilitating sustained drug release [157,161]. The trimethylation significantly increases CS's cationic charge, thereby enhancing its mucoadhesion. When methyl groups are added to their amino groups, trimethyl CS becomes soluble in neutral and basic pH environments [146]. Similarly, CMCS, including carboxyl and amino groups, demonstrates mucoadhesion across both basic and acidic pH ranges [162]. Moreover, strong interactions between the cysteine residues of mucus glycoproteins and the thiol groups in CS further enhance its mucoadhesive properties [163,164]. The CS-mucus interaction is schematically illustrated in Fig. 17 [165].

#### 3.2.3 Permeation enhancement

**Fig. 18:**
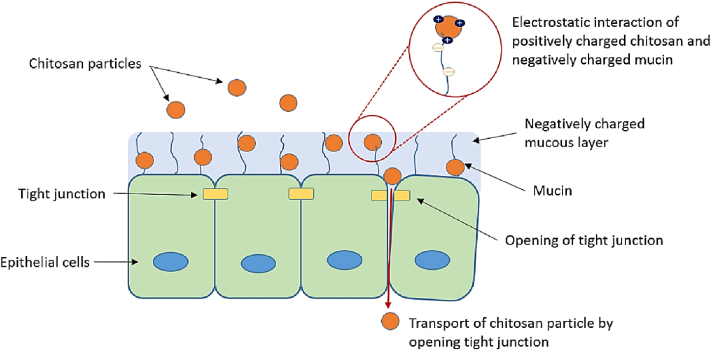
Opening of CS-mediated reversible tight junction [136]. Copyright 2023. Reproduced with permission from Elsevier.

With its cationic properties, CS interacts with the mucus membrane to help open tight junctions between cells. This action enhances the movement of substances through mucosal cells, improving drug permeation [166]. With a high molecular weight and deacetylation degree, CS has significantly increased epithelial permeability [167,168]. Modified CS derivatives exhibit even more significant permeation enhancement compared to unmodified CS [169]. Among the quaternized derivatives of CS, four types have demonstrated an ability to increase transepithelial electrical resistance, indicating their potential to open tight junctions between cells [[170], [171], [172]]. Transepithelial electrical resistance increases in the following order: CS, followed by triethyl CS, dimethyl ethyl CS, and finally, trimethyl CS [173,174]. Trimethylation of CS's primary amino group results in a more pronounced effect than unmodified CS. Furthermore, the thiolation of CS enhances its permeation properties through mucosal membranes [175,176]. The mechanism by which CS enhances permeation through the tight junction opening is depicted in Fig. 18 [136].

#### 3.2.4 In situ gelling behaviour

**Fig. 19:**
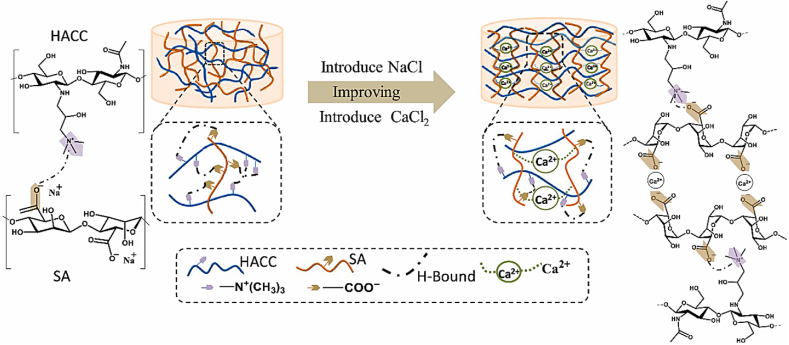
Schematic illustration of hydrogel formation and enhancement [131]. Copyright 2021. Reproduced with permission from Elsevier.

CS-based gelling systems can be divided into in-situ cross-linking and phase separation systems. In chemical cross-linking, bonds are created between CS chains, leading to a gel matrix. The in-situ phase separation technique produces in-situ gelling systems by adjusting the polymer solubility, typically initiated by variations in pH or temperature. This physical cross-linking method is used to form CS hydrogels [177]. Geng et al. developed a dual-cross-linked hydrogel by mixing CS, sodium alginate (SA), and quaternary ammonium salt. The hydrogel was formed in situ via electrostatic interaction energy between polyanions and polycations, along with hydrogen bonding and ionic crosslinking, as depicted in Fig. 19 [131]. The resulting hydrogel featured a uniform 3D network structure and demonstrated satisfactory mechanical properties. Additionally, it exhibited excellent injectability, with a compression strength of 27.65 kPa. Its biocompatibility was confirmed through cytotoxicity tests, showing that the hydrogel was non-toxic to NIH-3T3 cells [131].

#### 3.2.5 Controlled drug release

**Fig. 20:**
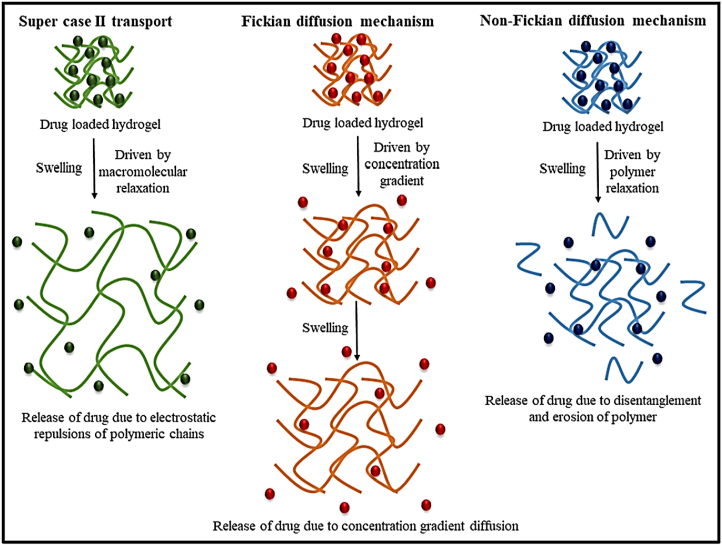
Different drug release mechanisms of hydrogels [180]. Copyright 2024. Reproduced with permission from John Wiley & Sons.

Controlled drug release is particularly advantageous for drugs exhibiting suboptimal plasma levels or those administered orally [178]. When conventional mechanisms, such as drug dissolution, diffusion, degradation, osmotic systems, or membrane control, fail to ensure sustained drug release, the desired effect can be achieved through ionic interactions. Anionic polymeric excipients, including alginate, sodium carboxymethyl cellulose (SCMC), or polyacrylates, are typically employed to control cationic drug release. However, CS is often preferred when working with anionic drugs. For instance, it has been successfully used as a drug carrier matrix for the sustained release of the anionic drug naproxen [179]. CS's ability to form ionic cross-links results in the formation of stable complexes, which facilitate prolonged and controlled drug release. When integrated with anionic polymers such as carrageenan, alginate, pectin, HA, and polyacrylates, CS forms stable complexes that enable sustained drug release [142,162,180,181]. Fig. 20 demonstrates the numerous mechanisms for drug release from hydrogels [180].

#### 3.2.6 Antimicrobial agent

CS and its derivatives show antimicrobial activity against various microorganisms, including fungi, bacteria, and yeast and several hypotheses have been proposed to understand the precise mechanism of their antibacterial actions [157,182,183]. One mechanism suggests that CS interacts with the anionic components of the cell membrane, increasing cellular permeability and ultimately leading to cell death. Another proposed mechanism involves CS penetrating the cell membrane, binding to bacterial DNA, and interacting with surface molecules, preventing mRNA and causing cell death. Additionally, CS's ability to chelate metal ions can inhibit microbial growth. Due to differences in their cell structures, CS has demonstrated effectiveness against different Gram-positive bacteria, Gram-negative bacteria, and fungi. CS also exhibits antifungal properties against different fungi by hindering spore germination and mycelia growth [157,184]. Recent research has indicated that CS's antimicrobial activity is affected by several factors, including deacetylation degree, concentration, molecular weight, viscosity, and the pH of the reaction mixture. Additionally, its effectiveness is closely related to the specific type of target microorganisms. Remarkably, CS demonstrates effectiveness at lower doses with reduced toxicity toward mammalian cells compared to other compounds. Overall, the diverse antimicrobial activity of CS makes it a promising alternative to antibiotics in addressing antibiotic resistance in microorganisms [151]. An overview of the key characteristics, biological activities, chemical modifications, and biomedical applications of CS matrix relevant to CS-SIHs is summarized in Table 3.

**Table 3:** Key characteristics and biomedical Relevance of CS polymer matrix.

Key Characteristics	Brief Description	Ref.
**Source**	Derived from chitin via alkaline deacetylation	[185]
**Key Properties**	Biodegradable, biocompatible, mucoadhesive, cationic, non-toxic	[151]
**Solubility**	Soluble in dilute acidic solutions (pH < 6.5)	[186]
**Functional Groups**	Primary amine (-NH_2_), hydroxyl (-OH), acetyl groups	[187]
**Biological Activities**	Antibacterial, antifungal, antioxidant, hemostatic, immunomodulatory	[188]
**Biomedical Functions**	Hydrogel backbone, injectable matrix, controlled drug delivery, wound healing	[11]
**Common Modifications**	Carboxymethylation, quaternization, thiolation, sulfation, PEGylation	[189]
**Representative Derivatives**	CMCS, TMC, thiolated CS, N-alkylated CS	[190]
**Reactive Potential**	Form ionic gels, Schiff bases, or crosslinked networks via multiple chemistries	[191]
**Applications in CS-SIHs**	Drug delivery, gene therapy, regenerative scaffolds, antimicrobial coatings	[192]

## 4 Design and synthesis of CS-SIHs

### 4.1 Design considerations of CS-SIHs

The design of CS-SIHs necessitates careful consideration of several key factors to ensure their efficacy in TE applications. One fundamental aspect is the mechanical characteristics of the hydrogel. The scaffold should possess sufficient mechanical robustness to support the developing tissue and withstand physiological stresses without the risk of collapse or distortion. Key parameters, such as elasticity, tensile strength, and compressive strength, must be finely tuned according to the targeted specific tissue. Another critical factor is the degradation rate of the hydrogel. The degradation rate of the scaffold should be calibrated to correspond with the tissue's growth rate. A rapid hydrogel degradation may deprive the tissue of essential nutrients for growth [193]. Conversely, a sluggish degradation could hinder tissue growth or lead to other complications. Control over the deterioration rate requires adjusting the hydrogel's composition and crosslinking density. Additionally, the cellular interaction with the hydrogel is a key determinant in the design process. The hydrogel should ideally facilitate cell adhesion, differentiation, and proliferation. This involves optimizing surface characteristics, including roughness, hydrophilicity, and the possible incorporation of bioactive compounds. The efficacy of the hydrogel as a scaffold in TE is directly influenced by its ability to support these critical biological processes [194].

### 4.2 Synthesis methods of CS-SIHs

CS-SIHs can be synthesized utilizing different techniques, each influencing the characteristics of the final product.

#### 4.2.1 Chemical crosslinking

**Fig. 21:**
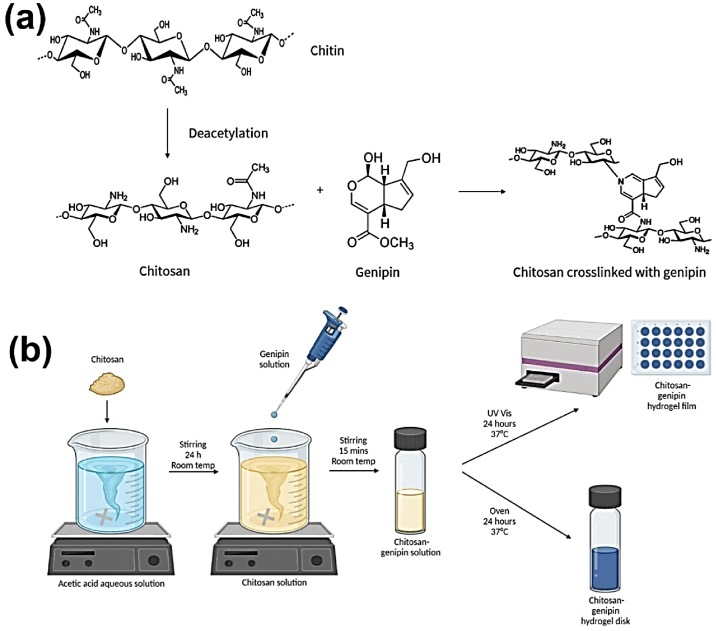
(A) Chemical transformation of chitin into CS through deacetylation, followed by the crosslinking reaction between CS and GP, and (b) synthesis of CS-GP hydrogel disks [199]. Copyright 2022. Adapted from the Royal Society of Chemistry.

Chemical crosslinking is extensively employed to produce CS-hydrogels. This method forms covalent bonds between CS chains, making a robust 3D network. This technique typically involves crosslinking agents interacting with the functional groups in CS, including amino and hydroxyl groups, to form a durable hydrogel structure. GP and glutaraldehyde are commonly used crosslinkers to form strong covalent bonds, enhancing the hydrogel's strength, stability, and flexibility for interaction with living organisms. Unlike synthetic compounds, GP is an endogenous crosslinker that binds to amine groups in CS, creating a highly biocompatible hydrogel that is non-toxic to living organisms and cells. This crosslinking technique has various biomedical applications in TE, including wound dressings, where CS hydrogels crosslinked with GP provide a moist environment that supports wound healing while maintaining the long-term structural integrity of the cells. Additionally, this method enables precise control over the mechanical strength and degradation rate of hydrogels [[195], [196], [197], [198]]. Fig. 21 (a) shows the chemical transformation of chitin to CS through the deacetylation process, as well as the chemical crosslinking reaction between CS and GP, while Fig. 21 (b) depicts the synthesis process of CS-GP hydrogel disks [199].

**Fig. 22:**
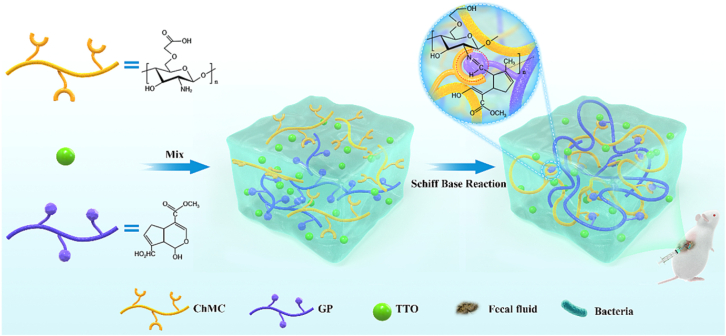
Reaction mechanisms of CMCS/GP composite hydrogel [200]. Copyright 2023. Reproduced with permission from Elsevier.

Injectable hydrogels are of significant interest for wound healing due to their ability to serve as carriers of bioactive molecules and promote tissue repair with minimal invasiveness. Nevertheless, integrating lipid-soluble substances into a hydrogel network presents challenges due to differences in polarity. Recently, Hu et al. encapsulated tea tree oil (TTO) within a hydrogel using an emulsification technique to synthesize a robust, injectable, antibacterial hydrogel through a Schiff base reaction between GP and CMCS (Fig. 22) [200]. CMCS is an emulsifier and a gel-forming agent that produces a heterogeneous hydrogel. The resulting hydrogel exhibits excellent biocompatibility, antibacterial efficacy (over 90 %), and high adhesive strength (about 162.75 kPa). Furthermore, an anal fistula-like wound healing study showed that this heterogeneous hydrogel exhibits effective slow-release properties for TTO, accelerating the healing process. This hydrogel holds substantial promise for treating complex anal fistula wounds [200].

#### 4.2.2 Physical crosslinking

**Fig. 23:**
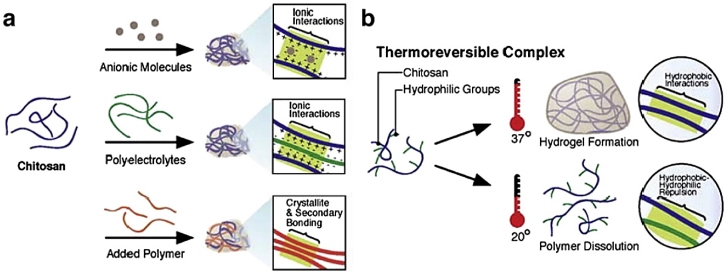
CS-based hydrogel networks formed through various physical interactions: (a) CS networks created with ionic molecules, neutral polymers, and polyelectrolyte polymers, and (b) thermo-reversible networks of CS graft copolymers that transition into a semi-solid gel at body temperature and remain liquid below RT [193]. Copyright 2010. Reproduced with permission from Elsevier.

Physical crosslinking is a non-covalent technique for constructing hydrogels based on CS particles. Physically crosslinked CS hydrogels exhibit distinct structural, physicochemical, and mechanical characteristics, which can be easily modified by varying the concentration and type of CS and crosslinking agents [193,201]. The process involves ionic bonding, hydrogen bonding, or environmental fluctuations (e.g., pH and temperature) to form a 3D network (Fig. 23) [193]. Non-chemical crosslinks simplify and maximize the environmental benefit of the process by eliminating the need for external crosslinking chemicals, thereby reducing cytotoxicity. This is particularly advantageous for applications that require reduced toxicity. A typical physical crosslinking technique is thermally induced gelation, where cooling the solution allows the formation of CS hydrogels through hydrogen bond creation, resulting in a gel matrix. A well-studied example is polyol-phosphate systems, where cooling-induced interaction between CS and phosphate ions forms hydrogels. These hydrogels are suitable for biomedical applications, including drug delivery and wound healing, as they offer compatibility and customizable mechanical properties that match those of natural biological systems. An example is the development of CS-based thermosensitive hydrogels for targeted DDSs, which solidify upon subcutaneous injection while maintaining their liquid state at ambient temperature [194,202].

**Fig. 24:**
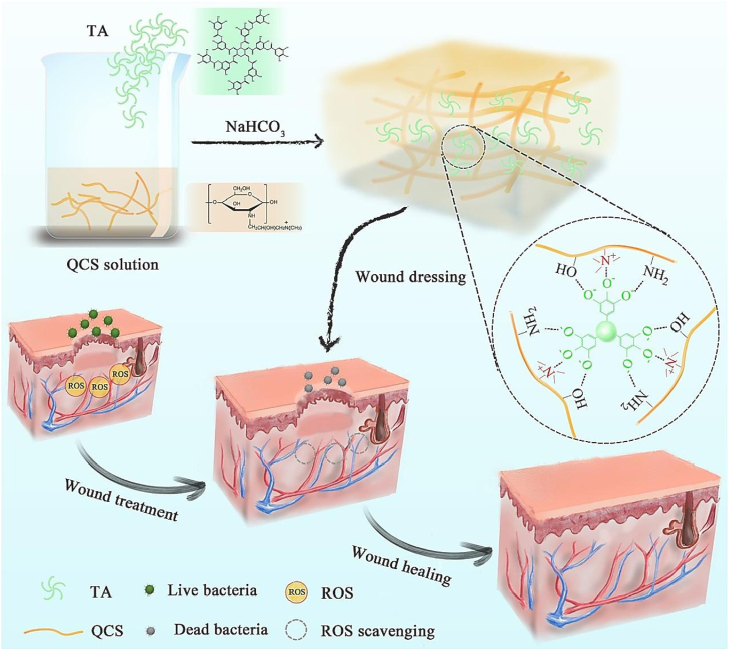
Fabrication of QCS/TA hydrogel and its physical cross-linking mechanism for diabetic wound healing [203]. Copyright 2022. Reproduced with permission from Elsevier.

In another study, Pan et al. made a hydrogel dressing with superior ROS scavenging capabilities and exceptional antibacterial efficacy by incorporating TA into the quaternized CS (QCS) matrix [203]. The appropriate physical crosslinking between QCS and TA conferred self-healing and injectable capabilities to the QCS/TA hydrogel, making it withstand external pressure from irregularly shaped wound dressings. The sodium bicarbonate (NaHCO_3_) neutralization approach, aided by hydrochloric acid (HCl) dissolution, allows the conversion of QCS and TA into an injectable hydrogel via a simple one-step process (Fig. 24) [203]. The findings indicated that this hydrogel might enhance coagulation, inhibit inflammation, and accelerate collagen deposition in a diabetic rat skin defect model. This investigation offers an efficient and convenient approach for fabricating injectable hydrogel dressings with potential therapeutic applications in diabetic wound treatment [203].

**Fig. 25:**
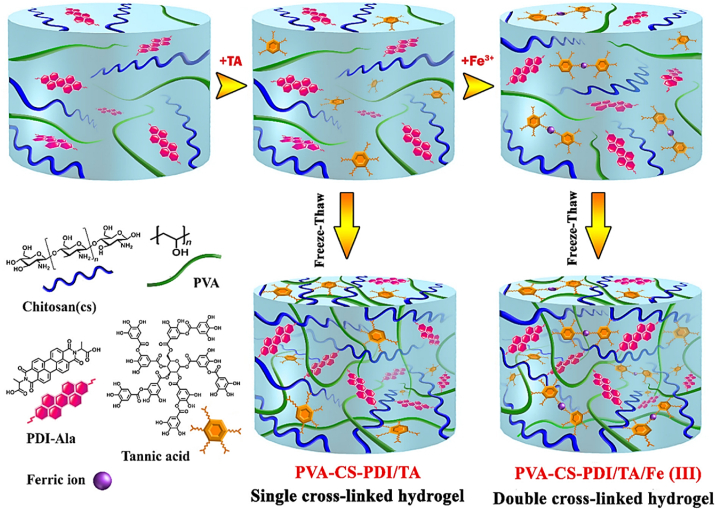
Formation of single and double cross-linked photosensitizing supramolecular hydrogels [204]. Copyright 2022. Reproduced with permission from Elsevier.

**Fig. 26:**
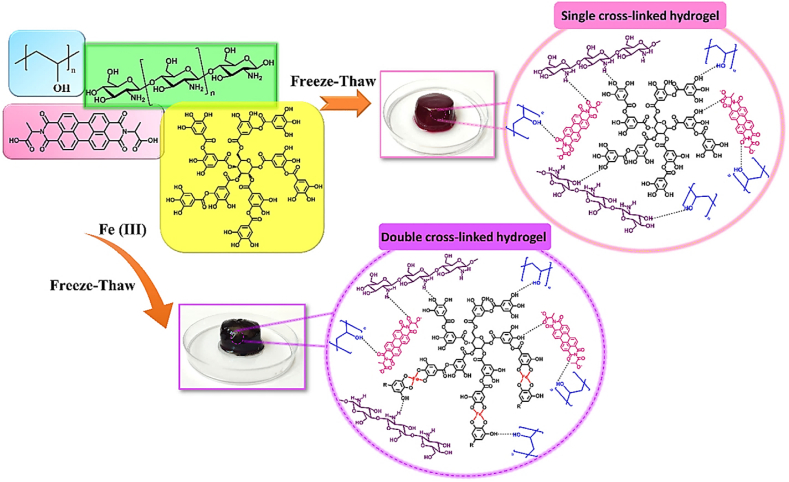
Fabrication of the PVA-CS-PDI/TA and PVA-CS-PDI/TA/Fe (III) hydrogels, along with the potential interactions, including hydrogen bonding and metal-catechol coordination bonds [204]. Copyright 2022. Reproduced with permission from Elsevier.

Azadikhah et al. introduced an economical and straightforward approach to producing photosensitizing supramolecular hydrogels with remarkable multifunctional characteristics via a freeze-thawing technique. Two types of hydrogels were synthesized: single cross-linked hydrogels (PVA-CS-PDI/TA) and double cross-linked hydrogels (PVA-CS-PDI/TA/Fe (III)), using metal coordination with Fe (III) and intermolecular hydrogen bonding. Fig. 25 schematically presents the formation of single and double-cross-linked photosensitizing supramolecular hydrogels [204]. The double cross-linked hydrogels exhibited enhanced mechanical properties relative to their single cross-linked counterparts, mainly due to the inclusion of Fe (III). These hydrogels demonstrated numerous advantages, including injectability, shear-thinning characteristics, and rapid self-healing capabilities, enhancing their applicability in biomedical fields. Their photodynamic efficiency was considerably improved in the double-cross-linked hydrogels due to increased singlet oxygen production facilitated by Fe (III). Moreover, both hydrogel variants exhibited cytocompatibility, low hemolytic activity, and antioxidant and antibacterial properties. These hydrogels are potential candidates for wound dressing and PDT in cancer treatment. Their rapid self-repair capability, mechanical durability, and biological activity enhance their utility in therapeutic environments. Biocompatible, cost-effective chemicals and their multifunctionality highlight the potential of hydrogels as an adaptable approach for managing wounds and cancer via PDT. This research underscores the novel incorporation of non-covalent interactions to develop high-performance photosensitizing hydrogels, offering a biocompatible and viable alternative for advanced biological applications. The PVA-CS-PDI/TA and PVA-CS-PDI/TA/Fe (III) hydrogels were prepared using a freeze-thawing method to create physically cross-linked structures as depicted in Fig. 26 [204]. In the PVA-CS-PDI/TA hydrogels, hydrogen bonding occurred between TA's phenolic hydroxyl and PVA's alcohol hydroxyl, as well as between TA and the hydroxyl and amine groups of CS, resulting in stronger cross-linking. The photosensitizer PDI-Ala interacted with the polymer chains and TA via hydrogen bonds. A concentration of 1.5 wt% PDI-Ala was selected to maintain solution homogeneity. For the PVA-CS-PDI/TA/Fe (III) hydrogels, a second cross-linking occurred through coordination between Fe (III) and the TA phenolic hydroxyl group, as confirmed by the color change to dark purple, indicating successful catechol/Fe^3+^ complex formation [204].

#### 4.2.3 Ionic crosslinking

**Fig. 27:**
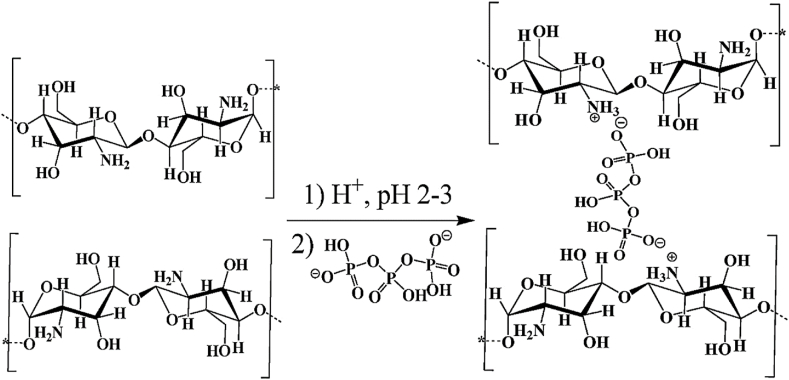
Ionic interaction of CS interaction with TPP [206]. Copyright 2019. Adapted from Elsevier.

Ionic crosslinking, a synthesis method for CS-SIHs, combines the positively charged amino groups of CS with multivalent anions to produce a physically cross-linked scaffold. This process is driven by electrostatic attractions rather than covalent bonds, making it easier to assemble and potentially reversible [203,204]. Ionic crosslinking naturally occurs in mild settings without harsh chemicals or external agents, making it highly suitable for applications where biocompatibility and non-toxicity are paramount [[205], [206], [207]]. Crosslinking agents commonly include multivalent metal ions such as calcium or zinc and tripolyphosphate (TPP). TPP is often employed as an ionic crosslinker since it is considered as a safe compound. The positively charged amino groups of CS interact with the negatively charged TPP under the influence of pH, forming an intermolecular or intramolecular network structure as shown in Fig. 27 [206]. A notable example is the controlled drug delivery using TPP-cross-linked CS hydrogels, which allow for the encapsulation and controlled release of therapeutic compounds through porous structure and adjustable degradation rate [205,208,209].

#### 4.2.4 Enzymatic crosslinking

Enzymatic crosslinking is a sustainable and environmentally advantageous technique for producing CS-SIHs, using enzymes to create covalently bonded networks. Unlike chemical crosslinking, this approach avoids potentially hazardous chemicals, making it especially suitable for biomedical applications, such as drug delivery and TE [210,211]. Transglutaminase is one of the most commonly employed enzymes in this context, facilitating covalent bond formation between the amino groups of CS and various amine-containing molecules. This enzymatic reaction generates a durable hydrogel with excellent biocompatibility and tunable degradation rates, which can be tailored to address the specific application needs [[210], [211], [212]]. Under mild reaction conditions, usually physiological pH and temperature, the bioactivity of embedded cells and therapeutic agents is maintained, making it well-suited for in situ applications. For example; transglutaminase-crosslinked carboxylated CS hydrogels were investigated for skin wound healing applications [212]. The prepared hydrogels exhibited enhanced cell adhesion, proliferation and migration of L929 cells, indicating their good biocompatibility. The study of full-thickness skin defect model suggested that the hydrogels were significantly effective in preventing the invasion of the wound by outside bacteria certifying that they are effective in fostering wound healing over pathologic healing [212]. Moreover, enzymatic crosslinking was also employed in studies related to the controlled release of growth factors, thereby expanding its potential in regenerative medicine applications [207,210,211].

#### 4.2.5 Radiation crosslinking

**Fig. 28:**
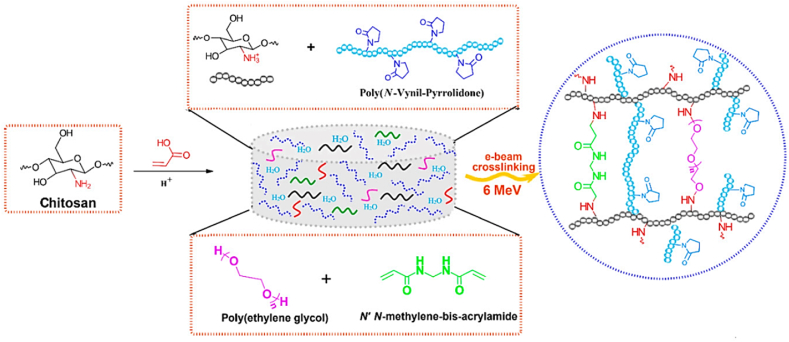
Formation of CS-PVP-PEG-PAA hydrogels through e-beam cross-linking and the cross-linked structure [217]. Copyright 2020. Adapted from MDPI.

Radiation crosslinking is a sustainable and innovative method for fabricating CS-based hydrogels using high-energy radiation, such as UV light or gamma rays. This technique induces polymer crosslinking without the use of chemical crosslinkers, ensuring superior biocompatibility—an essential feature for biomedical applications [[213], [214], [215], [216]]. This technique irradiates CS in an aqueous solution, whereby radiation produces free radicals in the polymer chains. These radicals then recombine to make covalent bonds, forming a stable 3D hydrogel network. The degree of cross-linking, mechanical properties, swelling, and hydrogel degradation rate can be precisely tuned by varying the radiation dose [213,214,216]. A notable application of radiation crosslinking is the development of wet dressings hydrogels based on CS-PEG, poly(N-vinyl-pyrrolidone) (PVP), polyethylene oxide (PEO), and poly (acrylic acid) (PAA), prepared through e-beam cross-linking in mildly acidic conditions (Fig. 28), the hydrogel was suitable for promoting accelerated healing and relieving pain in infected skin wounds [217]. The resulting hydrogels exhibit stability under simulated physiological conditions of an infected wound and maintain adequate moisture with a water vapor transmission rate of up to 272.67 g m^−2^ day^−1^, promoting accelerated healing. Furthermore, they exhibit a high loading capacity for ibuprofen (IBU), capable of including a therapeutic dose for managing severe pain. These hydrogels provide optimal wound healing conditions by maintaining moisture, promoting cellular proliferation, and minimizing infection risk. They also display excellent durability, slow degradation rates, and superabsorbent behavior under simulated hyperthermia (37–41 °C) and varying pH levels. Notably, the hydrogel morphology remained unchanged after 48 h in all test media, showing no signs of disintegration. Overall, these findings indicate that CS hydrogels crosslinked via e-beam radiation possess improved biocompatibility and mechanical properties, making them promising contenders for TE and advanced wound care [215,216].

#### 4.2.6 Comparative analysis of crosslinking strategies for CS-SIHs

Among the various crosslinking strategies employed to fabricate CS-SIHs, chemical and physical crosslinking remain the most fundamental. However, ionic, enzymatic, and radiation-induced crosslinking methods are increasingly gaining attention for their application-specific advantages. Table 4 summarizes the core features of these crosslinking strategies and guides their appropriate selection for different biomedical applications [[218], [219], [220], [221], [222]].

**Table 4:** Comparative overview of crosslinking strategies in CS-SIHs.

Method	Mechanism	Advantages	Limitations	Applications	Ref.
**Chemical**	Covalent bond via bifunctional crosslinkers	Strong mechanical strength, tunable degradation	Residual toxicity (e.g., glutaraldehyde)	Bone/cartilage TE	[218]
**Physical**	Hydrogen bonding, hydrophobic, or thermal interactions	Mild, injectable, no chemical reagents	Weaker mechanical properties, fast degradation	Ocular, neural, or injectable systems	[219]
**Ionic**	Electrostatic interaction with counterions	Rapid gelation, simple reagents	pH-sensitive, less durable	Nasal/ocular delivery, oral systems	[220]
**Enzymatic**	Enzyme-mediated formation of covalent bonds	Biocompatible, specific, green	Expensive, slower kinetics	In situ gels, wound healing	[221]
**Radiation**	Free radical formation by UV/gamma rays	No chemical residues, sterilization possible	Requires specialized equipment	Injectable systems with sterilization demand	[222]

## 5 Scaffold design and fabrication

### 5.1 Scaffold design principles

Successful tissue regeneration and integration with host tissues depend on the meticulous design of scaffolds, carefully considering their architectural and mechanical characteristics. Porosity is the most critical design parameter, which directly affects the scaffold's capability to support cell infiltration, vascularization, and nutrient exchange. High porosity facilitates optimal cell migration, tissue development, and the efficient exchange of oxygen, nutrients, and metabolic waste between cells and their surrounding environment. However, excessive porosity may compromise the mechanical integrity of the scaffold, making it unable to support tissue development effectively. Therefore, achieving a delicate balance between porosity and structural stability is essential. In most TE applications, scaffolds must have a carefully controlled porosity that allows for cell proliferation and tissue ingrowth while providing enough structural support to maintain their shape during tissue regeneration [223,224].

Another key parameter is pore size, which significantly affects cell behavior, tissue formation, and vascularization. However, the optimal pore size varies based on the target tissue, ranging from 100 to 500 μm. Smaller pores are generally suitable for soft tissues such as skin or cartilage, whereas larger pores are necessary for bone or other hard tissues. Proper pore sizing promotes cell-scaffold interactions, enhances ECM formation, and facilitates nutrient diffusion. However, oversized pores may compromise structural integrity, while undersized pores can hinder cell infiltration and tissue integration. Thus, identifying an optimal pore size is critical to balancing mechanical support with biological function [16].

Structural integrity remains a fundamental consideration, as scaffolds need to withstand in vivo mechanical stresses while supporting newly formed tissue. These forces may arise from surrounding tissues, physiological loading, or dynamic motion. Scaffolds with inadequate mechanical strength risk premature degradation or collapse under pressure, which can hinder tissue regeneration and compromise the scaffold's ability to perform its intended function. Mechanical properties can be finely tuned by adjusting parameters (e.g., material composition, crosslinking density, and scaffold geometry). For example, increasing the crosslinking density typically improves the stiffness and resistance to deformation. In contrast, lower densities may offer greater flexibility and faster degradation rates, which may be advantageous for specific applications. Material selection also plays a pivotal role; natural polymers such as CS can be chemically modified or blended with synthetic components to enhance their load-bearing capacity and durability [225,226].

Lastly, biodegradability and degradation kinetics are essential design factors that must align with the timeline of tissue regeneration. Ideally, the scaffold needs to degrade in tandem with new tissue formation, gradually transferring mechanical load to the regenerating tissue as it matures. Rapid degradation may result in a loss of structural support, whereas slow degradation may hinder tissue remodeling and provoke chronic inflammation. The degradation profile can be modulated through factors such as the scaffold's composition, crosslinking degree, and porosity [227,228].

### 5.2 Fabrication techniques

The precise control of design parameters enabled by advanced manufacturing processes, including freeze-drying, electrospinning, and 3D printing, facilitates the development of customized scaffolds tailored to the special needs of various tissue types. These technologies allow fine-tuning scaffold architecture at both macro- and micro-scales, enhancing their performance across various TE applications.

#### 5.2.1 Freeze-drying

Freeze drying, also referred to as lyophilisation, is a commonly adopted method for generating porous hydrogel scaffolds, particularly in TE. The technique typically begins with the cryogenic freezing of a hydrogel solution, commonly composed of CS, water, or another solvent. As the temperature decreases, the solvent precipitates ice crystals that disperse evenly throughout the hydrogel matrix. Subsequently, the system is subjected to vacuum conditions, initiating the sublimation phase, during which the ice directly transitions into vapor without passing through the liquid state. The sublimation process yields a highly porous scaffold that closely resembles the spatial arrangement of the original ice crystals. By modulating parameters (freezing rate and drying temperature), the porosity and pore size distribution can be precisely tailored, making freeze-drying a highly adaptable method for scaffold manufacture in TE applications [229].

An inherent benefit of freeze-drying lies in its ability to create interconnected pores with well-controlled dimensions, which is critical for facilitating efficient cell infiltration, nutrition diffusion, and waste elimination inside the scaffold. These structural features support cell migration and proliferation, facilitating tissue formation and maturation. Given the intrinsic biocompatibility, biodegradability, and tunability, the method offers substantial potential across diverse tissue types. By tailoring parameters (e.g., CS concentration, freezing rate, and sublimation pressure), the pore structure can be customized to fit specific applications. For instance, a slower freezing rate tends to produce larger pores due to the formation of bigger ice crystals, which is beneficial for applications (e.g., bone or cartilage regeneration) where significant cell infiltration or vascularization is required. In contrast, a faster freezing rate yields smaller pores that are more suitable for soft tissue regeneration, such as in skin or neural applications. Furthermore, the mechanical characteristics of freeze-dried CS scaffolds can be fine-tuned. Although the high porosity from lyophilisation often leads to reduced mechanical strength, this can be mitigated by introducing crosslinking agents or combining CS with other polymers. Such modifications enhance scaffold durability while maintaining the necessary porosity for biological function. Crosslinkers such as GP or glutaraldehyde can improve the structural integrity of freeze-dried CS scaffolds, thereby increasing their suitability for load-bearing applications or bone TE [230].

**Fig. 29:**
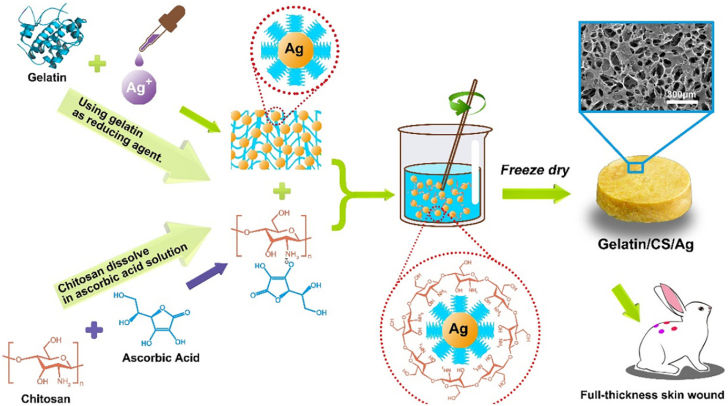
Fabrication of porous GEL/CS/Ag composite hydrogels [233]. Copyright 2019. Reproduced with permission from Elsevier.

Reys et al. produced CS-based hydrogel scaffolds by freeze-drying for cartilage TE [231]. The resulting scaffolds exhibited a highly porous structure that supported chondrocyte adhesion and proliferation. By carefully controlling freeze-drying conditions, cells were successfully sized within the optimal range (100–200 μm), promoting cell migration and ECM deposition. These scaffolds demonstrated excellent porosity and adequate mechanical properties, positioning them as promising candidates for cartilage regeneration. Moreover, freeze-drying has also been employed to generate post-synthesis porosity in nonporous PAAm/CS hydrogels. This involves immersing the hydrogel in water before lyophilization [232]. The morphology of the resulting hydrogel was affected by both the cross-linker ratio and pH of the synthesis mixture. Consequently, a hydrogel synthesized at pH 5 with a 1/80 cross-linker ratio shows pore sizes between 25 and 30 μm. Another study reported the development of novel porous GEL/CS/Ag composites using freeze-drying swollen gels created by combining GEL/Ag with CS and TA as a crosslinker (Fig. 29) [233]. From SEM images indicating microscopic surface and cross-sectional views, the authors demonstrated that GEL/CS, GEL/CS/Ag1, GEL/CS/Ag2, and GEL/CS/Ag3 scaffolds exhibited a dense, interconnected porous structure with interpenetrating pores. Moreover, the pore sizes of all the GEL/CS/Ag composite hydrogels were consistent, ranging from approximately 100 to 250 μm [233].

#### 5.2.2 Electrospinning

**Fig. 30:**
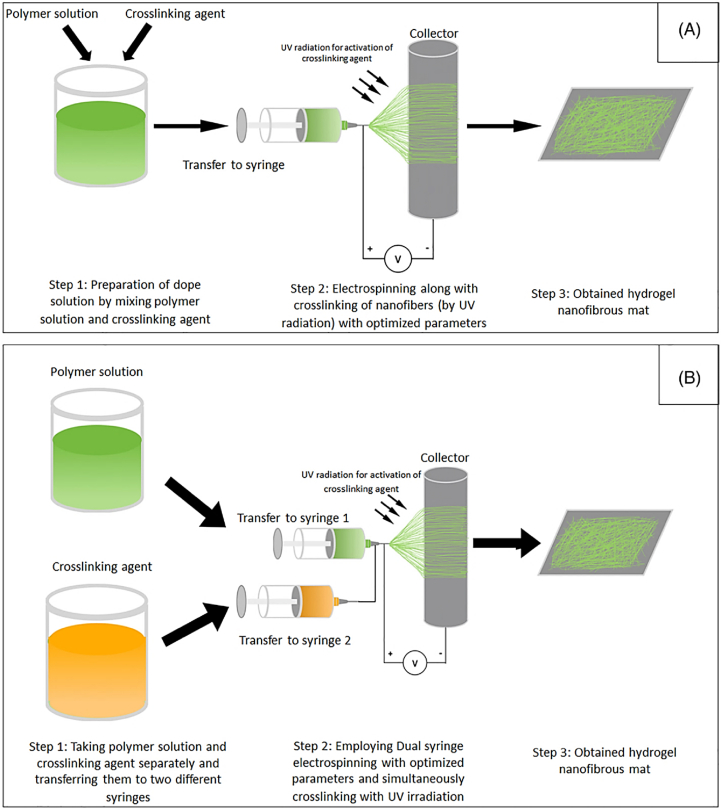
Single-step cross-linking technique for manufacturing hydrogel nanofibers [237]. Copyright 2021. Reproduced with permission from John Wiley & Sons.

Electrospinning is extensively used in manufacturing nanofiber scaffolds, namely in CS-SIHs used for TE purposes. This method uses a high-voltage electric field to apply force to a polymer solution or melt, causing the polymer to form a fine jet that stretches and thins into nanofibers as it travels toward a grounded collector. The yielded nanofibers are then deposited onto the collector to create a scaffold characterized by a high surface area, interconnected pores, and tunable porosity [234,235]. The concept of "reactive electrospinning" was initially proposed by Kim et al. [236] to prepare hydrogel nanofibers. In the single-step cross-linking approach, chemical cross-linking agents may be integrated in two primary ways. The first technique dissolves the cross-linker into the solution of polymer precursor, which is subsequently activated by UV light during fiber creation (Fig. 30A) [237]. The second method, the dual syringe technique, employs two syringes, one for the polymer solution and the other for the cross-linking agents (Fig. 30B) [237]. This method is used when the cross-linking agent reacts rapidly with polymer chains, potentially leading to premature 3D cross-linked structures that hinder electrospinning. To mitigate this, a second syringe is employed simultaneously to deliver the cross-linker alongside the polymer solution. A major advantage of electrospinning lies in its precise control over fiber diameter, which can range from a few nanometers to several micrometers, as well as its ability to tailor the overall structural design of the scaffold. This approach allows scaffold fabrication that closely replicates the natural ECM's mechanical and structural properties, essential for promoting cell adhesion, differentiation, and proliferation. Moreover, the high surface area of electrospun scaffolds provides enough space for cellular attachment and growth, making them highly beneficial for tissue regeneration [238,239]. By altering parameters (e.g., polymer content, applied voltage, and the spacing between the needle and collector), it is feasible to customize the porosity and pore size of the electrospun scaffold. The customizable features facilitate optimal nutrients and waste, which is crucial for tissue development. In CS-SIHs, electrospinning is especially advantageous because it incorporates growth factors, bioactive molecules, and other components that enhance tissue regeneration and repair. The synergy of CS's intrinsic biocompatibility, antibacterial properties, and biodegradability with the fibrous architecture produced by electrospinning makes this method well-suited for cartilage, bone, and skin TE applications. Furthermore, blending CS with other polymers (e.g., polycaprolactone (PCL) or PEO in composite scaffolds significantly enhances their mechanical strength while maintaining biocompatibility [239].

#### 5.2.3 D printing

3D printing, or additive manufacturing, is an emerging and highly versatile approach for fabricating scaffolds, particularly CS-SIHs, for TE [240,241]. Using precise computer-aided design (CAD) models, this technique allows for the layer-by-layer deposition of hydrogel materials to generate scaffolds with highly tailored and intricate geometries. Such precision in scaffold design is crucial for replicating the complex architecture of native tissues, which is fundamental to achieving successful tissue regeneration [240]. A major benefit of 3D printing is its exceptional ability to tailor scaffold structures to conform to the specific needs of the target tissue. Customized CAD models enable fine control over scaffold dimensions, configuration, and pore dispersion. This control level directly affects the scaffold's mechanical and biological performance. It ensures optimal mechanical support while allowing ample space for cellular infiltration, proliferation, and ECM deposition. Furthermore, 3D printing facilitates the direct integration of bioactive compounds, growth factors, or even living cells into the scaffold during fabrication. This capability is especially valuable in tissue-specific applications, enabling the creation of scaffolds that actively promote the regeneration of tissues (bone, skin, or cartilage). Incorporating bioactive proteins into the scaffold increases its ability to modulate cellular behavior, enhancing tissue integration and healing [241].

**Fig. 31:**
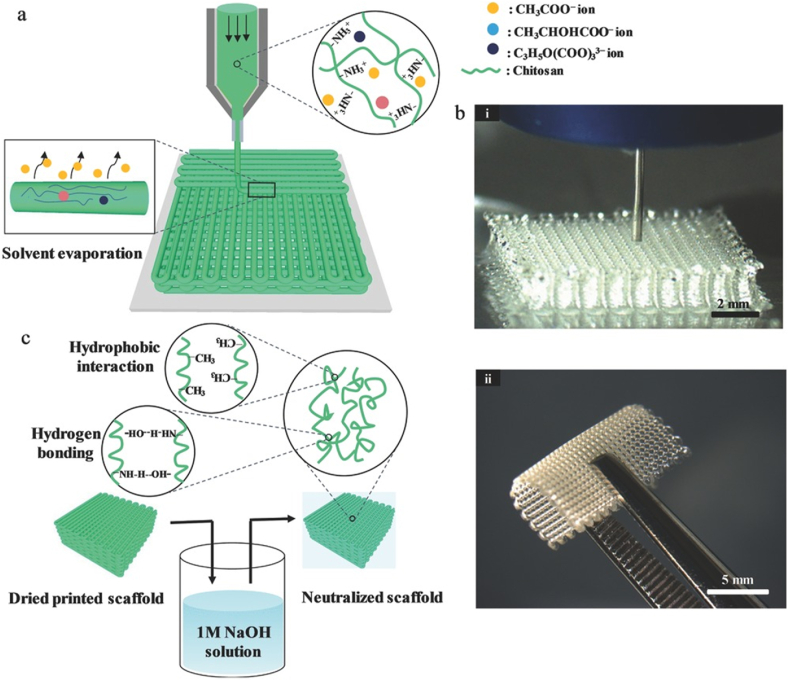
(A) 3D printing of a CS ink created using an acidic mixture and partially solidified through solvent evaporation. (b) (i) Photograph showing a 30-layer 3D printing of CS scaffold using a 100 μm micronozzle, and (ii) Photograph of a 10-layer CS scaffold fabricated with a 100 μm micronozzle and folded by a tweezer. (c) Representation of the neutralization process, leading to physical gelation through hydrophobic interactions and hydrogen bonding to form a CS hydrogel scaffold [245]. Copyright 2017. Reproduced with permission from John Wiley & Sons.

The flexibility of 3D printing is especially advantageous for generating scaffolds with precisely regulated mechanical properties for CS-SIHs. Due to its inherent biodegradability, biocompatibility, and antibacterial characteristics, CS is a superior material for TE applications. Through 3D printing, CS can be combined with alginate, gelatin, or collagen to improve scaffold performance and fulfill the mechanical requirements of specific tissues. This versatility ensures the scaffold preserves its structural integrity throughout tissue regeneration while progressively deteriorating as new tissue develops [242,243]. Another significant advantage of 3D printing is its ability to manufacture patient-specific scaffolds. CAD models can be generated based on anatomical data obtained from imaging techniques, such as magnetic resonance imaging (MRI) or computed tomography (CT). This approach is highly beneficial for personalized medicine, as it enables the precise design of scaffolds that conform perfectly to the size and shape of a defect or injury site. Such custom fitting improves scaffold integration and minimizes the risk of failure. Unquestionably, 3D printing offers excellent adaptability and accuracy, establishing itself as a promising and transformative technology for producing CS-based hydrogel scaffolds in TE [244]. For example; Wu et al. [245], employed 3D printing technique to fabricate CS hydrogels having highly flexible and organized microfiberous networked structure. Fig. 31 illustrates the 3D printing method employed to fabricate the scaffold using CS-based ink. The CS-based ink was printed directly in the air and partially hardened due to solvent evaporation. Subsequently, the CS scaffolds were refined via physical gelation in a neutralization step. The 3D-structured CS hydrogel was used for guided cell growth [245].

## 6 Cell interaction and growth promotion

TE and regenerative medicine depend on CS-SIHs due to their proven effectiveness in interacting with diverse cell types. Since each cell type thrives under distinct conditions, CS-SIHs offer a flexible substrate that may be adjusted to meet specific biological requirements. Table 5 summarizes the benefits and drawbacks of cell types, including stem cells, fibroblasts, and chondrocytes, when combined with CS-SIHs in TE [[246], [247], [248], [249], [250], [251], [252]].

**Table 5:** Benefits and drawbacks of stem cells, chondrocytes, and fibroblasts in CS-based hydrogels for TE applications.

Cell type	Benefits	Drawbacks
**Stem cells**	▪ Creating a biomimetic environment resembling ECM, enhancing cell adhesion and differentiation. ▪ Mechanical properties can be tailored to suit the target tissue type. ▪ Biocompatibility and support for stem cell survival and proliferation.	▪ CS's biodegradability rate may not align perfectly with stem cell differentiation timelines. ▪ Risk of immune response or improper integration due to variability in CS's sources and properties. ▪ Need for controlled delivery of growth factors or biochemical cues to direct differentiation.
**Chondrocytes**	▪ Supportive matrix for chondrocyte growth and ECM production (e.g., GAGs, collagen type II). ▪ Tunable stiffness to simulate native cartilage, aiding repair. ▪ Biodegradable and biocompatible, support long-term cartilage development.	▪ Limited in vitro expansion; often requires supplementary bioactive cues. ▪ Difficulty in maintaining phenotype without biomechanical/biochemical stimuli. ▪ Long-term stability of mechanical properties may decline.
**Fibroblasts**	▪ Enhances fibroblast adhesion, migration, and collagen production, crucial for wound healing. ▪ Porosity facilitates efficient nutrient exchange and waste removal. ▪ Surface modification can further enhance fibroblast adhesion.	▪ Risk of fibrosis or scar tissue formation due to excessive collagen synthesis. ▪ Mechanical tuning is often required for optimal tissue integration. ▪ Limited to specific tissue types and ECM components production.

The remarkable pluripotency of stem cells, enabling differentiation into various tissue lineages, underscores their widespread use in TE. CS-SIHs support stem cell adhesion, differentiation, and proliferation by providing an ideal biomimetic microenvironment. These hydrogels can mimic natural ECM in chemical composition and mechanical properties, facilitating targeted differentiation. Notably, stem cells may differentiate into chondrocytes, osteoblasts, or neurons, respectively, making them suitable for regenerating bone, cartilage, and nerve tissue [[246], [247], [248]].

Fibroblasts, mainly collagen, play a vital role in tissue repair through ECM synthesis. The precise adhesion, proliferation, and migration of fibroblasts within CS-SIH matrices are crucial for efficient tissue regeneration and wound healing. The fibroblast activity is influenced by the hydraulic permeability and surface characteristics of the hydrogels. Enhanced porosity improves nutrient diffusion and waste removal, promoting cell viability and proliferation. In addition, surface functionalization can enhance fibroblast attachment and collagen synthesis, thereby accelerating wound healing [249,250].

Chondrocytes which are responsible for cartilage formation, also benefit from the unique features of CS-based hydrogel. In cartilage TE, this hydrogel serves as a supportive scaffold that retains the phenotypic stability of chondrocytes while promoting ECM deposition, including GAGs and type II collagen. The mechanical characteristics of CS hydrogels can be altered to replicate the native stiffness of cartilage, further enhancing their potential for cartilage regeneration [251,252].

### 6.1 Growth factor incorporation

Integrating growth factors into CS-based hydrogels is a potent approach to increasing their efficacy in TE. Growth factors are crucial signalling molecules that control distinct cellular activities, such as migration, proliferation, and differentiation—key processes in tissue regeneration. Physical embedding is commonly used, where growth factors are directly added to the hydrogel matrix before gelation. As the hydrogel degrades, these molecules are gradually released, providing a sustained biological effect on the surrounding tissues. This technique ensures a consistent provision of growth factors, thus facilitating the process of healing. Another technique is chemical conjugation, involving the covalent attachment of growth agents to the hydrogel structure. This approach ensures a more consistent and regulated release of growth factors, enabling extended biological activity and minimizing the need for repeated applications. CS-based hydrogels can achieve superior therapeutic outcomes by tailoring these incorporation techniques, affirming their exceptional ability for healing and tissue regeneration [253].

Tissue regeneration also depends significantly on specific growth factors, such as fibroblast growth factor (FGF) [254], transforming growth factor-beta (TGF-β) [255], and VEGF [256]. FGF stimulates cell migration and proliferation, which are vital for tissue development and regeneration, whereas TGF-β is crucial for controlling ECM production and driving cell differentiation, making it indispensable in bone and cartilage repair [254,255]. In addition, VEGF is essential in developing new blood vessels (i.e., angiogenesis), which ensures an adequate supply of nutrients and oxygen to regenerate tissue [256]. Integrating these growth factors into CS-based hydrogels might greatly enhance TE applications, facilitating more precise and effective healing across a broad spectrum of clinical scenarios, from wound repair to organ regeneration.

## 7 Biomedical applications of CS-SIHs in regenerative medicine

CS-SIHs have gained significant attention in regenerative medicine due to their ability to mimic the ECM, enhance cellular functions, and enable minimally invasive delivery. Beyond their role as scaffolds, CS-SIHs serve as effective carriers for bioactive agents, including growth factors, antimicrobial drugs, and stem cell exosomes. In the context of regenerative medicine, CS-SIHs act as vehicles for the controlled release of bioactive compounds that promote tissue regeneration, confirming their classification within this therapeutic category.

### 7.1 Tissue regeneration applications

#### 7.1.1 Skin tissue engineering

CS-SIHs have significant potential in skin TE, particularly for managing acute and chronic wounds and fabricating skin transplants. CS's intrinsic biocompatibility, biodegradability, and non-toxicity properties make it particularly ideal for various biomedical applications. Applying CS-SIHs to a wound creates a moist environment, greatly improving wound healing by stimulating cellular migration, proliferation, and tissue restoration. This moisture prevents wound bed desiccation—a critical factor in effective tissue repair—and facilitates the recruitment of essential cell types, such as fibroblasts and keratinocytes, thereby accelerating re-epithelialization and tissue reconstruction. Furthermore, CS exhibits natural antimicrobial activity, providing a protective barrier against infection and inhibiting bacterial colonization at the wound site—a crucial advantage, particularly for chronic wounds such as diabetic ulcers. CS-based hydrogels also offer hemostatic benefits, as they interact with erythrocytes and thrombocytes to promote blood coagulation, effectively controlling hemorrhage at the injury site. This property is particularly beneficial in cases involving burns and traumatic injuries, where rapid hemostasis is essential. Moreover, these hydrogels can be tailored to control inflammation and organize ECM constituents, making them highly effective for managing skin lesions, from minor abrasions to severe burns and ulcers [247].

Ng et al. developed a gelatin-CS polyelectrolyte hydrogel optimized for 3D bioprinting at RT to enhance the shape accuracy of printed 3D structures while ensuring strong biocompatibility with fibroblast skin cells [257]. 3D bio-printing allows the precise placement of cells and matrices in a controlled 3D structure. CS is extensively applied in wound healing owing to its antibacterial and hemostatic characteristics. This study used gelatin-modified CS to produce printable polyelectrolyte gelatin-CS (PGC) hydrogels. The engineered skin constructs are temporary scaffolds to promote tissue regeneration while restoring the barrier function. Bioprinting allows for precise spatial placement of biomaterials, creating personalized grafts tailored to the wound's dimensions and depth. The results highlighted PGC hydrogels' potential for bioprinting applications [257].

**Fig. 32:**
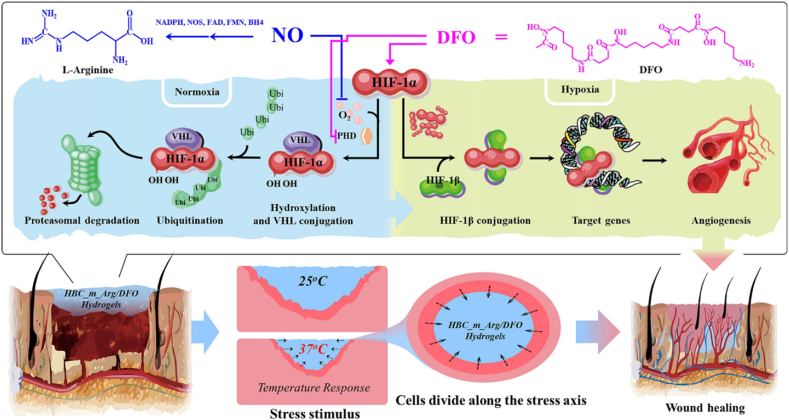
Schematic representation of the HBC_m_Arg/DFO hydrogel for improved wound healing. HBC_m_Arg/DFO hydrogel releases arginine and DFO to boost vascular regeneration, while its temperature-responsive shrinkage aids wound contraction [258]. Copyright 2024. Reproduced with permission from Elsevier.

Huang et al. developed a thermo-responsive and photo-cross-linkable hydrogel (HBC_m_Arg/DFO) by grafting hydroxybutyl CS methacrylate (HBC) with L-arginine and deferoxamine (DFO) (Fig. 32) [258]. This multifunctional hydrogel combines mechanical activity, vascular regeneration, and wound contraction to enhance healing. The hydrogel's temperature-responsive deformation induces self-contraction, boosting tissue regeneration and cellular proliferation. Additionally, DFO-mediated delayed arginine release improves wound maturity and promotes vascular regeneration. Quantitative assessments showed elevated HIF-1α, CD31, and VEGF levels, with superior vascularization and wound closure in HBC_m_Arg/DFO-treated groups. Cytotoxicity and subcutaneous implantation investigations confirmed the hydrogel's biosafety, affirming its suitability as an advanced wound dressing. Integrating mechanical stress modulation and regulated drug release highlights the functional superiority of HBC_m_Arg/DFO. This innovative approach provides a comprehensive solution for wound healing by simultaneously promoting vascular regeneration and facilitating wound contraction, establishing it as a strong candidate for advanced tissue regeneration therapies [258].

A study by Ma et al. introduced an injectable oxidized alginate/CMCS hydrogel (KA hydrogel) functionalized with silver-coated epigallocatechin-3-gallate (EGCG) (AE NPs) and keratin NPs (Ker NPs) to improve wound healing. AE NPs were incorporated for antioxidant activities by neutralizing free radicals and reducing oxidative stress during wound healing, while Ker NPs contributed to promoting epithelialization. The injectable composite hydrogel was synthesized via a Schiff-base reaction, enabling in situ gelation and structural stability [259]. The antioxidative properties of AE NPs were validated through radical scavenging studies employing ABTS and DPPH assays, which effectively neutralize ROS. Rheological analysis revealed a gelation time of ∼216 s and a storage modulus of ∼403 Pa. Modifying the concentration and oxidation level regulated gelation behavior, while Ker NPs increased the gelation time and reduced the storage modulus. AE NPs exhibited no alterations in gelation properties. In vivo, wound healing studies indicated that the KA hydrogel substantially accelerated wound repair, particularly in the first phases, and enhanced 21 % the thickness of the regenerated epidermis. The hydrogel demonstrated superior cytocompatibility and biocompatibility, underscoring its potential for therapeutic applications. Overall, the multifunctional KA hydrogel, integrating both antioxidant activity and epithelialization-promoting properties, represents a promising strategy for accelerating wound healing and advancing tissue regeneration therapies [259].

**Fig. 33:**
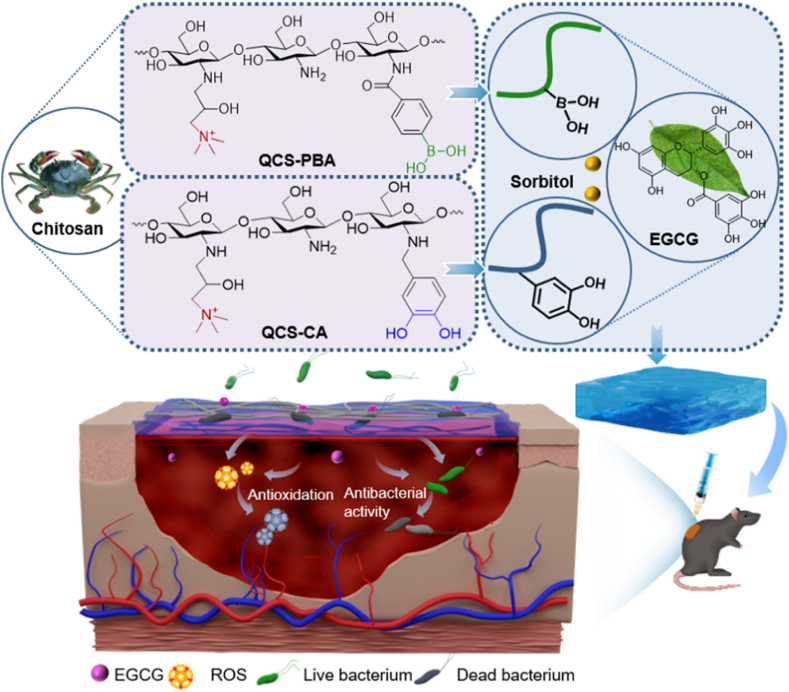
Preparation of QCS-PC hydrogel and its use in wound healing [260]. Copyright 2022. Reproduced with permission from Elsevier.

Zhong et al. produced a self-healing injectable hydrogel with dual antioxidant and antibacterial characteristics [260]. The hydrogel was synthesized by dynamic covalent bonding between catechol groups and boronic acid in QCS derivatives and subsequently functionalized with EGCG derived from green tea. This method yielded hydrogels (QCS-PC) with rapid self-healing capacity, shear-thinning characteristics suitable for injectability, and improved therapeutic performance. The QCS-PC hydrogels exhibited a resilient 3D network architecture established through boronated ester bonds, chain entanglements, and inter- and intra-molecular hydrogen bonding (Fig. 33) [260]. The catechol groups and encapsulated EGCG exhibited antioxidant activity by neutralizing free radicals and antibacterial properties by employing a "capture and kill" mechanism designed to combat bacterial infections. SEM images revealed a porous cross-linked structure within the hydrogels, essential for nutrient transport and cell infiltration. The hydrogel gelled within 30 s and exhibited pH-responsive EGCG release, enabling sustained therapeutic action in wound environments. In vivo, a full-thickness skin defect model revealed quick wound closure and regenerative recovery. The QCS-PC hydrogels effectively combine antibacterial and antioxidant bioactivities with dynamic mechanical properties, making them highly suitable for treating wounds with high microbial loads. This study emphasizes a viable approach for designing multifunctional, natural-based hydrogels for advanced wound care and tissue regeneration [260].

#### 7.1.2 Bone regeneration

CS-SIHs have emerged as a critical component in bone TE, providing an innovative technique to enhance bone regeneration and healing. Scaffolds that mimic the ECM and possess biodegradability and biocompatibility are optimal for promoting bone formation [261]. These 3D hydrogels, utilized as bone grafts or in conjunction with other biomaterials, promote cell adhesion, proliferation, and differentiation, creating an environment conducive to osteogenesis. By supporting the establishment of a new bone matrix, these scaffolds help restore the functional and structural integrity of the damaged bone. CS hydrogels encourage the spontaneous infiltration of osteoblasts—cells responsible for new bone development—and are often combined with bioactive agents (e.g., bone morphogenetic proteins (BMPs)) in bone regeneration due to their capacity to stimulate osteogenic differentiation. Among these, bone morphogenetic proteins 2 (BMP-2) has received significant attention for its ability to accelerate bone repair in large fractures by promoting the differentiation of MSCs into osteoblasts. The successful healing of bone is closely linked to the controlled release of bioactive chemicals from the hydrogel, ensuring prolonged and targeted therapeutic activity. By modulating hydrogel porosity and degradation rates, the release profile of osteoinductive agents can be fine-tuned to prolong delivery and maximize bone regeneration outcomes [262].

Integrating CS-SIHs with other biomaterials is a widely adopted approach to enhance their bioactivity and mechanical properties. For example, CS-hydroxyapatite composites have improved the mechanical stability of scaffolds and biological responses, including cell adhesion, differentiation, and proliferation, facilitating more effective bone regeneration. Hydroxyapatite, a mineral found naturally in bones, contributes to the mechanical strength and osteoconductivity of the scaffold when incorporated into the hydrogel matrix. This hybridization yields a composite that closely mimics the mineralized bone matrix, promoting seamless integration with host tissue and stimulating new bone growth [263]. Furthermore, regenerative advantage arises from the ability of CS-SIHs to encapsulate and support MSCs. The bioactive components of the CS matrix stimulate MSCs implanted within the hydrogel scaffold to differentiate into osteoblasts, thereby accelerating the repair of bone defects. This cell-based technique is highly effective for treating critical-sized bone lesions where endogenous repair mechanisms may be insufficient. CS hydrogels significantly promote bone tissue regeneration by providing a supportive microenvironment that supports stem cell growth and osteogenic differentiation [264].

Additionally, CS-SIHs promote angiogenesis, a critical process for delivering oxygen and nutrients to regenerating bone tissue. Successful bone repair relies on the interplay between osteogenesis and angiogenesis to sustain newly forming tissue. Incorporating VEGF) into CS hydrogels stimulates angiogenesis, ensuring adequate vascularization of the developing bone. The combined presence of osteogenic and angiogenic cues within CS-SIHs generates a synergistic effect, enhancing bone formation while establishing a supportive vascular network essential for sustained bone regeneration [265].

A biocompatible and thermosensitive CS hydrogel comprising graphene oxide (GO) and GlyP was developed and evaluated for its potential in bone healing [266]. The CS/GO hydrogel exhibited enhanced mechanical, physicochemical, and osteoconductive properties. Incorporating GO improved the structural integrity of the hydrogel and promoted cell adhesion and proliferation. The CS/GlyP/GO hydrogel was biocompatible with MSCs, which remained metabolically active following encapsulation. The hydrogel facilitated the osteogenic differentiation of mouse MSCs by upregulating the expression of alkaline phosphatase (ALP), runt-related transcription factor 2 (runx2), osteocalcin (OC), and type I collagen (COL-1) under osteogenic conditions. Rat bone marrow-derived MSCs (rBMSCs) were extracted for in vitro biocompatibility assays, whereas mouse MSCs were utilized for differentiation studies. Compared to conventional CS hydrogels, a histological study demonstrated increased bone matrix deposition and enhanced vascularization, promoting accelerated bone regeneration. This study highlighted that GO-incorporated CS hydrogels have significant potential to enhance mechanical properties and osteoinductive capacity. The developed constructs effectively promoted osteogenic differentiation of MSCs, demonstrating their suitability for bone TE applications.

Recently, the development of a bioactive scaffold by combining hydroxyethyl CS (HECS) with a PVA/biphasic calcium phosphate (BCP) nanoparticle-based hydrogel manufactured through cyclic freeze-thawing and subsequent in vitro biomineralization utilizing cell culture medium has been reported [267]. Fluorescence imaging showed that the biomineralized hydrogel exhibits superior cytocompatibility and compressive strength relative to the untreated hydrogel. Chemical modifications of CS with hydroxyethyl groups, along with biomineralization, significantly enhanced the mechanical characteristics of the scaffold, making the HECS-reinforced PVA/BCP hydrogel a suitable candidate for bone TE. BCP enhanced the osteoconductivity of the scaffold, hence improving compatibility with osteoblasts and other osteogenic cells. This study indicated that the enhanced HECS/PVA/BCP hydrogel, with favorable biological and mechanical characteristics, holds promise for bone regeneration applications [267].

#### 7.1.3 Clinical trials and commercial products

CS-SIHs are increasingly employed for their potential in tissue regeneration owing to their biocompatibility, biodegradability, and structural plasticity. Numerous clinical investigations have evaluated the efficacy of CS hydrogels in regenerating cartilage, wounds, and bone tissue. In clinical trials, CS hydrogels have demonstrated the ability to promote wound healing and decrease scarring, mimicking the ECM and enhancing cell adhesion, proliferation, and migration. Clinical investigations have also highlighted the potential of CS hydrogels, implanted with stem cells or growth hormones, for bone repair. The commercialization of CS-based hydrogels has expanded in parallel with their clinical application, and several products are now available [76,268,269]. Among the FDA-approved and extensively used options are HemCon® bandages, utilized in military and emergency medicine to control bleeding and promote healing. Products such as RegenOss®, developed for bone regeneration, combine CS hydrogels with bioactive compounds to enhance osteogenesis. Although challenges remain regarding long-term stability and large-scale production, the increasing inclusion of CS-based hydrogels in clinical trials and the commercial market underscores their substantial promise in regenerative medicine [270].

### 7.2 Wound healing

**Fig. 34:**
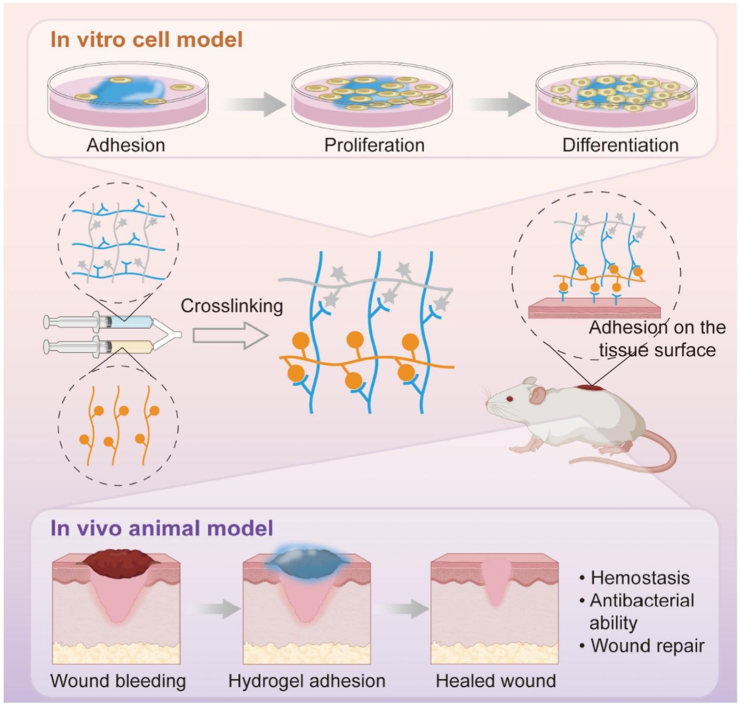
Wound healing applications of CS-based injectable hydrogels [181]. Copyright 2024. Reproduced with permission from Elsevier.

Wounds and skin breaks can be infected if pathogenic bacteria penetrate, leading to potential complications. Developing wound dressings with antimicrobial properties is crucial for effectively treating and protecting wounds, thereby reducing the risk of infection [132]. With the decline in novel antibiotic classes being developed, there is a need for functional wound dressings containing antimicrobial agents [265]. CS has been explored for its wound-healing properties due to its ability to accelerate healing by activating cells and promoting collagen formation. Additionally, CS-based materials are susceptible to degradation by bodily enzymes, further expediting the healing process. CS-based hydrogels have the potential to function as ideal dressings for wound healing due to their ability to foster wound contractions and healing, thereby acting as a wound-healing gas pedal with excellent biodegradability, biocompatibility, non-toxicity, and antimicrobial properties (Fig. 34) [181]. Commercially available CS-based wound dressings are available in various types, including non-woven fabrics, hydrogels, films, and sponges [38].

**Fig. 35:**
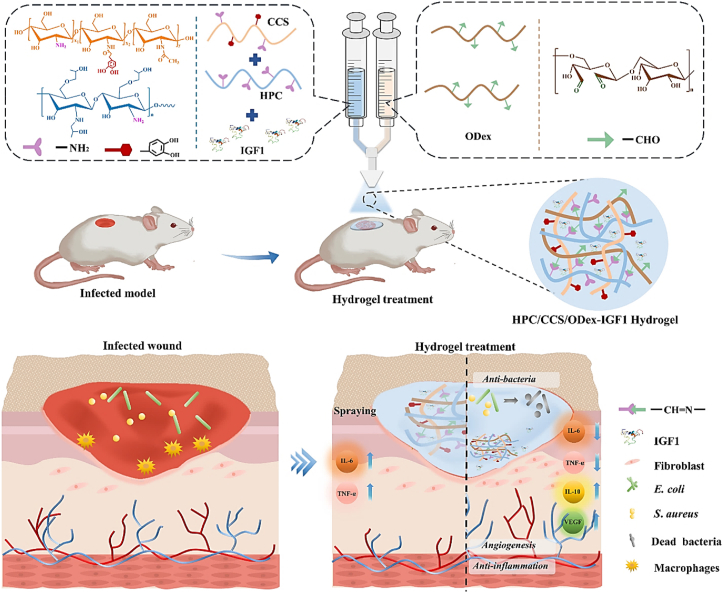
Sprayable CS-based hydrogels encapsulated with IGF1 to promote wound healing. Fabrication of the HPC/CCS/ODex-IGF1 hydrogel and its impact on enhancing bacteria-infected wound healing via anti-bacterial properties, anti-inflammatory effects, and angiogenesis etc., [137]. Copyright 2024. Reproduced with permission from Elsevier.

Tan et al. synthesized a sprayable hydrogel (HPC/CCS/ODex-IGF1) using hydroxypropyl CS (HPCS), oxidized dextran (ODex), and caffeic acid-functionalized CS (CCS), which was cross-linked via dynamic Schiff base linkages (Fig. 35) [137]. This hydrogel's pH sensitivity enables the release of insulin growth factor-1 (IGF1) in the acidic milieu of wounds resulting from bacterial infection. Apart from its remarkable antioxidant and antibacterial properties, the hydrogel displayed sprayable, self-healing, and self-adaptable characteristics. The catechol groups enhanced antibacterial and antioxidant properties, whereas the positively charged amino groups in CCS improved their sticky properties. Low cytotoxicity, hemocompatibility, and its capability to promote angiogenesis through cell proliferation and migration led to a remarkable 98.4 % recovery by day 11 in a full-thickness defect mouse model with bacterial infections. The hydrogel downregulated pro-inflammatory cytokines (TNF-α and IL-6) and raised cytokines associated with angiogenesis and anti-inflammatory responses, thereby lowering inflammation. With great promise for use in wound dressings, this multifunctional hydrogel has the potential to stimulate tissue regeneration [137].

**Fig. 36:**
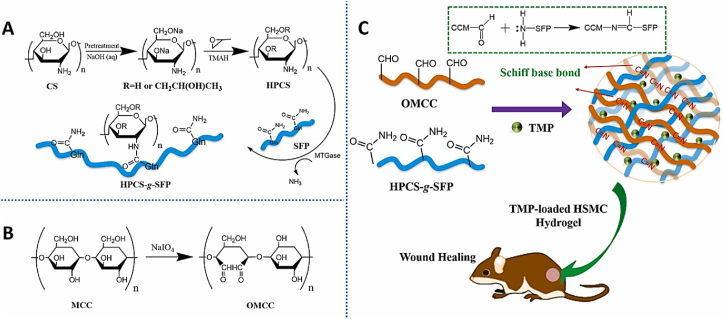
Injectable TMP-loaded HSMC hydrogel made using HPCS-g-SFP and OMCC. Synthesis process of (A) HPCS-g-SFP polymer and (B) OMCC polymer, and (C) preparation of TMP-loaded HSMC hydrogel through a Schiff base reaction [38]. Copyright 2022. Reproduced with permission from Elsevier.

Liu et al. manufactured an injectable hydrogel incorporating tetramethylpyrazine (TMP) to demonstrate scar less wound healing [38]. Fig. 36 shows the Schiff base interactions between oxidized microcrystalline cellulose (OMCC) and silk fibroin peptide-grafted HPCS (HPCS-g-SFP) in generating the hydrogel [38]. The HPCS-g-SFP copolymer significantly scavenges free radicals, specifically DPPH (2,2-Diphenyl-1-picrylhydrazyl) and hydroxyl radicals. The features of the hydrogel, such as pore size, gelling duration, water retention, and swelling rate, were altered by varying the ratios of OMCC to HPCS-g-SFP. A TMP hydrogel was added to human MSCs (HMSCs). In vitro experiments showed that the TMP-loaded hydrogel sustains 95 % cell viability for 24 h when co-cultured with hypertrophic scar fibroblasts (HSFB) or human skin fibroblasts (HSF). Animal studies demonstrated that hydrogel promotes scar-free wound healing by reducing scar formation and accelerating wound recovery [38].

### 7.3 Preclinical and clinical evaluation of CS-SIHs

CS-SIHs have shown exceptional promise in translational medicine, owing to their biocompatibility, biodegradability, and tunable physicochemical properties. Although their potential for biomedical applications has been extensively explored in vitro, growing focus is now being placed on validating their efficacy and safety in preclinical animal models and clinical trials. This shift marks a critical advancement toward the clinical translation of CS-SIHs.

#### 7.3.1 Preclinical studies

Qian et al. developed a multifunctional injectable hydrogel for cancer therapy, incorporating CS, indocyanine green (ICG), and CaO_2_ NPs. The hydrogel displayed self-healing and thermoresponsive properties, enabling controlled release and targeted photothermal and calcium overload treatments. In vivo murine tumor models demonstrated complete tumor ablation without causing systemic toxicity, highlighting the substantial potential of this approach for minimally invasive cancer treatment [271]. Jin et al. engineered a thermosensitive injectable hydrogel for cancer therapy, utilizing a PLGA-PEG-PLGA copolymer combined with low-molecular-weight CS and infused with the natural anticancer agent corilagin. The hydrogel went through a sol-to-gel transition at physiological temperature, making it suitable for intratumoral injection. This formulation substantially inhibited tumor growth in a mouse 4T1 breast tumor model and markedly enhanced the anticancer efficacy of Abraxane® when used in combination. The synergistic effects of tumor matrix remodeling by corilagin and improved cellular uptake facilitated by CS led to increased paclitaxel accumulation in tumor tissue, reduced expression of matrix proteins (fibronectin, collagen I, α-SMA), alleviated solid stress, and enhanced apoptosis. The system demonstrated excellent biodegradability, biocompatibility, and sustained corilagin release, positioning it as a promising injectable strategy for localized and combination cancer therapies [272].

Qian et al. developed an injectable, self-healing hydrogel composed of CS, silk fibroin, and platelet-rich plasma (PRP) to enhance tissue repair in diabetic wounds within the context of chronic wound healing. The hydrogel displayed shear-thinning behavior, resistance to enzymatic degradation, and bioresponsiveness, thereby facilitating the controlled release of growth factors from PRP. In both a type 2 diabetic rat model and a full-thickness skin defect model, the hydrogel significantly promoted wound closure, collagen deposition, neovascularization, and nerve regeneration. These findings were further supported by in vitro data showing enhanced chemotaxis and proliferation of MSCs. Collectively, these results highlight the hydrogel's potential as a versatile platform for addressing non-healing diabetic wounds by combining structural support with the bioactive delivery of therapeutic agents [273].

#### 7.3.2 Clinical evaluations and case Reports

Shi et al. constructed a thermosensitive, in situ gelling hydrogel using hexanoyl glycol chitosan (H-GCS) for the delivery of topical ocular therapeutics. This hydrogel undergoes a transition from liquid to gel at physiological temperature (32 °C), enhancing precorneal retention and drug absorption. The formulation exhibited favorable rheological properties and a porous internal structure (pores ranging from 50 to 200 μm), which facilitated the sustained release of encapsulated levofloxacin over 12 h. In vitro cytotoxicity assessments with human corneal epithelial cells and L-929 fibroblasts revealed negligible toxicity, with cell viability remaining above 80 % across a wide concentration range. Ocular safety was evaluated in rabbits using Draize testing, which showed no histopathological abnormalities or signs of distress following instillation. In vivo pharmacokinetic analysis demonstrated that levofloxacin delivered via the H-GCS hydrogel achieved a significantly higher maximum concentration (C_max_ = 3.50 ± 0.30 μg/mL) and area under the curve (AUC_0–12h_ = 11.89 ± 1.46 μg h/mL) in the aqueous humor, compared to conventional eye drops (C_max_ = 2.24 ± 0.28 μg/mL; AUC_0–12h_ = 6.18 ± 1.94 μg h/mL). This highlights a significant improvement in the retention and bioavailability of the drug. This work emphasize the therapeutic potential of glycol CS-based thermoresponsive hydrogels as safe and effective carriers for enhancing ocular drug delivery and patient compliance, particularly for the treatment of anterior eye segment disorders [274].

#### 7.3.3 Ongoing clinical trials

Despite substantial preclinical evidence supporting the medicinal applications of CS-SIHs, their clinical translation remains in the early stages. As of mid-2025, no registered clinical trials have been listed in major international registries assessing CS-SIHs for localized drug delivery, chronic wound healing, or regenerative therapies. This gap highlights the significant translational challenges faced by these systems, including issues related to material reproducibility, long-term biosafety, regulatory adherence, and the stability of formulations at clinical scales. However, the growing body of in vivo data, particularly from cancer, osteogenesis, and diabetic wound models, strongly supports the potential for future human studies. To bridge the translational gap, collaboration among researchers, clinicians, manufacturers, and regulatory bodies is imperative. Such collaboration will be crucial in developing standardized protocols and launching rigorously designed clinical trials to assess the efficacy of CS-SIHs in specific therapeutic applications.

#### 7.3.4 Clinical translation and regulatory challenges of CS-SIHs

Although CS-SIHs have shown considerable promise in preclinical studies, their progression toward clinical applications remain hindered by a range of scientific, regulatory, and manufacturing challenges. As of mid-2025, there are no major clinical trial registries listing Phase I-III trials directly evaluating CS-SIHs for indications such as localized drug delivery, chronic wound healing, or regenerative medicine. Nevertheless, several advanced preclinical studies conducted between 2023 and 2025 provide compelling evidence supporting their potential for human clinical translation. For instance, Zhu et al. developed a triple-responsive injectable CS hydrogel incorporating QCT-Cu NPs for the treatment of diabetic wounds. This hydrogel exhibited significant effects on angiogenesis, immunomodulation, and fibroblast homeostasis, underscoring its promising clinical applications [31]. Similarly, Lv et al. reported a thermosensitive crocin-1-loaded CS hydrogel, which markedly expedite the healing of full-thickness burn wounds by modulating reactive oxygen species and inflammatory pathways. These findings further exemplify the smart responsiveness and therapeutic potential of CS-SIHs across a range of pathological conditions [275].

In the field of cartilage repair, Shaygani et al. introduced an interpenetrating network of CS/silk fibroin hydrogel integrated with microspheres for sustained steroid delivery. Their approach resulted in near-complete defect filling and high chondrocyte viability after 12 weeks in vivo, demonstrating significant therapeutic promise [276]. In oncology, Tian and Yang reviewed the use of thermosensitive and bioadhesive CS hydrogels for bladder cancer treatment, highlighting their dual role as both drug carriers and potential immunomodulators. These studies exemplify how careful formulation optimization and multifunctionality can enhance preclinical efficacy, while also revealing translational challenges [32]. Additionally, Saadinam et al. explored the therapeutic potential of a thiolated CS/alginate hydrogel in spinal cord injury models, where it fostered axonal regeneration and diminished inflammation in rats. Although these results are promising, their translation into human clinical trials remains hindered by the complexity of neural repair and regulatory concerns surrounding biomaterials [277].

Despite significant progress, no CS-SIH formulation has yet achieved full regulatory approval. Key challenges include the inherent variability in the molecular weight and degree of deacetylation of CS, which complicate batch-to-batch reproducibility and make regulatory standardization difficult [32]. Furthermore, regulatory bodies such as the FDA and EMA place significant emphasis on the need for comprehensive long-term studies evaluating biocompatibility, degradation profiles, and product stability in injectable biomaterials. A limitation of many preclinical studies is the absence of large-animal validation or scalable Good Manufacturing Practice (GMP) protocols, which impedes clinical readiness [31,275]. To address these challenges, recent literature calls for the establishment of integrative translational pipelines. These include (1) humanized animal models that more accurately replicate disease microenvironments; (2) scalable, reproducible synthesis protocols; (3) adherence to ISO/USP biomaterial standards; and (4) the generation of robust pharmacokinetic-pharmacodynamic (PK-PD) data to support investigational new drug (IND) filings. Collaborative efforts among bioengineers, clinicians, and regulatory scientists are crucial for facilitating the transition of CS-SIHs from the research bench to clinical application.

### 7.4 Regeneration-targeted drug delivery via CS-SIHs

This section focuses on DDSs based on CS-SIHs that are specifically designed to support regenerative medicine applications, including delivering growth factors, exosomes, or antimicrobial agents to enrich tissue repair, angiogenesis, and wound healing. CS-based smart injectable gels have emerged as a potential option for targeted drug delivery and the treatment of specific pathologies. CS possesses unique properties, including biocompatibility and biodegradability, while CS-SIHs can act as DDSs, enabling localized, sustained, and responsive therapeutic release [78,[107], [108], [109],^278^]. CS-based hydrogels can be engineered/tailored to enable controlled drug release, which is vital for maintaining effective therapeutic concentrations over prolonged durations. The release rate can be controlled by altering the degree of crosslinking and the molecular weight of CS and by incorporating other polymers or NPs [278].

One of the most innovative features of CS-based smart gels is their responsiveness to physiological stimuli, including temperature, pH, and enzymatic activity. These hydrogels can be triggered to release drugs under specific conditions, thus enhancing therapeutic precision and minimizing off-target effects. For instance, pH-sensitive CS hydrogels can deliver drugs in the acidic tumor microenvironment, enabling tumor-specific therapy. This stimulus-responsive DDS treats the tumor directly while reducing damage to healthy tissues. Injectable CS hydrogels can be administered directly to the targeted area requiring treatment, facilitating precise drug delivery. This approach is a localized DDS and is especially beneficial for treating specific diseases, including cancer, infections, and inflammation. By delivering the medication directly to the affected area, one can concentrate more of the therapeutic agent exactly where needed, increasing treatment efficacy and decreasing side effects in unaffected body areas [[278], [279], [280], [281], [282]].

Adding NPs into CS-based gels can significantly improve their drug delivery capabilities. NPs can control the rate of drug release and make drugs more stable. For example, researchers have developed CS gels integrated with gold NPs, which gradually release cancer-fighting drugs. These gels have shown superior results in preliminary tests [[279], [280], [281], [282]]. CS-SIHs show great potential for targeted medicine, especially in cancer treatment, because they deliver drugs directly to tumors. These hydrogels can be engineered to release medicine upon encountering the acidic environment of tumors or certain enzymes, thus minimizing the detrimental effects of chemotherapy on healthy tissues while improving its effectiveness against cancer cells. Other promising DDS applications of CS-SIHs include gene delivery and gene therapy. Gene therapy has great promise to treat genetic disorders and other diseases. CS-based hydrogels can encapsulate genes, protecting them from deterioration and ensuring their delivery to the intended cells. Hydrogels can be designed to release genetic material in response to particular signals, providing effective and targeted gene delivery. In regenerative medicine, CS-based gels can transport growth factors, stem cells, and other regenerative agents to aid tissue repair and regeneration. Hydrogels create a favorable environment for cells to grow and develop, improving healing. For example, CS hydrogels loaded with stem cells have been used to repair damaged cartilage, demonstrating improved healing in animal studies [[283], [284], [285]].

CS exhibits inherent antibacterial properties, making CS-based hydrogels ideal for carrying antimicrobial agents. These gels can be employed to treat infections, particularly in wound healing. The controlled release of antibacterial agents from these hydrogels ensures a steady supply at the site of infection, promoting faster wound healing while reducing the risk of bacterial resistance to antibiotics. CS-SIHs have the potential to be used as personalized medicine in healthcare, offering targeted therapies for individual patients. By adjusting the hydrogel formulation to suit each patient's unique requirements, more efficient and targeted treatments can be developed. This approach necessitates an extensive understanding of the interactions between the hydrogel and the biological environment [[284], [285], [286], [287]].

### 7.5 Drug encapsulation and release

The efficacy of a hydrogel as a DDSs depend on its chemical and physical characteristics and the drug it carries. Choosing the appropriate hydrogel materials, their structure, and the method of drug incorporation is crucial to matching the drug's characteristics. Specific characteristics such as water resistance and electrical charge affect the release profile of drugs, particularly regarding sustained or rapid release. The main methods for drug incorporation into hydrogels include diffusion, entrapment, and tethering. The various drug loading and encapsulation strategies for CS hydrogels are outlined below. Each method has its own advantages and drawbacks, and the choice should depend on the hydrogel network type and the drug used [281].

**Physical Encapsulation:** This straightforward method involves incorporating the drug into a CS solution, followed by gel formation. The drug molecules become physically trapped within the hydrogel matrix as it solidifies. This technique is advantageous due to its simplicity and minimal chemical modification requirements [288,289].

**Chemical Conjugation:** In this approach, drugs are covalently bonded to the CS structure via strong chemical bonds. This method allows for controlled and sustained drug release, with the release rate governed by the degradation of chemical bonds. Chemical conjugation typically employs special agents to elevate the structural integrity and stability of the hydrogel [280,290].

**Ionic Gelation:** CS forms a jelly-like substance when interacting with negatively charged molecules (e.g., TPP). This method is particularly suitable for encapsulating hydrophilic drugs and can accommodate high drug payloads [291,292]. Embedding drugs within NPs and subsequently incorporating these NPs into a CS gel is possible. This approach combines the benefits of NPs drug delivery with the prolonged release characteristics of hydrogels, making it especially useful for poorly soluble drugs [293,294]. The release mechanisms for drugs from CS hydrogels vary, with the main types including:

**Diffusion-Controlled Release:** Drugs typically diffuse from CS hydrogels into the surrounding environment. The diffusion rate depends on various factors, including hydrogel porosity, drug molecule size, and the degree of entanglement within the matrix.

**Swelling-Controlled Release:** In this mechanism, the hydrogel swells in response to contact with body fluids, triggering the release of the encapsulated drug. The hydrogel composition and environmental factors (e.g., temperature and pH) influence the extent of swelling. Swelling-controlled release is especially beneficial for hydrogels that respond to specific physiological conditions.

**Enzymatic Degradation:** CS hydrogels can be establishment to degrade in the presence of specific enzymes, facilitating localized drug release. This method offers the advantage of delivering the drug to target areas with relevant enzymes, allowing for sustained and controlled release.

**Fig. 37:**
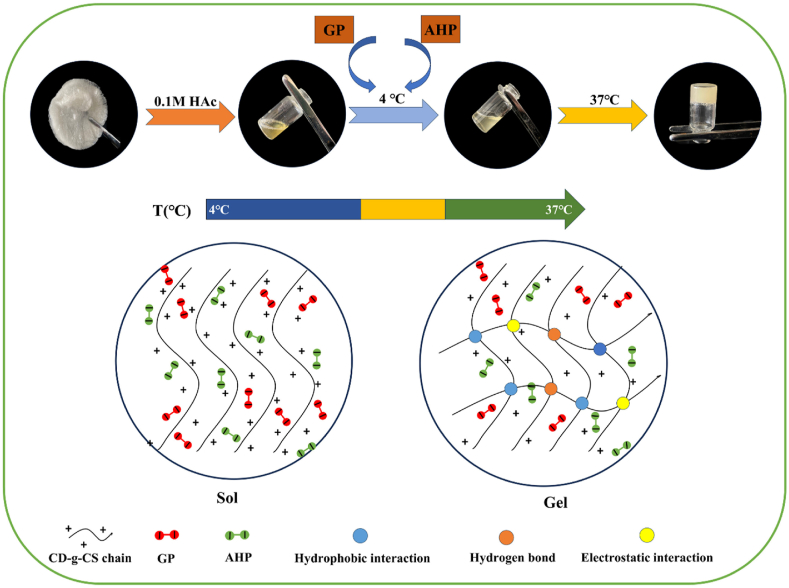
Schematic illustration of the CD-g-CS injectable hydrogel synthesis process with high thermoresponsive effects [297]. Copyright 2025. Reprinted with permission from Elsevier.

**pH-Responsive Release:** CS hydrogels can be tailored to react to variations in pH. In acidic conditions, the hydrogel matrix swells, causing the drug release. This property is beneficial for targeting specific areas in the digestive tract where pH levels fluctuate. Other stimuli-responsive release mechanisms, such as thermo-responsive and radiation-responsive, also exist [295,296]. A study by Jiang et al. stated the synthesis of thermoresponsive CS hydrogels grafted with β-cyclodextrin (CD-g-CS). The synthesis of CD-g-CS hydrogel is shown in Fig. 37 [297]. The hydrogel exhibited good thermal sensitivity and low cytotoxicity and was easily injectable. Notably, the hydrogel gelation time was less than 14 min, with a rapid setting time of 0.75 min. The hydrogel also showed significant water absorption and a degradation rate of over 65 % after 14 days. Furthermore, the hydrogel demonstrated a consistent and controlled release profile [297].

**Fig. 38:**
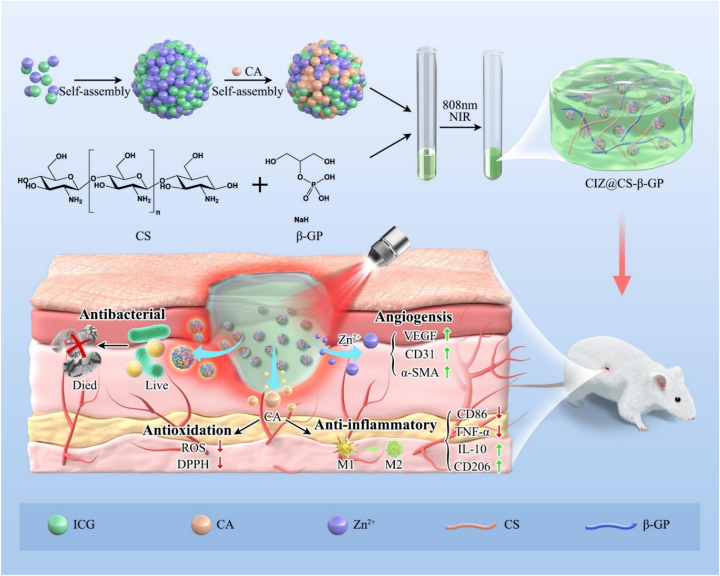
Illustration of injectable CS hydrogel embedded with antibacterial NPs for treating diabetic foot ulcers [298]. Copyright 2025. Reprinted with permission from Elsevier.

Wu et al. reported the synthesis and antibacterial applications of CS hydrogels self-assembled with multifunctional NPs [298]. A thermosensitive hydrogel, designed for smart therapeutic delivery, was created using self-assembled NPs. These NPs, composed of chlorogenic acid (CA), zinc ions (CIZ), and indocyanine green (ICG), were encapsulated within a CS-β-GlyP hydrogel, allowing for accelerated gel formation triggered by photothermal effects. The hydrogel demonstrated excellent biocompatibility and successfully facilitated the controlled release of drugs into diabetic foot ulcer wounds. Owing to the combined effects of zinc ions and CA, the hydrogel exhibited excellent antioxidant and anti-inflammatory properties, enhancing the expression of vascular endothelial growth factor (VEGF) and platelet endothelial cell adhesion molecule-1 (CD31), promoting angiogenesis. The in vivo as well as in vitro studies confirmed that CIZ@G successfully inhibits the growth of *Staphylococcus aureus* following laser irradiation and accelerates wound remodeling within 14 days. This study presents a novel approach for treating diabetic foot ulcers, potentially significantly improving patient care in this challenging clinical context. Fig. 38 depicts the schematic representation of the antibacterial effect and nanoparticle release mechanisms from the hydrogel [298].

**Fig. 39:**
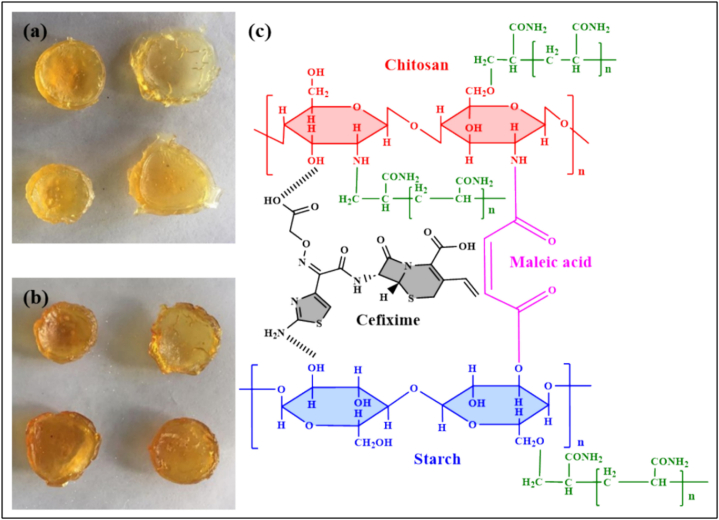
(A) CS-Starch hydrogel, (b) Cefixime-encapsulated hydrogel, and (c) Drug-loaded hydrogel structure [300]. Copyright 2024. Reproduced with permission from Elsevier.

In another recent study, Shajari et al. produced CS-based hydrogels for the drug delivery of timolol maleate [299]. They synthesized gels that rapidly form upon mixing oxidized gellan gum and CS with β-GlyP. Hydrogels OGCH1 and OGCH2, containing the highest amount of OGG, were chosen as the most promising formulations. In vitro testing revealed that OGCH1 exhibited faster drug release than the other hydrogels. OGCH1 demonstrated superior adhesion, flow properties, self-repair abilities, and sustained drug release compared to other hydrogels. This is particularly significant for ocular treatments, as the gel's ability to maintain the drug on the cornea for extended periods is crucial. Additionally, the MTT assays on bone marrow stem cells confirmed the biocompatibility of the hydrogels. Overall, the hydrogel presents a promising option for the controlled delivery of timolol in treating glaucoma [299]. Medha et al. [300], developed a hydrogel consisting of CS and starch which was made and characterized for drug delivery applications. Biomaterials with pH-responsive swelling properties are invaluable for controlled drug delivery. The hydrogel was evaluated for its expansion at a pH of 1.2 (simulating gastric conditions) and 7.4 (simulating intestinal conditions). The hydrogel demonstrated significant swelling over time, reaching its maximum size after 12 h. The synthesized CS-starch hydrogel showed high water absorption, pH responsiveness, blood compatibility, and biodegradability. BET analysis revealed that variations in the crosslinker concentration significantly influenced the porosity of the hydrogel. The composite CS-starch gel was explored as a potential contender for controlled drug release. Cefixime was incorporated into the hydrogel via a swelling and spreading method. SEM images confirmed that the drug molecules were evenly distributed throughout the hydrogel. The hydrogel demonstrated superior drug release at pH 7.4 (93.08 %) than at pH 1.2 (67.85 %), suggesting it can regulate drug release over an extended period (i.e., 12 h). The drug release followed the Makoid-Banakar and Korsmeyer-Peppas models using the Fickian diffusion technique. Additionally, the composite hydrogel showed fluorescence characteristics that changed with different light wavelengths, emitting the brightest blue light at 425 nm when excited at 350 nm. Furthermore, the cytotoxicity of the CS-starch hydrogel was assessed using the MTT assay on fibroblast cells (L929). The cell viability percentage showed no significant change with rising hydrogel concentration (50–1000 μg/mL). These results indicate that the hydrogel is safe for human cells and suitable for biomedical applications. The fibroblast cells maintained normal morphology and proliferated effectively with and without hydrogel, demonstrating the hydrogel's biocompatibility for cell survival and growth. Furthermore, the drug loading capacity of CS-starch hydrogel was evaluated using cefixime as a model drug, as shown in Fig. 39 [300]. The encapsulation efficiency (EE) was influenced by the amount of crosslinker, with EE values ranging from 63.03 % to 87.89 % for varying concentrations (0.18 g–0.22 g crosslinker). The optimized hydrogel (0.22 g crosslinker) encapsulated the maximum amount of cefixime, while higher crosslinker concentrations decreased EE due to reduced pore sizes. The drug release profile showed an initial rapid release (35.76 %) within the first hour, followed by a controlled, prolonged release over 12 h. Cefixime's absorption maximum (287 nm) remained unchanged throughout the release, indicating stable bioavailability. The release was pH-dependent, with higher release observed at pH 7.4 due to increased swelling and wider channels in the hydrogel. Despite cefixime's higher solubility in acidic conditions, the lower release at pH 1.2 was primarily due to the hydrogel's swelling properties. Kinetic modeling using the Korsmeyer-Peppas and Makoid-Banakar models revealed that drug release followed Fickian diffusion (n < 0.5) and was influenced by diffusion and swelling. The crosslinker concentration significantly impacted the release profile, with the highest release (93.08 %) observed at 0.22 g crosslinker. Optimal crosslinker concentration tunes the surface area and pore volume of hydrogels, thereby enhancing drug release [300].

**Fig. 40:**
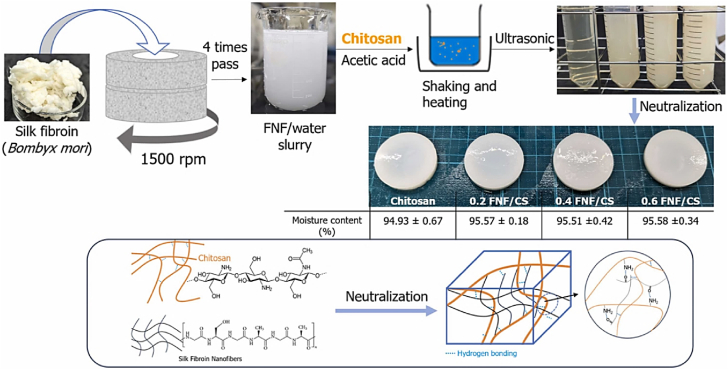
Synthesis process of fibroin nanofiber (FNF)/CS-based hydrogels [301]. Copyright 2025. Reprinted with permission from Elsevier.

Tran et al. prepared and characterized CS-based hydrogel scaffolds incorporated with fibroin nanofibers (FNF), as shown in Fig. 40 [301]. The CS and FNF/CS mixtures were made via a neutralization method using 10 % sodium hydroxide. Various fibroin nanofiber contents mixed with CS were gelled using neutralization. The mixed gels demonstrated excellent drug encapsulation (more than 25 %) and held significant amounts of medication (over 10 %). Controlled release of ibuprofen was achieved under specific conditions. The combination of fibroin nanofibers and CS holds promise for applications in bandages, drug delivery, and TE [301]. In another study, Zhang et al. created injectable hydrogels containing dual pharmaceuticals for precise sequential and long-term drug release, addressing the therapeutic needs of conditions such as acute retinal necrosis (ARN) [294]. The hydrogels were prepared by embedding CS-NPs coated with HA in an aminated hyaluronic acid (NHA)/aldehyde β-cyclodextrin (ACD) matrix. Two formulations, HA-DEX-CS-NPs/GCV-Gel and DEX-CS-NPs/GCV-Gel, were developed. Dexamethasone (DEX) was encapsulated in CS-NPs, while ganciclovir (GCV) was blended with the hydrogel matrix [294]. The hydrogels showed excellent swelling, gelation time, cytocompatibility, and degradation characteristics. In vitro, DEX-CS-NPs/GCV-Gel released DEX 128.5 % and 82.8 % faster than HA-DEX-CS-NPs/GCV-Gel over the first 24 and 48 h, respectively. HA-DEX-CS-NPs/GCV-Gel, however, slowed DEX release for the initial 7 h, allowing for a controlled release interval between DEX and GCV. The hydrogels sustained drug release for over 20 days, with macromolecule coatings offering flexibility in controlling release timing [294].

**Fig. 41:**
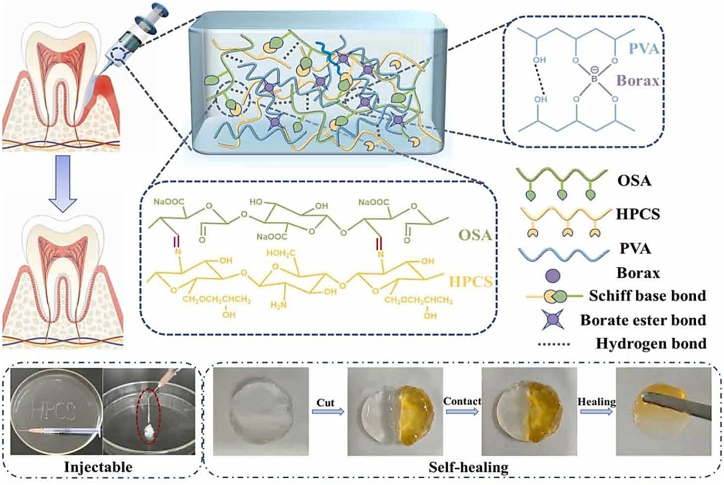
Synthesis and implementation of a unique injectable self-healing hydrogel made of hydroxypropyl CS/PVA that can be injected, self-healed when damaged, and fight germs. It is used for delivering medicine [302]. Copyright 2024. Reproduced with permission from Elsevier.

**Fig. 42:**
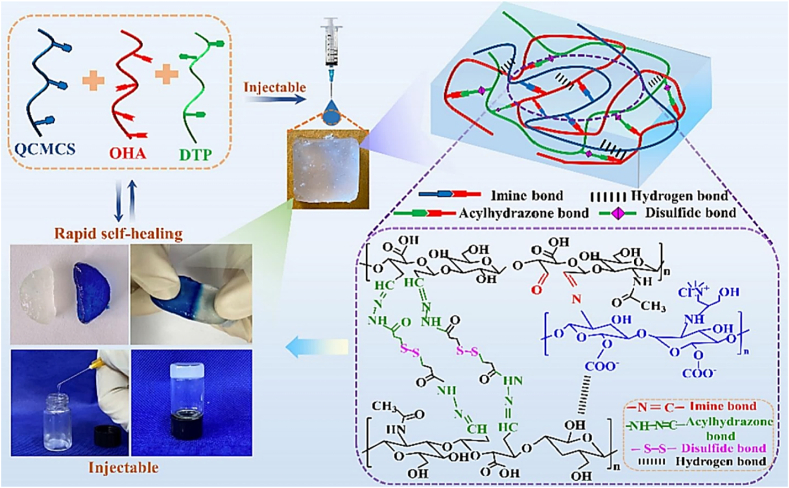
Demonstration of formation of QCMCS + OHA + DTP injectable hydrogel on the basis of dynamic covalent bonds (imine, disulfide, and acyl hydrazone bonds) and hydrogen bonds [162]. Copyright 2024. Reproduced with permission from Elsevier.

One of the current trends in the field of CS-SIHs is the introduction of self-healing features via novel methods, such as Schiff-base bond implementations. For example; Qiu et al. [302], prepared a special type of self-healing CS-SIH using poly (vinyl alcohol) (PVA) and hydroxypropyl CS as shown in Fig. 41 [302]. The hydrogel showed significant strength (about 18.20 kPa), was easily injectable, and self-healed with a PVA loading of 4 %. The hydrogel also demonstrated excellent antibacterial activity, with an antimicrobial effect of 87.94 % against Gram-negative *Escherichia coli* and 87.13 % against Gram-positive *Staphylococcus aureus*. The hydrogel was highly biocompatible, with over 80 % cell survival, could degrade over time, and exhibited a controlled drug release profile over 168 h. Thus, the hydrogel made in this study holds promise for drug delivery in gum disease treatment [302]. In another work, Zhang et al. developed a self-healing, injectable hydrogel using QCMCS, 3,3′-dithiobis (propionohydrazide) (DTP), and OHA [162]. The hydrogels were formed using a simple “one-pot” technique, using dynamic covalent bonds (imine, disulfide, and acyl hydrazone bonds) and hydrogen bonds as depicted in Fig. 42 [162]. The hydrogel demonstrated quick self-healing (96 % recovery in 30 min), good pH responsiveness (pH 4, 7.4, and 10), and excellent cytocompatibility (98 % cell viability). The hydrogel also exhibited notable mechanical strength with a compressive strength of 423 kPa and good injectability, forming a gel within 32 s. Moreover, the hydrogel displayed considerable swelling, controlled degradation, and sustained drug release. As a prototype drug, acetylsalicylic acid demonstrated prolonged release over 72 h, with kinetic modeling confirming a Fickian diffusion mechanism for the drug release. These results emphasize the potential of QCMCS-OHA-DTP hydrogels as pH-responsive DDSs. Their excellent mechanical characteristics, biocompatibility, and self-healing capabilities make them well-suited for biomedical applications [162].

Releasing drugs from CS-SIHs, such as sodium CS alginate, offers many benefits, including environmental sustainability and biocompatibility. Also, adding drugs to CS-SIHs facilitates their slow release, allowing bladder cancer medications to remain in the bladder for prolonged periods. Traditional hydrogels used for bladder cancer treatment face several challenges, such as poor adhesion, detachment during regular urination, and limited targeting efficiency, which lead to suboptimal drug delivery. Without better drug delivery strategies, treatment outcomes are often compromised. Chen et al. investigated a CS/dialdehyde SA/magnetic dopamine hydrogel to treat bladder cancer [303]. The hydrogel was synthesized by grafting, crosslinking, and compounding CS with sodium dialdehyde alginate and dopamine. A targeted drug-delivery system suitable for bladder cancer therapy was constructed [303]. The large number of catechol groups in the hydrogel ensures high adhesion to CS-SIHs. To enhance the ability of the hydrogels to target specific areas, iron tetraoxide (Fe_3_O_4_) NPs were incorporated [300,301]. The resulting drug-delivery system showed excellent features, including strong adhesion to organ walls, desirable hydrogel characteristics, effective targeting ability, 98 % antimicrobial and antibacterial activity, and 99 % biocompatibility. Given these outstanding characteristics, the developed targeted hydrogels hold significant promise for drug delivery applications and bladder cancer therapy [303].

**Fig. 43:**
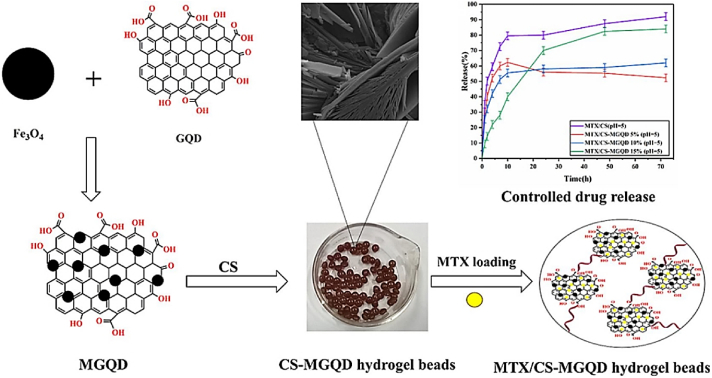
Formation mechanism of CS-MGQD hydrogel and methotrexate loading and release [304]. Copyright 2024. Reprinted with permission from Elsevier.

**Fig. 44:**
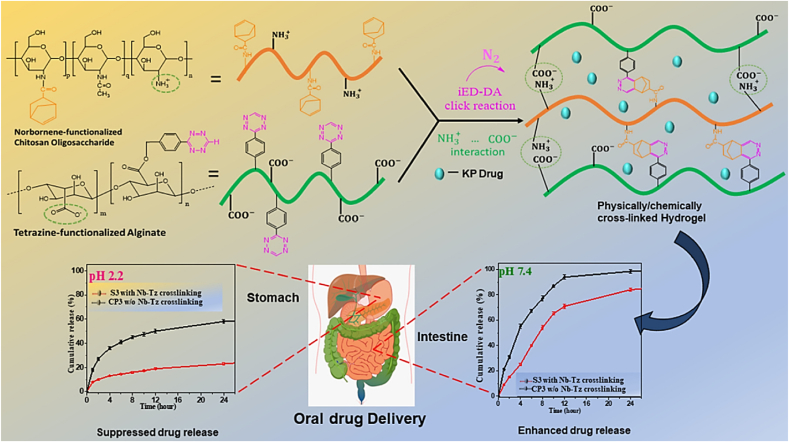
Synthesis mechanism and release properties of double-crosslinked CS/alginate hydrogels fabricated by the Nb-Tz “click” reaction for pH-sensitive DDS [305]. Copyright 2022. Reprinted with permission from Elsevier.

**Fig. 45:**
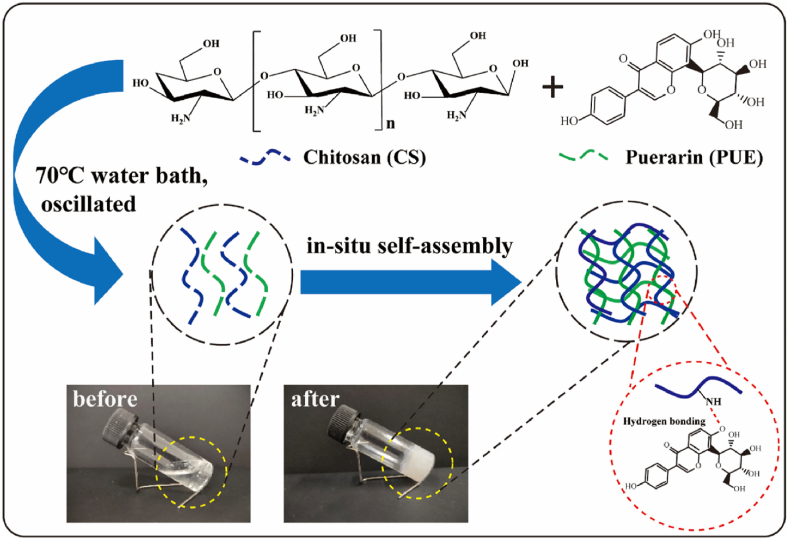
Schematics representing the synthesis process of injectable CS/PUE composite hydrogels employed in drug delivery applications [306]. Copyright 2021. Reprinted with permission from Elsevier.

Inducing magnetic properties in CS-SIHs is an effective strategy for producing magneto-responsive DDS capable of targeted drug delivery. In a study by Poursadegh et al. [304], a CS-magnetic graphene quantum dot (CS-MGQD) bio-nanocomposite hydrogel was synthesized and evaluated as a pH-responsive DDS. The bio-nanocomposite hydrogel was cross-linked utilizing a NaOH solution, and then CS hydrogel beads containing methotrexate were incorporated. The results demonstrated the CS-MGQD DDS's effectiveness in drug uptake and release in a laboratory setting. Fig. 43 indicates the formation mechanism of CS-MGQD hydrogel and methotrexate uptake and release [304]. Hydrogel beads also undergo pH-dependent swelling and release of methotrexate. The fabricated magnetic bio-nanocomposite hydrogel beads demonstrate exceptional potential for pH-sensitive, implantable DDSs, specifically for treating cancerous tissues [304]. In a recent study by Hoang et al. [305], click chemistry was used to prepare dual cross-linked CS-SIHs. A novel hydrogel, characterized by both chemical and physical double cross-linking, was synthesized from CS oligosaccharide and alginate (COS/Alg) utilizing a norbornene-tetrazine (Nb-Tz) click reaction to deliver ketoprofen. The chemical cross-linking achieved through the Nb-Tz reaction significantly enhanced the hydrogel's capacity for hydrophobic drug loading, resulting in an impressive 44 % weight/weight ratio of ketoprofen. This approach improved the loading efficiency and facilitated a sustained release profile. The mechanism underlying this sustained release is attributed to the hydrophobic interactions between ketoprofen and the polysaccharides used in the hydrogel formulation. The development of this double-crosslinked hydrogel represents a significant advancement in DDSs, particularly for hydrophobic drugs. The comprehensive evaluation of its properties indicates promising potential for widespread therapeutic applications, highlighting the importance of the Nb-Tz click reaction in enhancing drug loading and release characteristics in hydrogel formulations. Fig. 44 illustrates the primary mechanism of the dual cross-linked CS hydrogel preparation and the resulting drug release profile [305]. The development of these double-crosslinked hydrogels represent a significant advancement in DDSs, particularly for hydrophobic drugs. The comprehensive evaluation of its properties indicates promising potential for widespread therapeutic applications, highlighting the importance of the Nb-Tz click reaction in enhancing drug loading and release characteristics in hydrogel formulations [305]. In another study, CS hydrogels enhanced with injectable supramolecular nanofibers have been reported to exhibit significant antibacterial and anti-inflammatory effects [306]. Such developments indicate a noteworthy potential for improving drug delivery mechanisms. A composite hydrogel made from CS and puerarin (PUE_18_), utilizing an in-situ self-assembly method to create interpenetrating networks within the CS hydrogels, was examined. Fig. 45 displays the synthesis steps of injectable CS/PUE composite hydrogels [306]. The CS/PUE_18_ composite hydrogels exhibited enhanced mechanical properties (storage and loss modulus increased by three orders of magnitude) and pH responsiveness. Moreover, the resulting CS/PUE18 composite hydrogels showed various functional properties, including injectability, thixotropic behavior, strong cytocompatibility, biodegradability, and anti-inflammatory and antibacterial effects. The findings indicated that the CS/PUE18 composite hydrogels hold significant potential as injectable DDSs, facilitating controlled and sustained release of therapeutic agents [306].

Thus, over the years, researchers have demonstrated various innovative and potential biomedical applications of CS-based hydrogels and their corresponding systems, focusing on their mechanisms of action and responsiveness. These hydrogels have been developed for diverse therapeutic purposes, including antifungal and antimicrobial DDS, which utilize pH-responsive matrix swelling and erosion to enhance efficacy. For instance, the combination of CS with halogenated monoaldehydes demonstrates significant antifungal properties, while other formulations, such as Bletilla striata polysaccharide/CS, are designed for targeted rectal drug delivery with thermosensitive characteristics. In addition to their antimicrobial applications, CS hydrogels have been employed in targeted DDSs, particularly in antitumor therapies. The self-healing CMCS-HA system exemplifies a pH-responsive matrix that facilitates targeted delivery through swelling-induced mechanisms. Other notable examples include gallol-rich CS hydrogels and CS/γ-alumina/carbon quantum dot composites, which exhibit pH-responsive properties crucial for effective antitumor drug delivery. Furthermore, incorporating silver NPs into k-carrageenan/CS systems enhances their targeted anticancer capabilities. The versatility of CS-based hydrogels extends to various other applications, including anti-inflammatory and transdermal DDSs. For instance, CMCS and SA together have shown tremendous potential as an anti-inflammatory drug, while microneedle systems utilizing CMCS and pullulan demonstrate highly effective swelling and deswelling properties for transdermal applications. Additionally, formulations such as CS oligosaccharide/alginate and polyelectrolyte/CS/pectin have been developed for antioxidant and antibacterial purposes, showcasing the significant potential of these hydrogels in modern therapeutic strategies. Table 6 summarizes various biomedical applications of CS based hydrogels [123,[305], [306], [307], [308], [309], [310], [311], [312], [313], [314], [315], [316], [317], [318], [319], [320], [321], [322], [323]].

**Table 6:** A summary of recent works on CS-based hydrogels and relevant systems, their applications, response mechanisms, and therapeutic uses.

System	Application	Action trigger	Ref.
Self-healing carboxymethyl CS-HA	Targeted DDS	pH-responsive matrix swelling	[123]
CS oligosaccharide/alginate norbornene-tetrazine	Hydrophobic drug, KP DDS	pH-responsive	[305]
Supramolecular nanofiber-reinforced CS	Anti-inflammatory DDS	pH-responsive	[306]
CS and three halogenated mono-aldehydes	Antifungal, antimicrobial DDS	pH-responsive matrix swelling and erosion	[307]
Bletilla striata polysaccharide/CS	Rectal drug delivery	Thermosensitive	[308]
CS hydrogels	Antitumor DDS	pH-responsive	[309]
Succinoglycan/CS hydrogels	Antimicrobial DDS	pH-responsive	[310]
β-cyclodextrin/CS-based (polyvinyl alcohol-co-acrylic acid)	Antioxidant and antibacterial DDS	pH-responsive swelling	[311]
Gallol-rich CS hydrogel	Antitumor DDS	pH-responsive swelling/deswelling	[312]
CS/γ-alumina/carbon quantum dot	Antitumor DDS	pH-responsive	[313]
k-carrageenan/CS/silver NPs	Targeted anticancer DDS	pH-responsive	[314]
CS/crotonaldehyde	Hydrophilic DDS	pH-responsive	[315]
CS/fullerene	Antitumor DDS	pH-responsive	[316]
Carboxymethyl CS/SA	Anti-inflammatory DDS	pH-responsive	[317]
Carboxymethyl CS SFP/pullulan	Microneedle transdermal DDS	Swelling/deswelling	[318]
Polyelectrolyte/CS/pectin	Antioxidant and antibacterial DDS	pH-responsive	[319]
CS-gelatin	Oral DDS	pH-responsive	[320]
CS-dopamine-inulin aldehyde	Localized DDS	pH-responsive	[321]
CS microspheres/hydroxypropyl chitin	Cartilage repair DDS	Thermo-sensitive	[322]
CS/PEG	Antibacterial and anti-inflammatory DDS	Thermo-sensitive	[323]

### 7.6 Cartilage and bone tissue engineering with CS-SIHs

CS-SIHs hold considerable promise in TE, owing to their biocompatibility, biodegradability, and structural similarity to the natural ECM. Recent advancements have expanded the application of these materials to cartilage and bone tissue engineering (BTE), highlighting the crucial roles of both mechanical support and biological signaling. Injectable CS hydrogels with chondrogenic signals effectively promote chondrocyte proliferation and ECM accumulation, thereby facilitating cartilage regeneration. Tang et al. demonstrated that CS hydrogels exhibited compressive strengths ranging from 10 to 50 kPa, comparable to that of natural cartilage, and promoted the expression of collagen type II and aggrecan in encapsulated cells [324]. The incorporation of BMP-2 or MSCs further enhances bone formation and repair through CS-SIHs. In vitro studies revealed a 2.5-fold increase in ALP activity and calcium mineralization over 14 days, indicating active bone formation. Shan et al. demonstrated that composite CS-based hydrogels exhibited compressive moduli ranging from 80 to 150 kPa, highlighting their suitability for non-load-bearing bone healing applications [325]. The combined mechanical and biological properties of CS-SIHs enhance their effectiveness as injectable scaffolds for musculoskeletal regeneration. Future innovations may further improve their functionality through biomimetic structural design and the integration of nanomaterials.

### 7.7 Quantitative performance metrics of CS-SIHs

This section outlines essential quantitative performance metrics that evaluate CS-SIHs in comparison to two commonly utilized biomaterials, PEG and alginate-based hydrogels, focusing on mechanical strength, degradation characteristics, and drug release profiles.

#### 7.7.1 Mechanical properties

The mechanical properties of CS-SIHs, crosslinked with agents such as glutaraldehyde (GP) or sodium tripolyphosphate, typically demonstrate compressive moduli in the range of 30–80 kPa. These values are influenced by polymer concentration, crosslinking density, and formulation conditions. GP-crosslinked CS injectable hydrogels exhibit a compressive modulus of approximately 45 ± 5 kPa, which is significantly higher than the ∼30 kPa observed in PEG-based hydrogels under similar swelling conditions [326,327]. In contrast, alginate-based hydrogels, lacking ionic reinforcement, typically show lower mechanical strength, with moduli ranging from 20 to 30 kPa [328]. The enhanced mechanical stability of CS-SIHs is decisive for their application in soft tissue regeneration and regenerative medicine while maintaining their structural integrity.

#### 7.7.2 Degradation behavior

The degradation kinetics of hydrogels are decisive in their applications, in particular for drug delivery and TE. CS-SIHs typically degrade for 7–21 days under enzymatic or physiological conditions, with the degradation rate being modifiable through adjustments in polymer composition and crosslinking strategies. In contrast, PEG hydrogels, due to their synthetic nature, exhibit enhanced resistance to enzymatic degradation and maintain stability for over 30 days. This prolonged stability, however, may be disadvantageous when rapid matrix resorption is required [65,329]. Alginate-based hydrogels, while showing a comparatively faster degradation rate (5–14 days), lack the inherent bioactivity characteristic of CS derivatives [68].

#### 7.7.3 Drug release profiles

CS-SIHs demonstrate remarkable adaptability for regulated and stimulus-responsive drug delivery. Doxorubicin-loaded CS-based injectable hydrogels released approximately 80 % of the drug within 72 h at a pH of 5.5, simulating the tumor microenvironment. Conversely, the release was significantly reduced at neutral pH, confirming the material's pH-responsiveness [330]. By comparison, PEG-based hydrogels typically exhibit non-stimuli-responsive behavior, resulting in a slower and less controlled release, with approximately 50 % release over 72 h [331]. Alginate hydrogels, while offering some stimulus-responsive characteristics, do not achieve the same degree of molecular modulation, releasing about 70 % of the payload over similar timeframes when enhanced with calcium ions or bioactive additives [64]. In addition, PEG and alginate-based hydrogels are two of the most widely used injectable biomaterials clinically. Although collagen and silk fibroin are known for their excellent biocompatibility, their insufficient mechanical strength and lack of intrinsic stimuli-responsiveness limit their independent application [332,333]. HA-based hydrogels, while biodegradable and biocompatible, often require chemical modifications to enhance stability or enable controlled release [334]. In contrast, CS-SIHs offer a unique combination of injectability, tunable mechanical strength, enzymatic degradability, and inherent bioactivity, including mucoadhesive and hemostatic properties. Moreover, the differences in mechanical strength, degradation rates, bioactivity, and drug release potential of various hydrogel-based materials summarized in Table 1 provides an insight on the quantitative understanding of the distinctive role of CS-SIHs within the contemporary framework of SIHs in the biomedical field.

### 7.8 Toxicological considerations of chitosan degradation products

A comprehensive toxicological evaluation of the degradation products and their interactions in specific pathological microenvironments—such as tumors, chronic wounds, or ischemic tissues—is essential, even with the acknowledged biodegradability and biocompatibility of CS. Lysozyme, the primary enzyme responsible for catalyzing the breakdown of CS, generates low-molecular-weight oligosaccharides, D-glucosamine, and N-acetylglucosamine. In acidic microenvironments, such as those found in solid tumors (pH 6.2–6.8), the depolymerization of CS is often accelerated, resulting in changes to the size, charge, and reactivity of its fragments [335].

Recent studies indicate that these degradation products exhibit some reactivity. Low-molecular-weight CS oligosaccharides activate macrophages in acidic or inflamed conditions via Toll-like receptor pathways, triggering the release of pro-inflammatory cytokines, such as TNF-α, IL-1β, and IL-6. The surface charge, molecular weight, and degree of deacetylation of the fragments have key role to play in modulating immune system responses. Failure to appropriately control degradation kinetics may lead to local or systemic inflammatory reactions. Additionally, the degradation byproducts of CS can interfere with tissue remodeling and healing if released at concentrations that exceed physiological thresholds. These issues are particularly relevant in cancer therapy and regenerative medicine, where maintaining immunological balance is paramount. In tumor models, CS degradation products have been shown to be associated with increased antigen-presenting activity in dendritic cells. While this holds potential advantages for vaccine delivery, it may pose risks for tissue reconstruction applications [336].

CS-based hydrogels and their breakdown products are cleared pharmacokinetically by the renal and hepatic systems. Preclinical studies suggest that low-perfusion tissues, such as tumors or cartilage, may retain the drug at the injection site for extended periods, particularly with continuous therapy. However, long-term biodistribution studies remain scarce. These findings underscore the importance of investigating local immune responses, as well as the rate of CS degradation and clearance, during prolonged therapeutic use [192]. To address these concerns, optimizing the molecular architecture of CS-SIHs is crucial for achieving predictable degradation profiles and minimizing the release of immunogenic fragments. Strategies such as modifying acetylation levels, incorporating anti-inflammatory co-polymers, and applying enzyme-responsive crosslinkers can effectively control degradation rates and improve immunological compatibility. The successful clinical translation of CS-SIHs depends on their efficacy, as well as the safety, bioresorbability, and metabolic clearance of their degradation intermediates, ensuring the absence of adverse effects.

## 8 Challenges and future perspectives

Despite their promising biomedical applications, CS-SIHs face substantial challenges in clinical translation and commercialization. The efficacy of CS-based hydrogels is heavily influenced by variations in the source of raw materials, molecular weight, and degree of deacetylation, which can lead to potential issues with reproducibility. Furthermore, the lack of standardized protocols for functionalization, crosslinking, and sterilization complicates regulatory approval and large-scale manufacturing. Additional hurdles include limited clinical trial data, formulation instability during storage, and challenges in scaling production to meet Good Manufacturing Practice (GMP) standards. Overcoming these barriers necessitates the adoption of standardized methodologies, ensuring early regulatory alignment, and fostering collaborative industrial efforts to unlock the full translational potential of CS-SIHs.

CS-SIHs have attracted significant attention, thanks to their unique characteristics, including biodegradability, biocompatibility, and responsiveness to various stimuli. These hydrogels offer promising applications for drug delivery, wound healing, and TE. Despite their potential, several drawbacks must be addressed to completely exploit their capabilities. One of the key limitation of CS-based hydrogels is their relatively low mechanical strength. Hydrogels, in general, exhibit weak mechanical properties, which limit their utility in applications necessitating structural support (e.g., bone and cartilage regeneration). Enhancing the strength of these hydrogels without losing their biocompatibility and injectability represents a significant challenge. Additionally, the regulated release of therapeutic agents is crucial for the success of DDSs. CS-based hydrogels should be engineered to release drugs in a regulated manner, with the release profile affected by various factors (e.g., temperature, pH, and enzyme activity). Achieving predictable and responsive behavior in these hydrogels is an ongoing challenge.

Although CS is usually considered safe for the body, modifications required to create smart hydrogels may provoke undesirable immune responses. Ensuring that these modifications do not induce adverse immune reactions is critical for safely applying these materials in vivo. Furthermore, the long-term biocompatibility and safety of CS-based hydrogels need to be thoroughly investigated. The hydrogel degradation rate needs to be carefully controlled to match the intended application. For example, the hydrogel needs to degrade in TE at a rate that supports tissue regeneration. Balancing the degradation rate with the hydrogel's mechanical properties and functional efficacy remains a substantial challenge. The scalability and reproducibility of CS-based hydrogel production are additional concerns. Manufacturing processes should be optimized to ensure consistent quality at large scales, which is essential for clinical and commercial applications. Advancements in fabrication techniques are necessary to improve reproducibility and facilitate large-scale production.

Several research directions and strategies are emerging to address these challenges. Advanced crosslinking techniques can improve the mechanical characteristics of CS-based hydrogels. Techniques such as ionic crosslinking, covalent bonding, and incorporating NPs can considerably enhance the strength and stability of these hydrogels. Exploring novel crosslinking agents and strategies will be critical to overcoming limitations. Developing hydrogels that respond to external stimuli holds significant promise for more controlled drug release and other functional applications. Utilizing nanotechnology and specialized fabrication techniques (e.g., electrospinning) and hydrogels embedded with nanomaterials can enhance the responsiveness of these systems. Bioprinting technology, in particular, offers innovative approaches for fabricating complex tissue constructs using CS-based hydrogels. This technique enables the precise deposition of hydrogel layers to create intricate tissue architectures, thereby enhancing tissue functionality and integration.

The usage of CS-based hydrogels in personalized medicine is an emerging trend. Tailoring hydrogels for meeting specific requirements of each patient can improve therapeutic outcomes. However, one needs a deeper understanding of the interaction between hydrogels and the biological environment. Regulatory frameworks for the approval of CS-based smart hydrogels are also crucial. Constituting explicit instructions for the safety, biocompatibility, and efficacy of these materials will accelerate their translation into clinical practice. The collaboration of researchers, industry stakeholders, and regulatory agencies will be essential in driving the clinical adoption of these technologies. Therefore, developing cost-effective and environmentally sustainable production methods for CS-based hydrogels is imperative for their widespread adoption. Employing renewable CS sources and optimizing manufacturing processes can reduce costs and minimize environmental impact. Exploring green chemistry techniques for synthesizing these materials will contribute to their sustainable production and minimize the environmental impact.

## 9 Conclusion

CS-SIHs present transformative opportunities for targeted drug delivery, regenerative medicine, and TE, owing to their biocompatibility, biodegradability, and tunable responsiveness. As outlined in this review several technical and translational challenges remain, such as variability in CS sources, mechanical limitations, regulatory complexities, and the challenges of scaling up manufacturing processes. Overcoming these barriers is critical to the successful clinical translation of CS-SIHs. A clear path forward must include the establishment of standardized material specifications, scalable crosslinking methods, integration with bioprinting and nanotechnology, and early alignment with regulatory requirements. The successful clinical translation of CS-SIHs will depend on comprehensive in vivo safety assessments and the initiation of rigorously designed human trials. Furthermore, cross-sector collaboration between biomaterials scientists, clinicians, manufacturers, and regulators will be essential in determining the clinical and commercial success of CS-SIHs. With continuous innovation and translational alignment, CS-SIHs are positioned to transition from promising laboratory tools to clinically viable therapies. This study provides a comprehensive overview of material chemistry, smart response mechanisms, manufacturing methodologies, and clinical advancements in CS-SIHs. Unlike previous reviews that focused on isolated topics, this interdisciplinary approach provides a comprehensive roadmap for biomedical advancements in this field.

## CRediT authorship contribution statement

**Qamar Salamat:** Writing – review & editing, Writing – original draft. **Rasoul Moradi:** Writing – review & editing, Writing – original draft, Supervision, Project administration. **Zahra Nadizadeh:** Writing – review & editing, Writing – original draft. **Pirouz Kavehpour:** Writing – review & editing, Writing – original draft, Validation, Project administration. **Mustafa Soylak:** Writing – review & editing, Writing – original draft, Supervision, Resources. **Ahmet Asimov:** Writing – review & editing, Writing – original draft, Validation, Resources. **Md Zillur Rahman:** Writing – review & editing, Writing – original draft, Visualization, Validation, Resources. **Tomáš Kovářík:** Writing – review & editing, Writing – original draft, Supervision, Resources, Project administration, Funding acquisition. **Václav Babuška:** Writing – review & editing, Validation, Supervision, Resources. **Kalim Deshmukh:** Writing – review & editing, Writing – original draft, Visualization, Validation, Supervision, Resources, Project administration, Conceptualization.

## Ethics approval and consent to participate

This review article does not require any ethical approval or allied consents for publication.

## Declaration of competing interest

The authors declare that they have no known competing financial interests or personal relationships that could have appeared to influence the work reported in this paper.

## Data Availability

The data described in the article are available at: https://doi.org/10.5281/zenodo.15464019.
